# Delineating Life‐Course Percentile Curves and Normative Values of Multi‐Systemic Ageing Metrics in the United Kingdom, the United States, and China

**DOI:** 10.1002/jcsm.13862

**Published:** 2025-06-13

**Authors:** Liming Zhang, Jiening Yu, Xueqing Jia, Zichang Su, Yingying Hu, Jingyun Zhang, Wei Yang, Xi Chen, Emiel O. Hoogendijk, Huiqian Huang, Zuyun Liu

**Affiliations:** ^1^ Second Affiliated Hospital, School of Public Health, Zhejiang Key Laboratory of Intelligent Preventive Medicine Zhejiang University School of Medicine Hangzhou China; ^2^ Zhejiang University School of Medicine Hangzhou China; ^3^ School of Public Affairs Zhejiang University Hangzhou China; ^4^ Department of Epidemiology and Health Statistics School of Public Health, Hangzhou Medical College Hangzhou China; ^5^ Department of Global Health and Social Medicine Institute of Gerontology, King's College London London UK; ^6^ Department of Health Policy and Management Yale School of Public Health New Haven Connecticut USA; ^7^ Department of Economics Yale University New Haven Connecticut USA; ^8^ Department of Epidemiology & Data Science, Amsterdam Public Health Research Institute Amsterdam UMC‐Location VU University Medical Center Amsterdam the Netherlands; ^9^ Basic and Translational Research Center The Second Affiliated Hospital, Zhejiang University School of Medicine Hangzhou China

**Keywords:** cohort study, heterogeneity, multi‐dimensional ageing metric, normative value, percentile curve, sociodemographic disparity

## Abstract

**Background:**

Ageing is a complex and multi‐dimensional process that manifests heterogeneities across different organs/systems, individuals and countries. We aimed to delineate the life‐course percentile curves and establish the normative values of multi‐systemic (e.g., muscle‐skeletal, brain, cardiovascular and pulmonary) ageing metrics for people under distinct sociodemographic contexts (i.e., sex, income and education).

**Methods:**

Three national datasets, the UKB (the United Kingdom), the NHANES (the United States) and the CHARLS (China) were utilized for the analyses. We selected 14 ageing metrics (e.g., body mass index, grip strength, fat‐free mass index, bone mineral content [BMC], bone mineral density [BMD], diastolic blood pressure, cognitive function and frailty index_Lab) that represent the functions of different organs/systems and plotted their sex‐, educational‐ and income‐specific percentile curves utilizing the GMALSS model. We also estimated the age‐specific normative values for each ageing metric in distinct sociodemographic contexts.

**Results:**

The functions of all metrics, except for cognitive function, manifested a progressive decline or maintained stability after adulthood (20s), especially after middle age (40s–50s). The cognitive function showed an evident decline in old age (70s–75s) (e.g., in the CHARLS: the median [IQR] cognitive function scores were 11.6 [9.1, 13.8], 10.3 [7.5, 12.9], 8.3 [5.5, 11.0] at the ages of 60, 70 and 80 for males, respectively). In the stratified analyses, males and females manifested disparities in percentile curves of ageing metrics involving the muscle‐skeletal and cardiovascular systems. For instance, BMC and BMD manifested an evident decline after middle age in females, whereas they showed a slow decline after adulthood in males. Notably, we observed substantial income and educational disparities in percentile curves of several ageing metrics within Chinese participants: the ‘low‐income’ and ‘low‐education’ subgroups manifested an evident decline in ageing metrics (e.g., grip strength and frailty index_Lab) representative of multiple systems. By contrast, these income or educational disparities were not observed in the British and American participants.

**Conclusions:**

Our investigation delineated the potential heterogeneities and socioeconomic disparities in percentile curves of multi‐systemic ageing metrics and provided their age‐specific normative values tailored to different sexes and socioeconomic contexts based on three national datasets. This study may serve as a proof‐of‐concept for understanding the multi‐dimensional signature of systemic ageing and calls for policies to promote health equity across nations when facing dramatic global ageing.

## 1 Introduction

Ageing, characterized by a progressive deterioration in physiological integrity and fitness, is a complex and multi‐dimensional process [1, 2]. To shed light on ageing holistically, several single biomarkers [3] (e.g., grip strength, muscle mass and body composition indicators) and various composite ageing metrics (e.g., frailty index [4]) have been developed and utilized. Given the potential variation in ageing rates across organs/systems, relying on a single or limited number of metric(s) may fail to capture the multi‐dimensional signature of ageing comprehensively. For instance, the decline in cognitive function may not occur in parallel to the decrease in muscle mass [5, 6]. Consequently, carrying out geroscience research from a multi‐dimensional perspective is paramount [1, 7, 8, 9].

Ageing processes not only differ between organs/systems but also vary across the whole life course, exhibiting heterogeneities across individuals [10] and even countries [11]. These heterogeneities could be attributed to the combined impacts of multiple determinants, such as genetics, lifestyle, and socioenvironmental circumstances [12]. Genetic factors, which are less modifiable, undoubtedly play an essential role [13]. By contrast, sociodemographic factors, among which sex, economic position and education manifest disparities across different countries and regions, potentially play a more critical role in contributing to ageing heterogeneities [12]. These factors are potentially amenable to intervention through policy implementation, making them crucial in narrowing health inequalities.

Although current studies have attempted to utilize large‐sample datasets from diverse ethnic groups to map the life‐course changes (e.g., the percentile curves) of different ageing metrics (e.g., grip strength and skeletal mass index) and provide their normative values [14, 15], they may not fully address the heterogeneity observed in ageing processes across different systems, ethnicities and socioeconomic contexts. In light of the dramatic global ageing trend, delineating the life‐course changes of multi‐systemic ageing metrics and deciphering their potential sociodemographic disparities are indispensable prerequisites for evidence‐informed policies and programs, ultimately contributing to a reduction in public health burden.

To fulfil the potential of this work in public health, we performed a cross‐national study with the aim of delineating the percentile curves of multi‐systemic ageing metrics for people in the United Kingdom (UK), the United States (US) and China, three large countries with distinct sociodemographic contexts (Figure S1a) and estimate the age‐specific normative values for each ageing metric. Figure S1b–e presents the roadmap of this study.

## 2 Methods

Detailed descriptions of the study population, measurements of multi‐systemic ageing metrics, sociodemographic factors, and statistical analyses are provided in the Supporting Information.

### 2.1 Study Population

Participants from three national datasets, the UK Biobank (UKB, 2014 wave), the US National Health and Nutrition Examination Survey (NHANES, 2011–2020 wave) and the China Health and Retirement Longitudinal Study (CHARLS, 2015 wave), with valid data were included (Figure S1b). The initial enrollment comprised 80 791, 45 462 and 21 789 participants in UKB, NHANES and CHARLS, respectively. After excluding participants with missing data on age and all ageing metrics, those who were pregnant or had prevalent cancer during the survey (*N* = 37 090, 8117 and 6457 in UKB, NHANES and CHARLS, respectively), the analysis ultimately encompassed 43 701, 37 345 and 15 332 participants from the three datasets, respectively. Statistically significant differences were observed in the distribution of several basic characteristics (e.g., age and sex) between the excluded and included participants across three datasets (Tables S1–S3).

### 2.2 Sociodemographic Factors

Three sociodemographic factors (sex, income and education) were considered in the current analyses (Figure S1c). Sex was divided into male and female. Both income and education were classified into ‘low’ and ‘high’ subgroups, according to their corresponding criteria (see Supporting Information).

### 2.3 Measurements of Multi‐Systemic Ageing Metrics

Relying on the available data we acquired, we selected 13 ageing metrics that can capture the functions of different organs/systems (i.e., brain, metabolic, cardiovascular, muscle‐skeletal and pulmonary) and dimensions, encompassing *Mental health* (including cognitive function and depression) and *Physical health* (including body mass index [BMI], waist circumference [WC], systolic blood pressure [SBP], diastolic blood pressure [DBP], pulse, grip strength, peak expiratory flow [PEF], forced expiratory volume in the first second [FEV_1_], fat‐free mass index [FFMI], bone mineral content [BMC] and bone mineral density [BMD]).

Additionally, we incorporated a composite ageing metric derived from blood biomarkers, the frailty index_Lab (FI_Lab), to provide a more integrated representation of ageing and physical health from a multi‐systemic perspective (Figure S1d). All these ageing metrics were available in at least two of the three datasets (Table S4).

### 2.4 Statistical Analysis

Basic characteristics for participants in total and different sociodemographic contexts were reported as median with the interquartile range (IQR). The Mann–Whitney *U* test and Cohen's d statistics were performed to compare participants' basic characteristics across different subgroups. Furthermore, the Generalized Additive Models for Location, Scale, and Shape (GAMLSS) method [16] (R package ‘gamlss’) was used to fit the smoothed percentile curves of the 14 ageing metrics. We employed several tests and/or parameters to evaluate the fitting degree and applicability of the models, including the Akaike Information Criterion (AIC), against fitted values, density estimates, normal quantile–quantile (*Q*–*Q*) plots and fitted percentile curves. Notably, for the analysis of each ageing metric, we refitted the GAMLSS models across different datasets and within subgroups by various sociodemographic characteristics, to select the most suitable parameters and identify the optimal fitting model, thereby ensuring the robustness and validity of the results across diverse datasets. The optimal models were ultimately determined based on the Exponential (EXP) distribution for depression, the Gamma distribution for FI_Lab and the Box‐Cox t (BCT) distribution for the remaining ageing metrics across all three datasets, respectively. Utilizing the best‐fitted model, we generated sex‐, income‐ and education‐specific percentile curves for each ageing metric and estimated their age‐specific normative values for the 1st, 5th, 25th, 50th, 75th, 95th and 99th percentiles (Figure S1e). Notably, several chronic diseases (e.g., memory dysfunctions and arthritis) were excluded during the analysis of several ageing metrics (e.g., cognitive functions and grip strength) to avoid potential bias. All statistical analyses were performed using R software (Version 4.2.2), and a two‐tailed *p* value < 0.05 was considered statistically significant.

## 3 Results

### 3.1 Basic Characteristics of Participants

As illustrated in Table S5, the analyses included 15 332, 37 345 and 43 701 participants in CHARLS (median age: 60.0 years; males: 47.7%), NHANES (median age: 29.0 years; males: 49.8%) and UKB (median age: 64.8 years; males: 48.6%), respectively.

In CHARLS, males exhibited significantly higher grip strength and PEF compared to females (Cohen's *d* > 0.8; *p* < 0.05). Participants in the ‘high‐income’ and ‘high‐education’ groups exhibited higher values across several ageing metrics (e.g., cognitive functions, grip strength and PEF) compared to their respective ‘low‐income’ and ‘low‐education’ counterparts. In NHANES, males exhibited higher values in ageing metrics representative of muscle‐skeletal and pulmonary systems (e.g., FEV1 and BMC) compared to females. Participants in the ‘high‐income’ and ‘high‐education’ groups demonstrated statistically significant differences across most ageing metrics compared to their respective ‘low‐income’ and ‘low‐education’ counterparts, despite the Cohen's *d* statistics indicating a limited effect size for these differences. In UKB, we observed results that were consistent with those found in NHANES.

### 3.2 Percentile Curves and Normative Values for Multi‐Systemic Ageing Metrics

**FIGURE 1:**
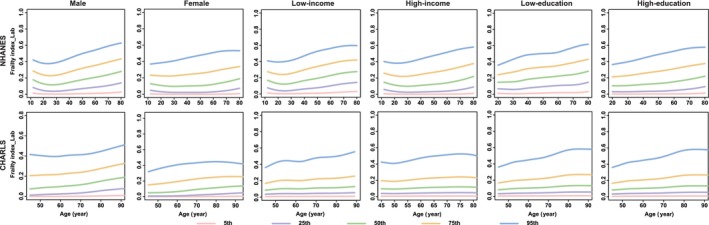
Percentile curves of frailty index_Lab by different sociodemographic contexts in CHARLS and NHANES. *Note:* The solid lines in different colours represent the corresponding percentile curves. NHANES, the National Health and Nutrition Examination Survey; CHARLS, the China Health and Retirement Longitudinal Study.

**FIGURE 2:**
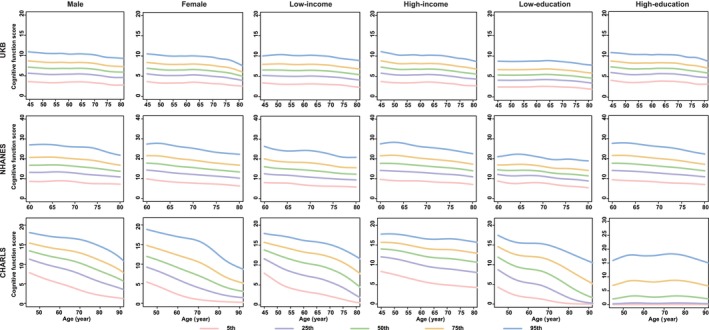
Percentile curves of cognitive function score by different sociodemographic contexts in three datasets. *Note:* The solid lines in different colours represent the corresponding percentile curves. UKB, the UK Biobank; NHANES, the National Health and Nutrition Examination Survey; CHARLS, the China Health and Retirement Longitudinal Study.

**FIGURE 3:**
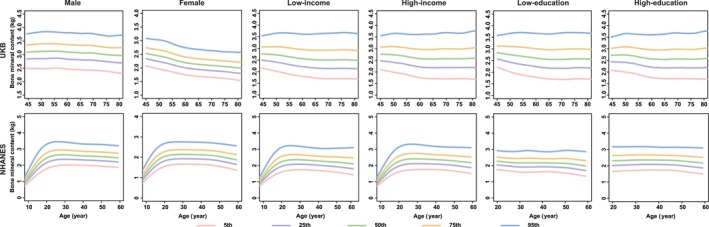
Percentile curves of body mineral content by different sociodemographic contexts in UKB and NHANES. *Note:* The solid lines in different colours represent the corresponding percentile curves. UKB, the UK Biobank; NHANES, the National Health and Nutrition Examination Survey.

**FIGURE 4:**
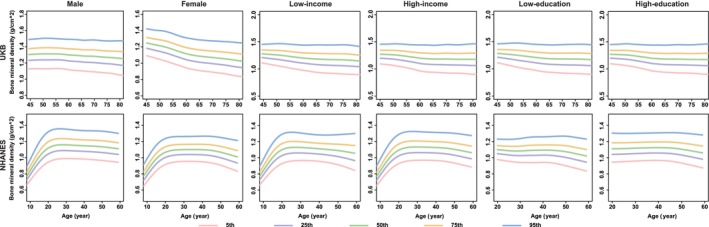
Percentile curves of body mineral density by different sociodemographic contexts in UKB and NHANES. *Note:* The solid lines in different colours represent the corresponding percentile curves. UKB, the UK Biobank; NHANES, the National Health and Nutrition Examination Survey.

**FIGURE 5:**
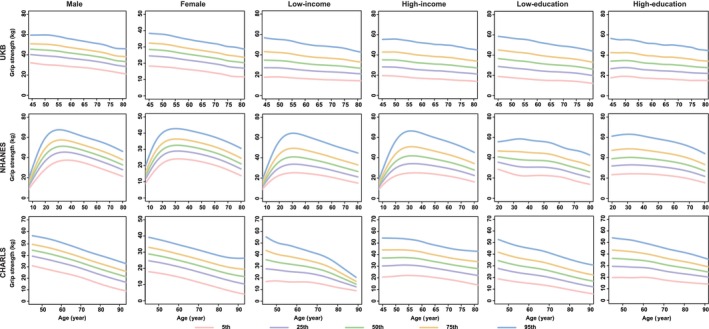
Percentile curves of grip strength by different sociodemographic contexts in three datasets. *Note:* The solid lines in different colours represent the corresponding percentile curves. UKB, the UK Biobank; NHANES, the National Health and Nutrition Examination Survey; CHARLS, the China Health and Retirement Longitudinal Study.

**FIGURE 6:**
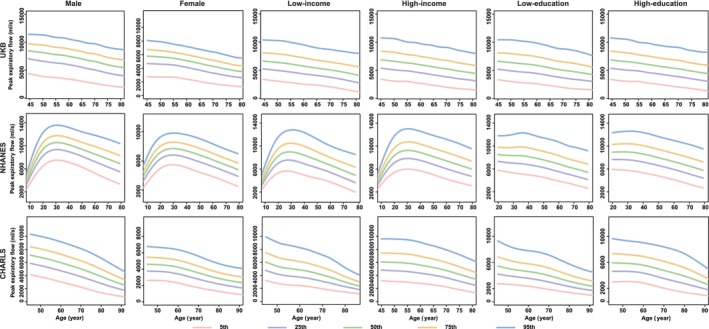
Percentile curves of peak expiratory flow by different sociodemographic contexts in three datasets. *Note:* The solid lines in different colours represent the corresponding percentile curves. UKB, the UK Biobank; NHANES, the National Health and Nutrition Examination Survey; CHARLS, the China Health and Retirement Longitudinal Study.

Generally, the functions of all ageing metrics (except for cognitive function) manifested a progressive decline (e.g., the decrease in BMI, grip strength, PEF, FEV_1_, the increase in SBP and FI_Lab) or maintained stability (e.g., WC for females) with advancing age after adulthood (20s), especially after middle age (e.g., DBP for males; BMC and BMD for females) (Figures 1, 3, 4, 5, 6 and [Link], [Link], [Link], [Link], [Link], [Link], [Link], [Link]). The cognitive function showed an evident decline in old age (70s–75s), especially in the Chinese (in the CHARLS: for males, the median (IQR) cognitive function scores were 11.6 (9.1, 13.8), 10.3 (7.5, 12.9) and 8.3 (5.5, 11.0) at the ages of 60, 70 and 80, respectively; for females, the median (IQR) cognitive function scores were 9.1 (6.1, 12.3), 7.1 (4.0, 10.6) and 4.8 (2.4, 7.6) at the age of 60, 70 and 80, respectively) and British (in the UKB: for males, the median [IQR] cognitive function scores were 6.8 [5.4, 8.3], 6.6 [5.2, 8.1], 5.9 [4.6, 7.3] at the age of 60, 70 and 80, respectively; for females, the median [IQR] cognitive function scores were 6.6 [5.2, 7.9], 6.3 [5.0, 7.7], 5.2 [4.1, 6.3] at the age of 60, 70 and 80, respectively) participants (Figure 2). The age‐specific normative values of the ageing metrics under different sociodemographic contexts are shown in Tables S7–S12.

### 3.3 Disparities of Percentile Curves for Ageing Metrics Under Different Sociodemographic Contexts in Three Countries

In the sex‐stratified analyses, males and females demonstrated disparities in the percentile curves of ageing metrics involving the cardiovascular and muscle‐skeletal systems. For instance, BMC and BMD manifested an evident decline after middle age (40s–50s) in females. However, the two ageing metrics showed a slow decline in males after adulthood (Figures 3 and 4). Additionally, DBP increased after middle age in males, while it maintained stable in females, especially in the British and Chinese participants (Figure  S6).

Notably, we observed substantial disparities in percentile curves of ageing metrics for participants according to income and educational levels within Chinese participants. Specifically, the ‘low‐income’ and ‘low‐education’ subgroups manifested evident declines in functions of the brain (i.e., cognitive function and depression), muscle‐skeletal (i.e., grip strength) and pulmonary (i.e., PEF) systems, as well as physical health (i.e., FI_Lab), compared to their higher‐income or higher‐education counterparts (Figures 1 and 2, 5 and 6 and S8). However, these income or educational disparities were non‐existent among the British and American participants.

## 4 Discussion

Comprehending the multi‐dimensional signature of ageing constitutes a high priority for carrying out geroscience research. In this study where we delineated the percentile curves of multi‐systemic ageing metrics for persons under different sociodemographic contexts in the United Kingdom, the United States and China, we observed heterogeneities across different organs/systems (i.e., the decline of cognitive function was evident in old age, compared to the progressive decline of the other metrics after adulthood), informing the promise of system‐targeted interventions and therapies at the key age node to prevent ageing‐related diseases such as cognitive decline, fracture, and sarcopenia. Additionally, we observed disparities in percentile curves of ageing metrics under different sociodemographic contexts in three countries, implying the urgent need to improve evidence‐based anti‐ageing policies, particularly for developing countries, to narrow health inequalities caused by regional socioeconomic disparities. Moreover, the established age‐specific normative values facilitate the evidence‐based criteria intended to identify people with chronic diseases or poor health conditions. In light of the dramatic global ageing trend, our study may provide clues for evidence‐based policies for narrowing cross‐national health inequalities attributed to regional socioeconomic disparities and serve as a proof‐of‐concept for understanding the multi‐dimensional and heterogeneous nature of systemic ageing, ultimately promoting health span and longevity.

The comprehension of ageing has matured from a uni‐dimensional perspective to a sophisticated and multi‐dimensional paradigm [1], which unfortunately remains overlooked by the majority of current geroscience research. Ingeniously, the ageing metrics selected from these dimensions can partially represent the functions of different organ systems, including muscle‐skeletal (i.e., grip strength and FFMI), brain (i.e., cognitive function) and pulmonary (e.g., FEV_1_) system, enabling the intuitive observation of the intricate ageing patterns across different organs/systems. Simultaneously, these metrics may provide insights into the ageing process from both anatomical and physiological perspectives, facilitating a more intuitive assessment of the ageing process [17]. In addition to single ageing metrics, we incorporated a composite ageing metric, FI_Lab, based on blood biomarkers, which captures more comprehensive aspects of ageing from a multi‐systemic perspective [18, 19]. Both FI_Lab itself and its constituent biomarkers (e.g., creatinine) were demonstrated to capture a broader spectrum of molecular ageing signatures [20, 21], making the FI_Lab integrate ageing information across multiple dimensions encompassing functional, physiological, and molecular levels.

Unsurprisingly, the percentile curves of the ageing metrics present analogous patterns across most systems involving the cardiovascular, pulmonary, and muscle‐skeletal systems, which manifest a progressive decline in functions after adulthood. The findings signify a relative harmony of systemic ageing and the benefit of early prevention in delaying disease onset and promoting an extended healthy lifespan. However, certain systems displayed a unique pattern. Cognitive function showed an evident decline after old age in the Chinese and British populations, implying that brain ageing may not be entirely aligned, or at least may not be synchronized with other systems, despite considering the influences of the dynamic interactions within different organ systems. Tian et al. [10] have indicated that there were marked heterogeneities in organ‐specific biological ages, and an organ's biological age merely selectively affects the ageing rates of several connected organ systems, rather than formulating a holistic network across the whole organism. The above findings shed light on the potential clinical implications of implementing system‐targeted interventions and therapies before the key age node for delaying the onset of specific diseases (e.g., initiating early diagnosis/prevention of cognitive impairment and Alzheimer's disease in middle age rather than in elderly stages). Nevertheless, we call caution that distinguished from insights derived from longitudinal trajectories of ageing metrics, which track the dynamics within the same individuals over time, the percentile curves generated from our large‐sample cross‐sectional data provide a complementary population‐level perspective, illustrating the distribution of ageing metrics at any specific time points and are instrumental in elucidating patterns and variability within the same population.

To investigate the potential disparities of percentile curves, we conducted stratified analyses based on sex, education and income status. There were no intuitive disparities in percentile curves between males and females across most of the ageing metrics. However, the decline of muscle‐skeletal functions (e.g., BMC and BMD) was intuitively more pronounced in females compared to males, particularly after middle age. This phenomenon may be attributed to the reduced bone content and altered bone tissue structures caused by decreased oestrogen levels after perimenopause [22], highlighting targeted prevention strategies for high‐risk populations. For instance, increasing calcium intake to prevent osteoporosis and reduce fracture and sarcopenia risks in perimenopausal females.

Prior studies have attempted to investigate the percentile curves of the muscle‐skeletal systemic ageing metrics (e.g., grip strength and skeletal muscle index) and established corresponding normative values [14, 15]. Nevertheless, as pinpointed in these studies, they may not sufficiently address the scientific challenges posed by ethnic heterogeneity and the potential influence of varying socioeconomic contexts. Moreover, they may fail to fully capture the heterogeneity of systemic ageing, despite muscle ageing being a crucial dimension of the broader ageing process. Healthy ageing, particularly in terms of muscle health and functional capacity, is influenced by the dynamic interplay of multiple factors, including age, sex, nutritional status and genetic background [23, 24, 25]. By establishing age‐specific normative values tailored to different sexes and socioeconomic contexts using data from three large‐sample national datasets, we provide more applicable and precise tools for assessing physical health conditions for people in different countries and sociodemographic contexts.

Notably, in China, participants with lower educational levels and income status exhibited an intuitively more pronounced decline in cognitive function, compared to their higher counterparts. The findings underscore the cumulative detrimental effects of educational and economic disadvantages on the overall health of the Chinese population, consistent with reports by others [5]. These socioeconomic disparities present a critical challenge for many developing countries, necessitating imperative attention and actions.

Several reasons may lie behind the disparities in percentile curves of ageing metrics in three countries. First, China is experiencing rapid health transition and socioeconomic development, whereas the United States and United Kingdom have already passed through similar stages several decades ago. Consequently, the long‐term accumulation of multiple adversities, particularly subpar educational attainment and economic status, further exacerbates these health inequality manifestations. Second, the differences in health policies, welfare institutions, and cultural backgrounds between the three countries may also contribute to these disparities. This calls for policy interventions aimed at reducing economic/educational disparities as a means to address and mitigate health inequalities, especially for many developing countries. China has steadfastly devoted itself to reform initiatives aimed at narrowing educational and economic disparities (e.g., ‘progressive taxation’ and ‘nine‐year compulsory education’) and implementing proactive policies to improve the life quality (e.g., ‘Critical Illness Insurance’) for its population. In the future, these efforts are expected to yield positive impacts, ultimately promoting global health equity.

This study may provide the following valuable contributions. First, based on the evidence from large‐scale data analyses, we have emphasized a widely acknowledged yet often overlooked scientific question that ageing is a complex and multi‐dimensional process. Future investigations necessitate moving beyond a uni‐dimensional and uni‐systemic perspective towards a multi‐dimensional and multi‐systemic perspective to comprehend a more holistic landscape of ageing. We hope these empirical findings may facilitate the development of system‐targeted interventions aimed at delaying age‐related diseases. Second, from a cross‐national standpoint, our study revealed the disparities in percentile curves of ageing metrics within and across different countries under varying sociodemographic contexts. This provided a paradigm for performing cross‐national ageing investigations. The thought‐provoking findings underscore the adverse impacts of educational and economic disparities on ageing, which call for evidence‐based policies aimed at narrowing health inequality. Third, the established normative values, derived from the large sample of relatively healthy adults, can serve as baseline data and evidence‐based criteria for evaluating interventions and provide early predictive markers for specific diseases, such as sarcopenia, as well as other functional decline and metabolic disorders.

Nevertheless, we acknowledge the potential limitations. First, the ageing metrics we outlined may be arbitrary to some extent and may overlook other crucial dimensions of ageing (e.g., molecular ageing), although this analysis incorporated the FI_Lab. However, large population‐based cohorts that include molecular ageing phenotypes are scarce. Additionally, due to the confidentiality and restricted access of these datasets, it was not feasible to incorporate these molecular ageing metrics into our current analysis. Our future research will be based on longitudinal multi‐omics data, as exemplified by the ZheJiang longitudinAl Study of Healthy Aging (JASHA) [26], an ongoing cohort that incorporates longitudinal multi‐dimensional ageing phenotypes (e.g., anthropometric and physical function indicators, blood biochemical indicators, body composition, brain CT/MRI imaging data and multiomics data), to elucidate more molecular insights and biological mechanisms of ageing. Second, the cross‐sectional nature of the analysis limited the ability to understand the potential causal relationships between socioeconomic disparities and poor health conditions. These correlations may be bidirectionally causative, with other factors potentially acting as mediators (for instance, low income may limit access to healthcare, thereby contributing to poor health conditions). Future longitudinal studies combined with appropriate methodologies, such as structural equation modelling and mediation analysis, hold promise for addressing these uncertainties. Third, it is crucial to acknowledge that ageing is a complex process influenced by the combined effects of multiple factors. In our study, we only examined a limited set of sociodemographic factors that are potentially amenable to intervention through policy implementation, warranting further endeavours to explore the impact of other determinants, as well as their interactions, on ageing. Fourth, we adopted a binary categorization of education and income, dividing these variables into only two subgroups. This approach inevitably oversimplifies the intricate socioeconomic gradients, which may obscure critical nuances, especially within nations marked by pronounced wealth disparities (e.g., China and the United States), necessitating future investigations. Fifth, to accurately depict the life‐course percentile curve of the ageing metrics, we did not exclude older adults in the analysis, which may introduce survival bias and ultimately underestimate the actual decline rate of ageing metrics within this susceptible population, particularly in nations characterized by elevated mortality rates (e.g., China and the United States). Sixth, given the present research objectives, the GAMLSS model stands out as the most appropriate methodology. Nevertheless, this model is limited in its capacity to identify precise time points of age‐related fluctuations, compare statistical differences in percentile curves among various subgroups, and incorporate covariates alongside their interaction terms within the same model. Seventh, the assessment methodologies for certain ageing metrics (e.g., cognitive function and depression) vary across different datasets, posing challenges for direct comparisons and necessitating more rigorous cross‐cultural validations. Lastly, the strict inclusion and exclusion criteria in the analysis may introduce selection bias.

## 5 Conclusion

Our investigation delineated the heterogeneities and socioeconomic disparities in percentile curves of multi‐systemic ageing metrics and provided their age‐specific normative values tailored to specific sexes and socioeconomic contexts based on three national datasets. This work may serve as a proof‐of‐concept for understanding the multi‐dimensional signature of systemic ageing and call for policies to promote health equity across nations when facing dramatic global ageing.

## Ethics Statement

The CHARLS is approved by the Biomedical Ethics Review Committee of Peking University (No. IRB00001052–11015), and all participants provide informed consent. The NHANES is approved by the National Center for Health Statistics Research Ethics Review Board (Protocol #2011‐17; Continuation of Protocol #2011‐17; and Protocol #2018‐01), and all participants provide informed consent. The UKB was approved by the North West Multi‐centre Research Ethics Committee (11/NW/0382) and all participants provide informed consent.

## Conflicts of Interest

The authors declare no conflicts of interest.

## Supporting information


**Data S1** Supplementary Information.

**Table S1.** Basic characteristics of the excluded and included participants in CHARLS. **Note:** Basic characteristics were expressed as mean±standard deviation and number with percentage for continuous and categorical variables, respectively. CHARLS, the China Health and Retirement Longitudinal Study.
**Table S2.** Basic characteristics of the excluded and included participants in NHANES.Note: Basic characteristics were expressed as mean±standard deviation and number with percentage for continuous and categorical variables, respectively. NHANES, the National Health and Nutrition Examination Survey.
**Table S3.** Basic characteristics of the excluded and included participants in UKB.Note: Basic characteristics were expressed as mean±standard deviation and number with percentage for continuous and categorical variables, respectively. UKB, the UK Biobank.
**Table S4.** Multi‐systemic ageing metrics available from the three datasets. Note: FEV_1_, forced expiratory volume in the first second; “√” represent the variables are available in this dataset; CHARLS, the China Health and Retirement Longitudinal Study; NHANES, the National Health and Nutrition Examination Survey; UKB, the UK Biobank.
**Table S5.** Biomarkers utilized for the construction of frailty index_Lab and the corresponding cut‐off values in CHARLS and NHANES, respectively. Note: CHARLS, the China Health and Retirement Longitudinal Study; NHANES, the National Health and Nutrition Examination Survey.
**Table S6.** Basic characteristics for participants in total and by different sociodemographic contexts in three datasets. Note: Basic characteristics were expressed as median with the inter‐quartile ranges. The Mann–Whitney U‐test and Cohens'd statistics were used for the comparison for the participants’ characteristics across different subgroups; FEV1, forced expiratory volume in the first second; CHARLS, the China Health and Retirement Longitudinal Study; NHANES, the National Health and Nutrition Examination Survey; UKB, the UK Biobank.
**Table S7**. Age‐specific percentile values of multi‐systemic ageing metrics for males in three datasets. Note: FEV_1_, forced expiratory volume in the first second; CHARLS, the China Health and Retirement Longitudinal Study; NHANES, the National Health and Nutrition Examination Survey; UKB, the UK Biobank.
**Table S8.** Age‐specific percentile values of multi‐systemic ageing metrics for females in three datasets. Note: FEV_1_, forced expiratory volume in the first second; CHARLS, the China Health and Retirement Longitudinal Study; NHANES, the National Health and Nutrition Examination Survey; UKB, the UK Biobank.
**Table S9.** Age‐specific percentile values of multi‐systemic ageing metrics for participants in the “low‐income” group in three datasets. Note: FEV_1_, forced expiratory volume in the first second; CHARLS, the China Health and Retirement Longitudinal Study; NHANES, the National Health and Nutrition Examination Survey; UKB, the UK Biobank.
**Table S10.** Age‐specific percentile values of multi‐systemic ageing metrics for participants in the “high‐income” group in three datasets. Note: FEV_1_, forced expiratory volume in the first second; CHARLS, the China Health and Retirement Longitudinal Study; NHANES, the National Health and Nutrition Examination Survey; UKB, the UK Biobank.
**Table S11.** Percentile values of multi‐systemic ageing metrics for participants in the “low‐education” group in three datasets. Note: FEV_1_, forced expiratory volume in the first second; CHARLS, the China Health and Retirement Longitudinal Study; NHANES, the National Health and Nutrition Examination Survey; UKB, the UK Biobank.
**Table S12.** Age‐specific percentile values of multi‐systemic ageing metrics for participants in the “high‐education” group in three datasets. Note: FEV_1_, forced expiratory volume in the first second; CHARLS, the China Health and Retirement Longitudinal Study; NHANES, the National Health and Nutrition Examination Survey; UKB, the UK Biobank.

**Figure S1** Roadmap of the study design. a. Sociodemographic disparities within the United Kingdom, the United States, and China, including the tendencies of the male‐to‐female ratio, the GDP per capita, and the educational attainment rate for lower secondary schools in adults. b. Three national datasets were utilized for the cross‐national analyses, including the UKB (the United Kingdom, 2014 wave, *N* = 43 701), the NHANES (the United States, 2011–2020 waves, *N* = 37 345), and the CHARLS (China, 2015 wave, *N* = 15 332). c. Three sociodemographic (sex, income, and education) factors were considered in the analysis. d. We selected 14 organ/system representative ageing metrics from different dimensions (i.e., *Mental health*, *Physical health*, and *Biomarker*‐based *Composite index*). e. Using the GAMLSS method, we fitted the percentile curves for each ageing metric with the advancing age. Several parameters were used to test the residual and the degree of the fitted model. Note: Data utilized in Figure S1a were obtained from the official website of the World Bank (https://data.worldbank.org.cn/?cid=eap_wechat_worldbank_zh_ext). CHARLS, the China Health and Retirement Longitudinal Study; NHANES, the National Health and Nutrition Examination Survey; UKB, the UK Biobank; GAMLSS, Generalized Additive Models for Location, Scale, and Shape.

**Figure S2** Percentile curves of body mass index by different sociodemographic contexts in three datasets. Note: The solid lines in different colours represent the corresponding percentile curves. UKB, the UK Biobank; NHANES, the National Health and Nutrition Examination Survey; CHARLS, the China Health and Retirement Longitudinal Study.

**Figure S3** Percentile curves of waist circumference by different sociodemographic contexts in three datasets. Note: The solid lines in different colours represent the corresponding percentile curves. UKB, the UK Biobank; NHANES, the National Health and Nutrition Examination Survey; CHARLS, the China Health and Retirement Longitudinal Study.

**Figure S4** Percentile curves of FEV1 by different sociodemographic contexts in UKB and NHANES. Note: The solid lines in different colours represent the corresponding percentile curves. UKB, the UK Biobank; NHANES, the National Health and Nutrition Examination Survey; FEV1, forced expiratory volume in the first second.

**Figure S5** Percentile curves of systolic blood pressure by different sociodemographic contexts in three datasets. Note: The solid lines in different colours represent the corresponding percentile curves. UKB, the UK Biobank; NHANES, the National Health and Nutrition Examination Survey; CHARLS, the China Health and Retirement Longitudinal Study.

**Figure S6** Percentile curves of diastolic blood pressure by different sociodemographic contexts in three datasets. Note: The solid lines in different colours represent the corresponding percentile curves. UKB, the UK Biobank; NHANES, the National Health and Nutrition Examination Survey; CHARLS, the China Health and Retirement Longitudinal Study.

**Figure S7** Percentile curves of pulse by different sociodemographic contexts in three datasets. Note: The solid lines in different colours represent the corresponding percentile curves. UKB, the UK Biobank; NHANES, the National Health and Nutrition Examination Survey; CHARLS, the China Health and Retirement Longitudinal Study.

**Figure S8** Percentile curves of depression scores by different sociodemographic contexts in CHARLS and NHANES. Note: The solid lines in different colours represent the corresponding percentile curves. NHANES, the National Health and Nutrition Examination Survey; CHARLS, the China Health and Retirement Longitudinal Study.

**Figure S9** Percentile curves of fat‐free mass index by different sociodemographic contexts in UKB and NHANES. Note: The solid lines in different colours represent the corresponding percentile curves. UKB, the UK Biobank; NHANES, the National Health and Nutrition Examination Survey.

## Data Availability

All original data are available through the official website of the CHARLS (http://charls.pku.edu.cn/en/), the NHANES (https://www.cdc.gov/nchs/nhanes/index.htm) and the UKB (https://biobank.ndph.ox.ac.uk/ukb/index.cgi) (under Application Number 61856).
