# Supplementary material for: Delineating Life‐Course Percentile Curves and Normative Values of Multi‐Systemic Ageing Metrics in the United Kingdom, the United States, and China
Source: J Cachexia Sarcopenia Muscle. 2025 Jun 13;16(3):e13862. doi: 10.1002/jcsm.13862 (PMC12163542; doi:10.1002/jcsm.13862)
Supplement: Supplementary file 1 — Data S1 Supplementary Information. [file JCSM-16-e13862-s006.docx]

**Supporting information-Supplementary Methods**

**Study Population**

Participants from three countries, China, the US, and the UK, which have distinct economic, social, and cultural backgrounds, were included. Three national datasets, the China Health and Retirement Longitudinal Study (CHARLS), the National Health and Nutrition Examination Survey (NHANES), and the UK Biobank (UKB), were utilized for subsequent analyses. Comprehensive data of participants was gathered through questionnaires, interviews, laboratory tests, physical examinations, etc. Detailed descriptions for these datasets can be obtained from the corresponding websites and in additional publications[1-3]. In order to maximize the acquisition of multidimensional aging metrics, we selected specific waves for data from each database: the 2015 wave for CHARLS, the 2011-2020 waves for NHANES, and the 2014 wave for UKB. Initially, a total number of 80791, 45462, and 21789 participants were enrolled in the respective survey waves in UKB, NHANES, and CHARLS, respectively. After excluding participants who had missing data on age and all aging metrics, were pregnant, or had prevalent cancer during the survey (N=37090, 8117, and 6457 in UKB, NHANES, and CHARLS, respectively), the analysis ultimately encompassed 43701, 37345, and 15332 participants from the three datasets, respectively (**Fig. S1b**).

**Measurements of multi-systemic aging metrics**

Relying on the available data we acquired, we selected 13 aging metrics representative of different organs/systems and dimensions: *Mental health* (including cognitive function and depression), *Physical health* (including body mass index (BMI), waist circumference (WC), systolic blood pressure (SBP), diastolic blood pressure (DBP), pulse, grip strength, peak expiratory flow (PEF), forced expiratory volume in the first second (FEV_1_), fat-free mass index (FFMI), bone mineral content (BMC), and bone mineral density (BMD)). Additionally, we incorporated a composite aging metric derived from blood biomarkers - the frailty index_Lab (FI_Lab) (**Fig. S1d and Table S4-S5**). Notably, all these aging metrics were available in at least two of the three datasets. The specific measurements for these aging metrics are as follows:

**Cognitive function**

In CHARLS, cognitive function was assessed across three dimensions: orientation and attention (evaluated by the Telephone Interview for Cognitive Status (TICS-10), with scores ranging from 0 to 10), episodic memory (evaluated by the word recall, with scores ranging from 0 to 10), and visual-spatial abilities (evaluated by the figure drawing, with scores ranging from 0 to 1), producing a total score ranging from 0 to 21 [4]. Participants with a higher total score have a better cognitive function. In NHANES, cognitive function was assessed only in the 2011-2014 waves, incorporating three assessments: word learning and recall modules from the Consortium to Establish a Registry for Alzheimer’s Disease (CERAD), the Animal Fluency test, and the Digit Symbol Substitution test (DSST). As these assessments are commonly utilized separately, we opted not to combine their total scores. Ultimately, we selected the Animal Fluency test for evaluating the cognitive function, considering its demand for awareness (e.g., naming animals) irrespective of cultural context, and its reduced reliance on the formal educational experiences of a specific culture[5]. The total score for the Animal Fluency test ranges from 0 to 40, where a higher score represents a better cognitive function. In UKB, cognitive function was assessed using the verbal-numerical reasoning test, namely the Fluid Intelligence (FI) Test [6]. Participants faced 13 verbal logic/reasoning-type multiple-choice questions and were required to answer as many as possible within 2 minutes. Incorrect or unanswered questions were scored as zero, and the total number of correct answers (maximum 13) was used for this analysis.

**Depression**

In CHARLS, depression was assessed using the 10-item Center for Epidemiological Studies Depression Scale (CESD-10), which has been extensively validated in general populations and indicated adequate reliability and validity for the older population in China[7]. Participants were guided to respond to a 10-item questionnaire regarding “how often you felt this way during the past week?”, with a total score ranging from 0 to 30. Participants with a higher score have a worse psychological status. In NHANES, the Patient Health Questionnaire (PHQ)-9[8] was utilized for the assessment of depression. Nine questions on a scale of 0-3 were included, with a total score ranging from 0 to 27. Participants may have a worse psychological status accompanied by a higher score.

**Obesity**

Two obesity indicators, BMI and WC, were available in all three datasets. BMI (in kg/m^2^) was calculated by the quotient for weight (in kg) and the square for height (in m). WC was measured by a soft tape rule in the unit of cm.

**Blood pressure and pulse**

Both SBP (in mmHg) and DBP (in mmHg) were measured using a sphygmomanometer for at least three times, and the mean values were utilized for this analysis. Meanwhile, the pulse rate was also measured during the same procedure or using a pulse trace monitor.

**Grip strength**

Grip strength was measured using a handgrip dynamometer. Participants were instructed to use one of the hands to squeeze the dynamometer as hard as possible, and the test was then repeated for the other hand. Each hand was tested at least two times, and the maximum value for the two hands was ultimately utilized.

**Pulmonary functions**

In CHARLS, pulmonary function was assessed by PEF, which is considered a robust indicator for general health and a predictor of mortality[9]. PEF was measured by trained technicians using a peak flow meter (Everpure™, Shanghai, China) with a disposable mouthpiece, measured in units of L/min. Participants were instructed to stand up, take a deep breath, place their lips around the mouthpiece, and blow as hard and fast as possible. The average value for the three repeated measurements was utilized. In NHANES, PEF (in ml/s) and FEV_1_ (in ml) were measured by a spirometry test, after excluding participants who had current chest pain or a physical problem, etc. Participants were selected for a second spirometry test if their baseline spirometry values indicated possible airflow obstruction. Therefore, the highest value within the two measurements was finally utilized. Notably, we also excluded the readings with quality grades C and D, to ensure the reliability of the data, in accordance with the guidelines and previous research [10]. In UKB, participants underwent a similar testing procedure for the measurement of PEF (in L/min) and FEV_1_ (in L) in the assessment center, after excluding participants with any contraindications including chest infection, detached retina, heart attack, or surgery to the eyes, etc. We carried out a unified conversion for all data units (ml/s for PEF and ml for FEV_1_) to ensure the comparability of data.

**Body composition**

Three body composition indicators, including FFMI (in kg/m^2^), BMC (in kg), and BMD (in g/cm^2^), can be acquired in the NHANES and UKB. In NHANES, body compositions were measured using Dual-energy x-ray absorptiometry (DXA), which is a widely accepted method due in part to its speed, ease of use, and low radiation exposure[11]. FFMI (in kg/m^2^) was obtained by dividing the fat-free mass (in kg) by height (in m). In UKB, the three indicators were also measured using the DXA method following a similar procedure.

**FI_Lab calculation**

By employing laboratory biomarkers, we constructed the FI_Lab based on the identification and accumulation of clinically detectable health deficits across multiple systems, to comprehensively quantify frailty. In the current study, 12 biomarkers (**Table S6**) co-detected in CHARLS and NHANES were included for the construction of FI_Lab, containing blood urea nitrogen, C-reactive protein, creatinine, high-density lipoprotein, glucose, glycohemoglobin, hemoglobin, platelet, total cholesterol, triglyceride, uric acid, and white blood cell. Each biomarker was regarded as a “deficit” when exceeding the normal ranges [12-14](**Table S5**), and the FI_Lab was calculated by counting the number of deficits in an individual and dividing by the total number of deficits (N=12) to produce a score between 0 and 1; a higher score indicates greater frailty.

**Sociodemographic factors**

Three sociodemographic factors were considered in the current analyses, including sex, income, and education (**Fig. S1c**). Sex was divided into male and female uniformly across the three datasets. In CHARLS, the income status was evaluated by annual household income. Following previous studies, participants were divided into quintile groups according to their annual household income, and the lowest quintile group was classified as the “low-income” group in the current study [15]. The rest of the participants were classified as the “high-income” group. In NHANES, the income status was reflected by the ratio of family income to poverty (PIR), which was calculated by dividing family (or individual) income by the poverty guidelines specific to the survey year. Participants with a PIR value less than 1, accounting for 27.5% of the total participants, were regarded as living in poverty, as referred to in the guidelines[16]. In UKB, the income status was assessed by the Townsend deprivation index (TDI), which was a widely used index to evaluate deprivation from four dimensions: unemployment, overcrowded household, non-car ownership, and non-home ownership, participants with a higher TDI score representing higher levels of deprivation. In the current study, participants were regarded as having a worse economic status (the “low-income” group, accounting for 22.2% of the total participants) when their TDI values were higher than 0, as referred to in previous studies[17]. Additionally, participants were classified as the “low-education” group when their highest education level was secondary school or below in all three databases.

**Statistical analysis**

Basic characteristics for participants in total and different sociodemographic contexts were reported as median with the interquartile range (IQR). The Mann-Whitney U-test and Cohen’s d statistics were performed to compare the participants’ basic characteristics across different subgroups.

Furthermore, the Generalized Additive Models for Location, Scale, and Shape (GAMLSS) method[18] (R package “gamlss”) was used for the generation of smoothed percentile curves for the aforementioned aging metrics. The GAMLSS method was a well-acknowledged model widely applied in medicine (e.g., growth curve fitting), environmental studies, and the financial community. Specifically, GAMLSS is a distributional regression model where all parameters for the assumed response distribution can be modeled as functions for explanatory variables. The models are appropriate when the focus is not solely on the mean (or location) for the distribution but possibly another part of the distribution such as variance, quantiles, skewness, and kurtosis or tails. Since all the parameters for the distribution of the response can be modeled as a function for the explanatory variables, the distribution for the response changes with explanatory variables. The models are extensions for the LMS model that summarizes the data in terms of three smooth age-specific curves, namely, L (lambda), M (mu), and S (sigma). This method does not assume that the outcome follows a normal distribution but instead employs a more flexible distribution that can accommodate skewed or kurtotic data. Parameters corresponding to centrality, dispersion, skewness, and kurtosis are first estimated using maximum likelihood methods for each time point, and the trends for each parameter are smoothed using non-parametric regression in turn. Percentiles are then found by inverting the distribution function. Several tests were performed to examine the fitting degree and applicability of the model, including the Akaike Information Criterion (AIC), against fitted values, density estimates, the normal Q-Q plot, the percentile curves, etc. (**Fig. S1e**). Utilizing the best-fitted model, we generated sex-, income-, and education-specific percentile curves for each aging metric and estimated their age-specific normative values for the 1st, 5th, 25th, 50th, 75th, 95th, and 99th percentiles (**Fig. S1e**). Notably, several chronic diseases were excluded during the analysis of several aging metrics to avoid potential bias. For instance, participants with memory dysfunctions and arthritis were excluded from the analysis for cognitive function and grip strength, respectively. All statistical analyses were performed using R software (version 4.2.2), and a two-tailed P value < 0.05 was considered statistically significant.

**References**

1. Sudlow C, Gallacher J, Allen N, Beral V, Burton P, Danesh J, Downey P, Elliott P, Green J, Landray M *et al*: **UK biobank: an open access resource for identifying the causes of a wide range of complex diseases of middle and old age**. *PLoS medicine* 2015, **12**(3):e1001779.

2. **National Health and Nutrition Examination Surveys** [<https://www.cdc.gov/nchs/nhanes/index.htm>]

3. **China Health and Retirement Longitudinal Study** [<http://charls.pku.edu.cn/>]

4. Li J, Cacchione PZ, Hodgson N, Riegel B, Keenan BT, Scharf MT, Richards KC, Gooneratne NS: **Afternoon Napping and Cognition in Chinese Older Adults: Findings from the China Health and Retirement Longitudinal Study Baseline Assessment**. *Journal of the American Geriatrics Society* 2017, **65**(2):373-380.

5. Prince M, Acosta D, Chiu H, Scazufca M, Varghese M: **Dementia diagnosis in developing countries: a cross-cultural validation study**. *Lancet (London, England)* 2003, **361**(9361):909-917.

6. Duchowny KA, Ackley SF, Brenowitz WD, Wang J, Zimmerman SC, Caunca MR, Glymour MM: **Associations Between Handgrip Strength and Dementia Risk, Cognition, and Neuroimaging Outcomes in the UK Biobank Cohort Study**. *JAMA network open* 2022, **5**(6):e2218314.

7. Chen H, Mui AC: **Factorial validity of the Center for Epidemiologic Studies Depression Scale short form in older population in China**. *International psychogeriatrics* 2014, **26**(1):49-57.

8. Kroenke K, Spitzer RL, Williams JB: **The PHQ-9: validity of a brief depression severity measure**. *Journal of general internal medicine* 2001, **16**(9):606-613.

9. Fragoso CA, Gahbauer EA, Van Ness PH, Concato J, Gill TM: **Peak expiratory flow as a predictor of subsequent disability and death in community-living older persons**. *Journal of the American Geriatrics Society* 2008, **56**(6):1014-1020.

10. **National Health and Nutrition Examination Survey: Spirometry-Pre and Post-Bronchodilator** [<https://wwwn.cdc.gov/Nchs/Nhanes/2011-2012/SPX_G.htm>]

11. **National Health and Nutrition Examination Survey: Dual-Energy X-ray Absorptiometry-Whole Body** [<https://wwwn.cdc.gov/Nchs/Nhanes/2011-2012/DXX_G.htm>]

12. **CHARLS: Blood user guide** [<https://charls.charlsdata.com/Public/ashelf/public/uploads/document/2011-charls-wave1/application/blood_user_guide_en_20140429.pdf>]

13. Mitnitski A, Collerton J, Martin-Ruiz C, Jagger C, von Zglinicki T, Rockwood K, Kirkwood TB: **Age-related frailty and its association with biological markers of ageing**. *BMC medicine* 2015, **13**:161.

14. Blodgett JM, Theou O, Howlett SE, Rockwood K: **A frailty index from common clinical and laboratory tests predicts increased risk of death across the life course**. *GeroScience* 2017, **39**(4):447-455.

15. Feng Z, Cramm JM, Jin C, Twisk J, Nieboer AP: **The longitudinal relationship between income and social participation among Chinese older people**. *SSM - population health* 2020, **11**:100636.

16. **National Health and Nutrition Examination Survey: Demographic Variables and Sample Weights** [<https://wwwn.cdc.gov/Nchs/Nhanes/2015-2016/DEMO_I.htm>]

17. Lyall LM, Wyse CA, Graham N, Ferguson A, Lyall DM, Cullen B, Celis Morales CA, Biello SM, Mackay D, Ward J *et al*: **Association of disrupted circadian rhythmicity with mood disorders, subjective wellbeing, and cognitive function: a cross-sectional study of 91 105 participants from the UK Biobank**. *The lancet Psychiatry* 2018, **5**(6):507-514.

18. **Generalized Additive Models for Location, Scale and Shape** [<https://www.gamlss.com/>]
