# Supplementary material for: Delineating Life‐Course Percentile Curves and Normative Values of Multi‐Systemic Ageing Metrics in the United Kingdom, the United States, and China
Source: J Cachexia Sarcopenia Muscle. 2025 Jun 13;16(3):e13862. doi: 10.1002/jcsm.13862 (PMC12163542; doi:10.1002/jcsm.13862)
Supplement: Supplementary file 2 — Table S1. Basic characteristics of the excluded and included participants in CHARLS. Note: Basic characteristics were expressed as mean±standard deviation and number with percentage for continuous and categorical variables, respectively. CHARLS, the China Health and Retirement Longitudinal Study. Table S2. Basic characteristics of the excluded and included participants in NHANES.Note: Basic characteristics were expressed as mean±standard deviation and number with percentage for continuous and categorical variables, respectively. NHANES, the National Health and Nutrition Examination Survey. Table S3. Basic characteristics of the excluded and included participants in UKB.Note: Basic characteristics were expressed as mean±standard deviation and number with percentage for continuous and categorical variables, respectively. UKB, the UK Biobank. Table S4. Multi‐systemic ageing metrics available from the three datasets. Note: FEV1, forced expiratory volume in the first second; “√” represent the variables are available in this dataset; CHARLS, the China Health and Retirement Longitudinal Study; NHANES, the National Health and Nutrition Examination Survey; UKB, the UK Biobank. Table S5. Biomarkers utilized for the construction of frailty index_Lab and the corresponding cut‐off values in CHARLS and NHANES, respectively. Note: CHARLS, the China Health and Retirement Longitudinal Study; NHANES, the National Health and Nutrition Examination Survey. Table S6. Basic characteristics for participants in total and by different sociodemographic contexts in three datasets. Note: Basic characteristics were expressed as median with the inter‐quartile ranges. The Mann–Whitney U‐test and Cohens'd statistics were used for the comparison for the participants’ characteristics across different subgroups; FEV1, forced expiratory volume in the first second; CHARLS, the China Health and Retirement Longitudinal Study; NHANES, the National Health and Nutrition Examination Survey; UKB, the UK Bioban [file JCSM-16-e13862-s003.doc]

**Table S1 Basic characteristics of the excluded and included participants in CHARLS**

**Note:** Basic characteristics were expressed as mean±standard deviation and number with percentage for continuous and categorical variables, respectively. CHARLS, the China Health and Retirement Longitudinal Study.

|  | Overall (N=21789) | Excluded participants (N=6457) | Included participants (N=15332) | **P** |
| --- | --- | --- | --- | --- |
| **Age, year** | 59.12±10.79 | 55.31±11.96 | 60.54±9.95 | <0.001 |
| **Female, n (%)** | 11304 (52.0) | 3278 (51.2) | 8026 (52.3) | 0.122 |
| **Educational attainment, n (%)** |  |  |  | <0.001 |
| Less than secondary school | 15311 (70.6) | 4407 (69.1) | 10904 (71.2) |  |
| Middle to senior high school | 5962 (27.5) | 1761 (27.6) | 4201 (27.4) |  |
| College and above | 424 (2.0) | 212 (3.3) | 212 (1.4) |  |
| **Moderate physical activity for at least 10 minutes every week, n (%)** | 5501 (55.9) | 1305 (55.4) | 4196 (56.1) | 0.559 |
| **Drinker, n (%)** | 9676 (46.3) | 2577 (46.0) | 7099 (46.4) | 0.613 |
| **Smoking status, n (%)** |  |  |  | 0.001 |
| Never | 11896 (56.9) | 3279 (58.5) | 8617 (56.3) |  |
| Quit | 3168 (15.1) | 770 (13.7) | 2398 (15.7) |  |
| Current | 5856 (28.0) | 1558 (27.8) | 4298 (28.1) |  |

**Table S2 Basic characteristics of the excluded and included participants in NHANES**

|  | **Overall (N=45462)** | **Excluded participants (N=8117)** | **Included participants (N=37345)** | **P** |
| --- | --- | --- | --- | --- |
| **Age, year** | 32.37±24.82 | 30.85±30.99 | 32.69±23.26 | <0.001 |
| **Female, n (%)** | 22990 (50.6) | 4233 (52.1) | 18757 (50.2) | 0.002 |
| **Race, n (%)** |  |  |  | <0.001 |
| Mexican American | 6996 (15.4) | 1102 (13.6) | 5894 (15.8) |  |
| Other Hispanic | 4888 (10.8) | 842 (10.4) | 4046 (10.8) |  |
| Non-Hispanic White | 14984 (33.0) | 3409 (42.0) | 11575 (31.0) |  |
| Non-Hispanic Black | 11177 (24.6) | 1671 (20.6) | 9506 (25.5) |  |
| Other race | 7417 (16.3) | 1093 (13.5) | 6324 (16.9) |  |
| **Educational attainment, n (%)** |  |  |  | 0.006 |
| Less than 9th grade | 2412 (9.2) | 338 (8.3) | 2074 (9.3) |  |
| 9-11th grade | 3290 (12.5) | 482 (11.9) | 2808 (12.7) |  |
| High school gradudate/GED or equivalent | 5933 (22.6) | 878 (21.6) | 5055 (22.8) |  |
| Some college or AA degree | 8094 (30.8) | 1287 (31.7) | 6807 (30.7) |  |
| College graduate or above | 6519 (24.8) | 1079 (26.6) | 5440 (24.5) |  |
| **Ratio of family income to poverty** | 2.30±1.62 | 2.37±1.66 | 2.28±1.62 | <0.001 |
| **Recreational activity, n (%)** |  |  |  | 0.001 |
| Others | 11896 (56.9) | 3279 (58.5) | 8617 (56.3) |  |
| Moderate | 3168 (15.1) | 770 (13.7) | 2398 (15.7) |  |
| Vigorous | 5856 (28.0) | 1558 (27.8) | 4298 (28.1) |  |
| **Non-smokers, n (%)** | 16074 (58.8) | 2192 (53.1) | 13882 (59.8) | <0.001 |
| **Non-drinkers, n (%)** | 5615 (23.4) | 568 (22.5) | 5047 (23.5) | 0.279 |

**Note:** Basic characteristics were expressed as mean±standard deviation and number with percentage for continuous and categorical variables, respectively. NHANES, the National Health and Nutrition Examination Survey.

**Table S3 Basic characteristics of the excluded and included participants in UKB**

|  | **Overall (N=80791)** | **Excluded participants (N=37090)** | **Included participants (N=43701)** | **P** |
| --- | --- | --- | --- | --- |
| **Age, year** | 66.4±8.0 | 68.8±7.6 | 64.4±7.7 | <0.001 |
| **Female, n (%)** | 41704 (51.6) | 19243 (51.9) | 22461 (51.4) | 0.173 |
| **Townsend deprivation index** | -1.9±2.8 | -1.9±2.8 | -1.9±2.7 | 0.639 |
| **White race, n (%)** | 77919 (96.7) | 35697 (96.6) | 42222 (96.8) | 0.087 |
| **Educational attainment, n (%)** |  |  |  | <0.001 |
| Less than high school diploma | 16658 (20.7) | 7885 (21.5) | 8773 (20.1) |  |
| High school graduate or equivalent | 4762 (5.9) | 2210 (6.0) | 2552 (5.8) |  |
| College or above | 58933 (73.3) | 26588 (72.5) | 32345 (74.1) |  |
| **Smoking status, n (%)** |  |  |  | <0.001 |
| Never | 30105 (62.1) | 3150 (60.1) | 26955 (62.3) |  |
| Quit | 16674 (34.4) | 1955 (37.3) | 14719 (34.0) |  |
| Current | 1699 (3.5) | 140 (2.7) | 1559 (3.6) |  |
| **Drinking status, n (%)** |  |  |  | 0.014 |
| Never | 1613 (3.3) | 166 (3.2) | 1447 (3.3) |  |
| Quit | 1649 (3.4) | 214 (4.1) | 1435 (3.3) |  |
| Current | 45354 (93.3) | 4889 (92.8) | 40465 (93.4) |  |

**Note:** Basic characteristics were expressed as mean±standard deviation and number with percentage for continuous and categorical variables, respectively. UKB, the UK Biobank.

**Table S4 Multi-systemic aging metrics available from the three datasets**

|  | **CHARLS (the 2015 wave)** | **NHANES (2011-2020 waves)** | **UKB (the 2014 wave)** |
| --- | --- | --- | --- |
| **Cognitive function** | √ | √ | √ |
| **Depression** | √ | √ |  |
| **Systolic blood pressure** | √ | √ | √ |
| **Diastolic blood pressure** | √ | √ | √ |
| **Pulse** | √ | √ | √ |
| **Body mass index** | √ | √ | √ |
| **Waist circumference** | √ | √ | √ |
| **Grip strength** | √ | √ | √ |
| **Peak expiratory flow** | √ | √ | √ |
| **FEV_1_** |  | √ | √ |
| **Fat-free mass index** |  | √ | √ |
| **Bone mineral content** |  | √ | √ |
| **Bone mineral density** |  | √ | √ |
| **Frailty index_Lab** | √ | √ |  |

**Note:** FEV_1_, forced expiratory volume in the first second; “√” represent the variables are available in this dataset; CHARLS, the China Health and Retirement Longitudinal Study; NHANES, the National Health and Nutrition Examination Survey; UKB, the UK Biobank

**Table S5 Biomarkers utilized for the construction of frailty index_Lab and the corresponding cut-off values in CHARLS and NHANES, respectively**

| **Biomarker** | **CHARLS** | **NHANES** |
| --- | --- | --- |
| Blood urea nitrogen | >20 mg/dL | <2.9 or >8.2 mmol/L |
| C-reactive protein | >3 mg/L | >1 mg/dL |
| Creatinine | >1.4 mg/dL | Male: <60 or >110 umol/L; Female: <45 or >90 umol/L |
| HDL-Cholesterol | <40 mg/dL | ≥1.3 mmol/L |
| Glucose | ≥126 mg/dL | <3.9 or >6.1 mmol/L |
| Glycohemoglobin | ≥6.5% | >5.7% |
| Hemoglobin | Male: <13 g/dL; Female: <12 g/dL | Male:<13.5 or >18 g/dL; Female:<12 or >16 g/dL |
| Platelet count SI | <170*10^9^/L | <150 or >450*1000 cells/uL |
| Total cholesterol | ≥240 mg/dL | <3.88 or >6.47 mmol/L |
| Triglyceride | ≥200 mg/dL | <0.11 or >2.74 mmol/L |
| Uric acid | Male: <240 or >510 umol/L; Female: <160 or >430 umol/L | Male: <240 or >510 umol/L; Female: <160 or >430umol/L |
| White blood cell | > 7*10^9^/L | > 7*10^9^/L |

**Note:** CHARLS, the China Health and Retirement Longitudinal Study; NHANES, the National Health and Nutrition Examination Survey.

**Table S6 Basic characteristics for participants in total and by different sociodemographic contexts in three datasets**

| **CHARLS** | | | | | | | | | | | | | |
| --- | --- | --- | --- | --- | --- | --- | --- | --- | --- | --- | --- | --- | --- |
|  | **Overall**  **(N=15332)** | **Male**  **(N=7306)** | **Female**  **(N=8026)** | **P** | **Cohen’s d** | **Low-income**  **(N=614)** | **High-income**  **(N=2398)** | **P** | **Cohen’s d** | **Low-education**  **(N=10904)** | **High-education**  **(N=4413)** | **P** | **Cohen’s d** |
| **Age, year** | 60.00  (52.00, 67.00) | 60.50  (52.00, 68.00) | 60.00  (52.00, 66.00) | <0.001 | 0.08 | 62.00  (53.00, 68.00) | 53.00  (49.00, 59.00) | <0.001 | 0.97 | 62.00  (53.00, 69.00) | 56.00  (51.00, 62.00) | <0.001 | 0.42 |
| **Cognition function score** | 10.50  (7.00, 14.00) | 11.50  (8.50, 14.00) | 9.50  (5.50, 13.00) | <0.001 | 0.41 | 10.00  (7.00, 13.50) | 13.00  (10.50, 15.00) | <0.001 | -0.78 | 9.50  (6.00, 12.50) | 13.50  (11.00, 15.50) | <0.001 | -1.00 |
| **Depression score** | 6.00  (3.00, 12.00) | 6.00  (3.00, 10.00) | 8.00  (4.00, 13.00) | <0.001 | -0.35 | 7.00  (3.00, 12.00) | 5.00  (2.00, 8.00) | <0.001 | 0.50 | 7.00  (3.00, 12.00) | 5.00  (2.00, 9.00) | <0.001 | 0.37 |
| **Systolic blood pressure, mmHg** | 126.67  (114.33, 141.00) | 127.67  (115.67, 141.67) | 125.33  (113.00, 140.67) | <0.001 | 0.09 | 127.67  (115.08, 143.33) | 124.00 (113.33, 136.67) | <0.001 | 0.17 | 127.00 (114.67, 142.00) | 125.67 (113.67, 139.33) | <0.001 | 0.09 |
| **Diastolic blood pressure, mmHg** | 74.67  (67.33, 82.67) | 76.00  (68.67, 84.00) | 73.67  (66.67, 81.33) | <0.001 | 0.21 | 74.00  (67.33, 82.33) | 75.67  (68.00, 83.67) | 0.003 | -0.15 | 74.33  (67.00, 82.00) | 75.67  (68.33, 84.00) | <0.001 | -0.13 |
| **Pulse** | 73.33  (66.67, 80.67) | 73.00  (66.00, 80.67) | 73.83  (67.33, 80.67) | <0.001 | -0.06 | 72.00  (66.33, 79.92) | 73.00  (66.67, 80.00) | 0.128 | -0.05 | 73.67  (66.67, 80.67) | 73.00  (66.42, 80.33) | 0.019 | 0.04 |
| **Body mass index, kg/m^2^** | 23.57  (21.21, 26.17) | 23.15  (20.81, 25.69) | 24.00  (21.65, 26.56) | <0.001 | -0.21 | 23.02  (20.68, 25.50) | 23.99  (21.78, 26.33) | <0.001 | -0.24 | 23.35  (20.96, 25.98) | 24.15  (21.83, 26.48) | <0.001 | -0.17 |
| **Waist circumference, cm** | 86.00  (78.90, 93.40) | 85.80  (78.50, 93.60) | 86.40  (79.00, 93.20) | 0.247 | -0.01 | 84.70  (77.95, 92.00) | 87.00  (80.00, 94.00) | <0.001 | -0.20 | 85.40  (78.20, 92.80) | 87.80  (80.50, 94.80) | <0.001 | -0.21 |
| **Grip strength, kg** | 29.80  (23.00, 37.50) | 37.00  (31.00, 43.00) | 24.60  (20.00, 29.00) | <0.001 | 1.53 | 29.70  (23.00, 37.00) | 37.00  (29.50, 43.70) | <0.001 | -0.68 | 28.00  (22.00, 35.00) | 34.00  (27.50, 41.30) | <0.001 | -0.58 |
| **Peak expiratory flow, ml/s** | 4722.03  (3444.31, 6055.31) | 5721.99  (4222.05, 7166.38) | 4055.39  (3055.43, 4999.80) | <0.001 | 0.93 | 4555.37  (3333.20, 5777.55) | 5777.55  (4388.71, 7221.93) | <0.001 | -0.56 | 4388.71  (3222.09, 5666.44) | 5499.78  (4110.95, 6888.61) | <0.001 | -0.54 |
| **Frailty index_Lab** | 0.17  (0.08, 0.25) | 0.17  (0.08, 0.25) | 0.08  (0.08, 0.17) | <0.001 | 0.20 | 0.17  (0.08, 0.25) | 0.17  (0.08, 0.25) | 0.51 | 0.01 | 0.17  (0.08, 0.25) | 0.17  (0.08, 0.25) | <0.001 | 0.07 |
| **NHANES** | | | | | | | | | | | | | |
|  | **Overall**  **(N=37345)** | **Male**  **(N=18588)** | **Female**  **(N=18757)** | **P** | **Cohen’s d** | **Low-income**  **(N=9237)** | **High-income**  **(N=24359)** | **P** | **Cohen’s d** | **Low-education**  **(N=2074)** | **High-education**  **(N=20110)** | **P** | **Cohen’s d** |
| **Age, year** | 29.00  (11.00, 52.00) | 28.00  (11.00, 52.00) | 30.00  (12.00, 53.00) | <0.001 | -0.04 | 19.00  (9.00, 45.00) | 32.00  (13.00, 54.00) | <0.001 | -0.29 | 59.00  (44.00, 69.00) | 47.00  (33.00, 61.00) | <0.001 | 0.56 |
| **Cognition function score** | 16.00  (12.00, 20.00) | 16.00  (12.00, 20.00) | 16.00  (12.00, 19.00) | 0.144 | 0.06 | 14.50  (11.00, 18.00) | 16.00  (13.00, 20.00) | <0.001 | -0.36 | 13.00  (10.50, 16.00) | 16.00  (13.00, 20.00) | <0.001 | -0.58 |
| **Depression score** | 2.00  (0.00, 4.00) | 1.00  (0.00, 4.00) | 2.00  (0.00, 5.00) | <0.001 | -0.23 | 3.00  (0.00, 6.00) | 1.00  (0.00, 4.00) | <0.001 | 0.33 | 2.00  (0.00, 6.00) | 2.00  (0.00, 4.00) | 0.294 | 0.17 |
| **Systolic blood pressure, mmHg** | 115.67  (106.00, 128.00) | 118.00  (108.67, 129.33) | 112.67  (103.67, 126.00) | <0.001 | 0.20 | 114.00  (104.67, 126.67) | 116.00  (106.00, 128.00) | <0.001 | -0.07 | 126.67  (115.33, 140.67) | 120.67  (111.00, 132.67) | <0.001 | 0.34 |
| **Diastolic blood pressure, mmHg** | 68.00  (60.00, 76.67) | 69.33  (60.00, 77.33) | 67.33  (60.00, 75.33) | <0.001 | 0.10 | 66.67  (58.00, 75.00) | 68.67  (60.67, 76.67) | <0.001 | -0.16 | 70.33  (63.33, 78.00) | 72.00  (64.67, 79.33) | <0.001 | -0.16 |
| **Pulse** | 72.67  (65.00, 82.00) | 71.67  (64.00, 80.00) | 74.00  (67.00, 82.00) | <0.001 | -0.24 | 74.00  (66.00, 82.33) | 72.00  (64.33, 81.00) | <0.001 | 0.14 | 70.00  (62.00, 78.00) | 70.67  (64.00, 79.00) | <0.001 | -0.09 |
| **Body mass index, kg/m^2^** | 24.80  (19.50, 30.40) | 24.80  (19.40, 29.60) | 24.90  (19.70, 31.20) | <0.001 | -0.12 | 23.70  (18.20, 29.80) | 25.20  (20.00, 30.50) | <0.001 | -0.12 | 28.50  (25.10, 32.50) | 28.20  (24.30, 33.10) | 0.115 | -0.02 |
| **Waist circumference, cm** | 87.50  (70.40, 102.70) | 89.10  (70.30, 103.60) | 86.20  (70.50, 101.60) | <0.001 | 0.05 | 83.00  (63.92, 100.80) | 88.80  (72.20, 103.30) | <0.001 | -0.19 | 99.25  (90.40, 108.03) | 97.80  (87.00, 109.60) | 0.004 | 0.03 |
| **Grip strength, kg** | 31.60  (23.60, 42.30) | 41.60  (29.60, 49.30) | 27.50  (21.90, 31.90) | <0.001 | 1.05 | 29.80  (21.30, 39.90) | 32.20  (24.40, 43.10) | <0.001 | -0.2 | 32.40  (25.02, 41.20) | 35.90  (28.90, 46.20) | <0.001 | -0.40 |
| **Peak expiratory flow, ml/s** | 7180.50  (5403.75, 9005.00) | 8665.50  (6207.75, 10286.75) | 6431.50  (5084.50, 7520.00) | <0.001 | 0.83 | 6775.00  (4842.75, 8636.25) | 7392.00  (5693.50, 9182.50) | <0.001 | -0.25 | 7091.00  (5415.00, 8644.00) | 8005.00  (6586.00, 9703.50) | <0.001 | -0.42 |
| **FEV_1_, ml** | 2852.00  (2154.00, 3549.00) | 3380.00  (2535.00, 4030.00) | 2519.50  (2000.00, 2996.25) | <0.001 | 0.89 | 2781.00  (2063.00, 3482.00) | 2893.50  (2205.00, 3578.50) | <0.001 | -0.12 | 2674.50  (2110.75, 3321.75) | 3039.00  (2440.00, 3692.00) | <0.001 | -0.39 |
| **Fat-free mass index, kg/m^2^** | 17.33  (14.72, 20.16) | 18.78  (16.05, 21.17) | 16.00  (14.05, 18.39) | <0.001 | 0.63 | 17.04  (14.43, 19.87) | 17.41  (14.81, 20.26) | <0.001 | -0.09 | 19.44  (17.37, 21.31) | 18.85  (16.54, 21.20) | 0.003 | 0.09 |
| **Bone mineral content, kg** | 2.11  (1.70, 2.49) | 2.36  (1.87, 2.71) | 1.94  (1.63, 2.22) | <0.001 | 0.61 | 2.03  (1.57, 2.41) | 2.14  (1.74, 2.52) | <0.001 | -0.2 | 2.15  (1.87, 2.44) | 2.32  (2.02, 2.64) | <0.001 | -0.41 |
| **Bone mineral density, g/cm^2^** | 1.06  (0.95, 1.15) | 1.09  (0.96, 1.18) | 1.04  (0.94, 1.12) | <0.001 | 0.29 | 1.05  (0.92, 1.13) | 1.07  (0.96, 1.15) | <0.001 | -0.16 | 1.08  (1.01, 1.15) | 1.11  (1.04, 1.18) | <0.001 | -0.33 |
| **Frailty index_Lab** | 0.17  (0.08, 0.25) | 0.17  (0.08, 0.33) | 0.17  (0.08, 0.25) | <0.001 | 0.23 | 0.17  (0.08, 0.33) | 0.17  (0.08, 0.25) | <0.001 | 0.19 | 0.25  (0.17, 0.33) | 0.17  (0.08, 0.25) | <0.001 | 0.35 |
| **UKB** | | | | | | | | | | | | | |
|  | **Overall**  **(N=43701)** | **Male**  **(N=21240)** | **Female**  **(N=22461)** | **P** | **Cohen’s d** | **Low-income**  **(N=9680)** | **High-income**  **(N=33977)** | **P** | **Cohen’s d** | **Low-education**  **(N=9445)** | **High--education**  **(N=34225)** | **P** | **Cohen’s d** |
| **Age, year** | 64.80  (58.32, 70.38) | 65.75  (59.00, 71.10) | 63.99  (57.76, 69.62) | <0.001 | 0.16 | 63.33  (56.85, 69.53) | 65.21  (58.76, 70.57) | <0.001 | -0.17 | 67.28  (61.02, 71.91) | 64.08  (57.71, 69.81) | <0.001 | 0.32 |
| **Cognition function score** | 7.00  (5.00, 8.00) | 7.00  (5.00, 8.00) | 6.00  (5.00, 8.00) | <0.001 | 0.12 | 6.00  (5.00, 8.00) | 7.00  (5.00, 8.00) | <0.001 | -0.11 | 5.00  (4.00, 7.00) | 7.00  (5.00, 8.00) | <0.001 | -0.75 |
| **Systolic blood pressure, mmHg** | 137.50  (125.50, 150.50) | 140.50  (130.00, 152.50) | 134.00  (122.00, 148.00) | <0.001 | 0.33 | 136.00  (124.00, 149.50) | 138.00  (126.00, 151.00) | <0.001 | -0.08 | 140.50  (129.00, 153.50) | 136.50  (125.00, 149.50) | <0.001 | 0.20 |
| **Diastolic blood pressure, mmHg** | 78.50  (72.00, 85.50) | 80.50  (74.00, 87.00) | 76.50  (70.00, 83.50) | <0.001 | 0.36 | 78.50  (72.00, 85.50) | 78.50  (72.00, 85.50) | 0.545 | -0.01 | 79.00  (72.50, 86.00) | 78.50  (71.50, 85.00) | <0.001 | 0.07 |
| **Pulse** | 69.00  (61.00, 77.00) | 67.00  (59.00, 75.00) | 71.00  (64.00, 79.00) | <0.001 | -0.31 | 69.00  (62.00, 78.00) | 69.00  (61.00, 77.00) | <0.001 | 0.05 | 70.00  (62.00, 78.00) | 69.00  (61.00, 77.00) | <0.001 | 0.09 |
| **Body mass index, kg/m^2^** | 25.96  (23.52, 28.90) | 26.54  (24.35, 29.17) | 25.29  (22.76, 28.56) | <0.001 | 0.21 | 26.33  (23.64, 29.55) | 25.85  (23.49, 28.73) | <0.001 | 0.13 | 26.80  (24.34, 29.82) | 25.72  (23.32, 28.63) | <0.001 | 0.23 |
| **Waist circumference, cm** | 88.00  (79.00, 97.00) | 93.00  (87.00, 101.00) | 81.00  (74.00, 90.00) | <0.001 | 1.00 | 89.00  (80.00, 98.00) | 88.00  (79.00, 96.00) | <0.001 | 0.09 | 91.00  (82.00, 99.00) | 87.00  (79.00, 96.00) | <0.001 | 0.24 |
| **Grip strength, kg** | 30.00  (24.00, 40.00) | 40.00  (34.00, 45.00) | 24.00  (20.00, 28.00) | <0.001 | 2.08 | 30.00  (24.00, 40.00) | 30.00  (24.00, 40.00) | 0.018 | -0.03 | 30.00  (22.00, 40.00) | 30.00  (24.00, 40.00) | <0.001 | -0.07 |
| **Peak expiratory flow, ml/s** | 5574.78  (4222.05, 7141.38) | 6988.61  (5344.23, 8366.33) | 4810.92  (3710.96, 5766.44) | <0.001 | 1.10 | 5483.11  (4160.94, 6994.16) | 5599.78  (4238.72, 7183.05) | <0.001 | -0.05 | 5308.12  (3938.73, 6921.95) | 5638.66  (4305.38, 7208.04) | <0.001 | -0.14 |
| **FEV_1_, ml** | 2540.00  (2083.33, 3103.33) | 3080.00  (2610.00, 3525.00) | 2200.00  (1870.00, 2525.00) | <0.001 | 1.42 | 2495.00  (2046.67, 3046.67) | 2550.00  (2090.00, 3116.67) | <0.001 | -0.06 | 2435.00  (1983.33, 2983.33) | 2563.33  (2110.00, 3136.67) | <0.001 | -0.17 |
| **Fat-free mass index, kg/m^2^** | 17.17  (15.57, 18.86) | 18.76  (17.70, 19.94) | 15.70  (14.81, 16.78) | <0.001 | 1.83 | 17.23  (15.74, 18.84) | 17.17  (15.54, 18.87) | 0.248 | 0.04 | 17.36  (15.74, 19.06) | 17.12  (15.53, 18.80) | 0.006 | 0.09 |
| **Bone mineral content, kg** | 2.58  (2.16, 3.07) | 3.07  (2.80, 3.35) | 2.18  (1.97, 2.41) | <0.001 | 2.38 | 2.57  (2.17, 3.03) | 2.59  (2.16, 3.08) | 0.083 | -0.02 | 2.60  (2.15, 3.09) | 2.58  (2.16, 3.06) | 0.569 | 0.01 |
| **Bone mineral density, g/cm^2^** | 1.20  (1.09, 1.31) | 1.29  (1.21, 1.37) | 1.11  (1.03, 1.20) | <0.001 | 1.47 | 1.20  (1.10, 1.30) | 1.20  (1.09, 1.31) | 0.584 | 0.01 | 1.20  (1.09, 1.31) | 1.20  (1.09, 1.31) | 0.115 | -0.02 |

**Note:** Basic characteristics were expressed as median with the inter-quartile ranges. The **Mann-Whitney U-test and Cohens’d statistics** were used for the comparison for the participants’ characteristics across different subgroups; FEV1, forced expiratory volume in the first second; CHARLS, the China Health and Retirement Longitudinal Study; NHANES, the National Health and Nutrition Examination Survey; UKB, the UK Biobank.

**Table S7** **Age-specific percentile values of** **multi-systemic aging metrics for males in three datasets**

|  |  | **CHARLS** | | | | | | | **NHANES** | | | | | | | **UKB** | | | | | | |
| --- | --- | --- | --- | --- | --- | --- | --- | --- | --- | --- | --- | --- | --- | --- | --- | --- | --- | --- | --- | --- | --- | --- |
|  | **Age,**  **years** | **1st** | **5th** | **25th** | **50th** | **75th** | **95th** | **99th** | **1st** | **5th** | **25th** | **50th** | **75th** | **95th** | **99th** | **1st** | **5th** | **25th** | **50th** | **75th** | **95th** | **99th** |
| **Cognitive function score** | **50** | 3.9 | 6.8 | 10.5 | 12.7 | 14.8 | 17.6 | 19.4 |  |  |  |  |  |  |  | 2.0 | 3.4 | 5.4 | 6.9 | 8.4 | 10.6 | 12.2 |
|  | **60** | 2.4 | 5.1 | 9.1 | 11.6 | 13.8 | 16.9 | 18.9 | 5.9 | 8.7 | 13.2 | 16.8 | 20.7 | 27.0 | 32.0 | 2.2 | 3.5 | 5.4 | 6.8 | 8.3 | 10.4 | 11.9 |
|  | **70** | 1.3 | 3.4 | 7.5 | 10.3 | 12.9 | 16.3 | 18.6 | 5.8 | 8.5 | 12.9 | 16.3 | 20.0 | 26.0 | 30.8 | 2.0 | 3.3 | 5.2 | 6.6 | 8.1 | 10.2 | 11.7 |
|  | **80** | 0.7 | 2.1 | 5.5 | 8.3 | 11.0 | 14.6 | 17.0 | 5.0 | 7.3 | 10.9 | 13.7 | 16.8 | 21.8 | 25.8 | 1.5 | 2.7 | 4.6 | 5.9 | 7.3 | 9.4 | 10.8 |
|  | **90** | 0.5 | 1.3 | 3.9 | 6.1 | 8.5 | 11.7 | 13.8 |  |  |  |  |  |  |  |  |  |  |  |  |  |  |
|  | **100** | 0.2 | 0.5 | 1.6 | 2.5 | 3.6 | 5.0 | 6.0 |  |  |  |  |  |  |  |  |  |  |  |  |  |  |
| **Depression score** | **20** |  |  |  |  |  |  |  | 0 | 0.1 | 0.8 | 1.9 | 3.9 | 8.4 | 12.9 |  |  |  |  |  |  |  |
|  | **30** |  |  |  |  |  |  |  | 0 | 0.1 | 0.8 | 1.9 | 3.8 | 8.1 | 12.5 |  |  |  |  |  |  |  |
|  | **40** |  |  |  |  |  |  |  | 0 | 0.1 | 0.8 | 1.8 | 3.7 | 8.0 | 12.3 |  |  |  |  |  |  |  |
|  | **50** | 0 | 0.0 | 0.4 | 2.8 | 8.2 | 17.4 | 23.1 | 0 | 0.1 | 0.8 | 1.9 | 3.8 | 8.3 | 12.8 |  |  |  |  |  |  |  |
|  | **60** | 0 | 0.0 | 0.6 | 3.4 | 9.3 | 19.0 | 24.9 | 0 | 0.1 | 0.8 | 2.0 | 4.0 | 8.7 | 13.3 |  |  |  |  |  |  |  |
|  | **70** | 0 | 0.0 | 0.9 | 4.1 | 10.3 | 20.0 | 25.9 | 0 | 0.1 | 0.8 | 1.8 | 3.6 | 7.9 | 12.1 |  |  |  |  |  |  |  |
|  | **80** | 0 | 0.0 | 0.8 | 3.8 | 9.5 | 18.7 | 24.2 | 0 | 0.1 | 0.7 | 1.6 | 3.2 | 7.0 | 10.8 |  |  |  |  |  |  |  |
|  | **90** | 0 | 0.0 | 0.4 | 2.5 | 7.5 | 15.9 | 21.1 |  |  |  |  |  |  |  |  |  |  |  |  |  |  |
|  | **100** | 0 | 0.0 | 0.1 | 1.4 | 5.5 | 13.4 | 18.2 |  |  |  |  |  |  |  |  |  |  |  |  |  |  |
| **Systolic blood pressure, mmHg** | **20** |  |  |  |  |  |  |  | 94.1 | 100.2 | 108.8 | 115.0 | 121.9 | 133.8 | 144.8 |  |  |  |  |  |  |  |
|  | **30** |  |  |  |  |  |  |  | 96.4 | 103.0 | 112.5 | 119.4 | 127.1 | 140.7 | 153.3 |  |  |  |  |  |  |  |
|  | **40** |  |  |  |  |  |  |  | 95.7 | 103.0 | 113.7 | 121.6 | 130.5 | 146.4 | 161.6 |  |  |  |  |  |  |  |
|  | **50** | 92.9 | 100.8 | 113.7 | 124.0 | 135.8 | 155.9 | 172.8 | 95.2 | 103.4 | 115.4 | 124.5 | 134.9 | 153.9 | 172.5 | 104.4 | 112.4 | 124.2 | 133.0 | 142.5 | 157.9 | 170.6 |
|  | **60** | 93.3 | 101.9 | 116.0 | 127.4 | 140.6 | 163.3 | 182.8 | 95.6 | 104.6 | 117.9 | 128.2 | 140.0 | 162.1 | 184.1 | 106.0 | 114.9 | 128.1 | 138.0 | 148.7 | 166.4 | 181.1 |
|  | **70** | 93.8 | 103.0 | 118.4 | 131.0 | 145.7 | 171.4 | 193.9 | 96.0 | 105.6 | 120.1 | 131.5 | 144.7 | 169.8 | 195.4 | 108.5 | 118.1 | 132.6 | 143.5 | 155.3 | 175.0 | 191.5 |
|  | **80** | 95.4 | 104.9 | 120.8 | 133.9 | 149.1 | 176.0 | 199.5 | 95.6 | 106.0 | 121.8 | 134.4 | 149.2 | 177.8 | 207.9 | 109.9 | 120.4 | 136.4 | 148.5 | 161.8 | 184.0 | 202.8 |
|  | **90** | 98.6 | 107.9 | 123.5 | 136.2 | 150.8 | 176.4 | 198.5 |  |  |  |  |  |  |  |  |  |  |  |  |  |  |
|  | **100** | 102.1 | 111.1 | 125.9 | 137.9 | 151.5 | 174.9 | 194.9 |  |  |  |  |  |  |  |  |  |  |  |  |  |  |
| **Diastolic blood pressure, mmHg** | **20** |  |  |  |  |  |  |  | 39.5 | 48.1 | 58.4 | 65.0 | 71.7 | 82.4 | 91.6 |  |  |  |  |  |  |  |
|  | **30** |  |  |  |  |  |  |  | 46.4 | 55.0 | 65.4 | 72.0 | 78.7 | 89.4 | 98.6 |  |  |  |  |  |  |  |
|  | **40** |  |  |  |  |  |  |  | 50.0 | 58.9 | 69.5 | 76.3 | 83.2 | 94.2 | 103.7 |  |  |  |  |  |  |  |
|  | **50** | 54.0 | 60.3 | 70.1 | 77.6 | 85.8 | 98.9 | 109.2 | 50.7 | 59.7 | 70.5 | 77.4 | 84.3 | 95.5 | 105.1 | 60.1 | 66.0 | 74.8 | 81.2 | 88.0 | 98.2 | 105.9 |
|  | **60** | 54.1 | 60.1 | 69.6 | 76.8 | 84.6 | 97.1 | 107.0 | 47.8 | 57.0 | 68.1 | 75.2 | 82.4 | 93.9 | 103.8 | 61.2 | 66.9 | 75.4 | 81.6 | 88.1 | 97.9 | 105.2 |
|  | **70** | 52.2 | 58.1 | 67.3 | 74.3 | 82.0 | 94.2 | 103.8 | 42.2 | 51.7 | 63.2 | 70.6 | 78.1 | 90.0 | 100.4 | 58.4 | 64.3 | 73.1 | 79.4 | 86.1 | 96.3 | 104.0 |
|  | **80** | 49.4 | 55.1 | 64.2 | 71.1 | 78.7 | 90.8 | 100.3 | 35.9 | 45.6 | 57.4 | 65.0 | 72.6 | 85.0 | 95.6 | 54.8 | 61.1 | 70.5 | 77.4 | 84.7 | 95.8 | 104.2 |
|  | **90** | 47.3 | 52.8 | 61.4 | 68.0 | 75.2 | 86.8 | 95.9 |  |  |  |  |  |  |  |  |  |  |  |  |  |  |
|  | **100** | 44.9 | 50.1 | 58.2 | 64.4 | 71.2 | 82.0 | 90.5 |  |  |  |  |  |  |  |  |  |  |  |  |  |  |
| **Pulse** | **20** |  |  |  |  |  |  |  | 49.4 | 55.0 | 64.0 | 71.3 | 79.5 | 93.6 | 105.8 |  |  |  |  |  |  |  |
|  | **30** |  |  |  |  |  |  |  | 48.4 | 53.9 | 62.7 | 69.8 | 77.9 | 91.8 | 103.8 |  |  |  |  |  |  |  |
|  | **40** |  |  |  |  |  |  |  | 48.5 | 54.0 | 62.9 | 70.1 | 78.2 | 92.2 | 104.3 |  |  |  |  |  |  |  |
|  | **50** | 52.7 | 58.3 | 67.1 | 73.9 | 81.5 | 94.0 | 104.2 | 47.8 | 53.4 | 62.5 | 69.8 | 78.2 | 92.7 | 105.3 | 46.5 | 52.0 | 61.2 | 68.6 | 77.2 | 92.3 | 105.3 |
|  | **60** | 51.3 | 57.0 | 65.8 | 72.8 | 80.4 | 93.2 | 103.7 | 46.9 | 52.5 | 61.6 | 68.9 | 77.3 | 92.0 | 104.7 | 45.0 | 50.5 | 59.6 | 67.1 | 75.8 | 91.0 | 104.3 |
|  | **70** | 50.1 | 55.8 | 64.9 | 72.0 | 79.9 | 93.1 | 104.1 | 46.1 | 51.6 | 60.5 | 67.6 | 75.8 | 90.0 | 102.4 | 44.7 | 50.1 | 59.1 | 66.4 | 74.9 | 89.9 | 102.9 |
|  | **80** | 49.6 | 55.4 | 64.8 | 72.2 | 80.5 | 94.4 | 106.0 | 45.3 | 50.7 | 59.3 | 66.3 | 74.3 | 88.3 | 100.4 | 44.4 | 49.6 | 58.3 | 65.3 | 73.5 | 87.7 | 100.0 |
|  | **90** | 49.0 | 54.9 | 64.2 | 71.6 | 79.8 | 93.7 | 105.2 |  |  |  |  |  |  |  |  |  |  |  |  |  |  |
|  | **100** | 47.8 | 53.6 | 63.0 | 70.4 | 78.7 | 92.7 | 104.4 |  |  |  |  |  |  |  |  |  |  |  |  |  |  |
| **Body mass index, kg/m^2^** | **20** |  |  |  |  |  |  |  | 15.9 | 17.8 | 21.1 | 24.1 | 28.1 | 37.3 | 49.1 |  |  |  |  |  |  |  |
|  | **30** |  |  |  |  |  |  |  | 18.1 | 20.2 | 23.9 | 27.3 | 31.8 | 41.9 | 54.8 |  |  |  |  |  |  |  |
|  | **40** |  |  |  |  |  |  |  | 19.3 | 21.4 | 25.2 | 28.5 | 32.9 | 42.6 | 54.2 |  |  |  |  |  |  |  |
|  | **50** | 17.4 | 19.0 | 21.7 | 23.9 | 26.3 | 30.5 | 33.9 | 19.6 | 21.7 | 25.3 | 28.6 | 32.9 | 42.1 | 53.0 | 19.6 | 21.4 | 24.3 | 26.7 | 29.6 | 35.3 | 41.3 |
|  | **60** | 16.9 | 18.5 | 21.1 | 23.2 | 25.5 | 29.5 | 32.8 | 19.4 | 21.4 | 25.0 | 28.2 | 32.4 | 41.3 | 51.7 | 19.9 | 21.6 | 24.4 | 26.7 | 29.4 | 34.6 | 39.9 |
|  | **70** | 16.1 | 17.7 | 20.3 | 22.4 | 24.8 | 29.0 | 32.5 | 19.2 | 21.2 | 24.6 | 27.6 | 31.5 | 39.7 | 49.0 | 20.0 | 21.7 | 24.4 | 26.6 | 29.1 | 34.1 | 39.0 |
|  | **80** | 15.2 | 16.8 | 19.4 | 21.5 | 23.9 | 28.0 | 31.5 | 19.0 | 20.9 | 24.1 | 26.8 | 30.4 | 37.6 | 45.6 | 19.6 | 21.2 | 23.6 | 25.6 | 27.9 | 32.1 | 36.3 |
|  | **90** | 14.9 | 16.4 | 18.8 | 20.8 | 23.0 | 26.9 | 30.2 |  |  |  |  |  |  |  |  |  |  |  |  |  |  |
|  | **100** | 14.8 | 16.2 | 18.5 | 20.3 | 22.3 | 25.8 | 28.7 |  |  |  |  |  |  |  |  |  |  |  |  |  |  |
| **Waist circumference, cm** | **20** |  |  |  |  |  |  |  | 62.4 | 67.6 | 76.7 | 84.8 | 95.4 | 118.7 | 147.4 |  |  |  |  |  |  |  |
|  | **30** |  |  |  |  |  |  |  | 70.1 | 75.9 | 86.0 | 95.0 | 106.7 | 132.3 | 163.6 |  |  |  |  |  |  |  |
|  | **40** |  |  |  |  |  |  |  | 74.4 | 80.3 | 90.5 | 99.5 | 110.9 | 135.3 | 163.9 |  |  |  |  |  |  |  |
|  | **50** | 52.7 | 67.4 | 80.8 | 87.9 | 94.5 | 105.0 | 114.3 | 76.2 | 82.1 | 92.2 | 101.0 | 112.3 | 135.9 | 162.9 | 70.8 | 76.4 | 84.9 | 91.4 | 98.9 | 112.2 | 124.7 |
|  | **60** | 51.4 | 66.0 | 79.2 | 86.3 | 92.9 | 103.2 | 112.5 | 77.1 | 83.0 | 93.0 | 101.7 | 112.8 | 135.7 | 161.5 | 72.4 | 78.0 | 86.4 | 92.9 | 100.2 | 113.3 | 125.5 |
|  | **70** | 47.7 | 63.3 | 77.4 | 84.9 | 91.9 | 102.7 | 112.4 | 78.1 | 83.9 | 93.6 | 102.0 | 112.5 | 133.9 | 157.3 | 73.9 | 79.5 | 87.8 | 94.1 | 101.3 | 114.0 | 125.9 |
|  | **80** | 45.4 | 61.3 | 75.8 | 83.4 | 90.5 | 101.5 | 111.3 | 79.0 | 84.6 | 93.9 | 101.9 | 111.7 | 131.2 | 152.0 | 73.8 | 79.2 | 87.2 | 93.3 | 100.1 | 112.2 | 123.3 |
|  | **90** | 43.3 | 59.6 | 74.6 | 82.4 | 89.6 | 100.9 | 110.9 |  |  |  |  |  |  |  |  |  |  |  |  |  |  |
|  | **100** | 41.4 | 58.1 | 73.4 | 81.4 | 88.7 | 100.2 | 110.4 |  |  |  |  |  |  |  |  |  |  |  |  |  |  |
| **Grip strength, kg** | **20** |  |  |  |  |  |  |  | 22.8 | 28.5 | 36.7 | 42.7 | 49.1 | 59.4 | 67.8 |  |  |  |  |  |  |  |
|  | **30** |  |  |  |  |  |  |  | 30.3 | 36.2 | 44.7 | 50.8 | 57.2 | 67.5 | 75.8 |  |  |  |  |  |  |  |
|  | **40** |  |  |  |  |  |  |  | 31.9 | 37.3 | 44.9 | 50.3 | 56.0 | 65.0 | 72.3 |  |  |  |  |  |  |  |
|  | **50** | 21.4 | 28.5 | 36.9 | 42.1 | 47.2 | 54.8 | 60.7 | 29.8 | 34.8 | 41.9 | 46.9 | 52.3 | 60.7 | 67.4 | 23.3 | 30.3 | 39.0 | 44.7 | 50.4 | 59.6 | 67.3 |
|  | **60** | 17.7 | 24.7 | 32.8 | 37.9 | 42.8 | 50.1 | 55.9 | 25.6 | 30.6 | 37.7 | 42.8 | 48.2 | 56.8 | 63.8 | 21.8 | 28.3 | 36.4 | 41.7 | 47.1 | 55.6 | 62.8 |
|  | **70** | 13.8 | 20.4 | 28.1 | 32.8 | 37.5 | 44.4 | 49.7 | 21.3 | 26.1 | 33.1 | 38.2 | 43.5 | 52.2 | 59.2 | 19.8 | 25.8 | 33.3 | 38.3 | 43.2 | 51.2 | 57.9 |
|  | **80** | 8.1 | 14.5 | 22.4 | 27.3 | 32.0 | 38.9 | 44.3 | 17.0 | 21.4 | 27.9 | 32.7 | 37.9 | 46.1 | 52.9 | 15.9 | 21.6 | 28.9 | 33.6 | 38.4 | 46.0 | 52.5 |
|  | **90** | 4.4 | 9.7 | 17.3 | 22.1 | 26.7 | 33.4 | 38.7 |  |  |  |  |  |  |  |  |  |  |  |  |  |  |
|  | **100** | 2.3 | 6.2 | 12.8 | 17.2 | 21.4 | 27.6 | 32.4 |  |  |  |  |  |  |  |  |  |  |  |  |  |  |
| **FEV_1_, ml** | **20** |  |  |  |  |  |  |  | 2256.23 | 2722.14 | 3397.54 | 3882.50 | 4387.94 | 5168.53 | 5769.97 |  |  |  |  |  |  |  |
|  | **30** |  |  |  |  |  |  |  | 2637.95 | 3088.00 | 3733.86 | 4194.04 | 4671.20 | 5404.20 | 5966.35 |  |  |  |  |  |  |  |
|  | **40** |  |  |  |  |  |  |  | 2430.83 | 2843.26 | 3435.00 | 3856.52 | 4293.54 | 4964.77 | 5479.48 |  |  |  |  |  |  |  |
|  | **50** |  |  |  |  |  |  |  | 2067.65 | 2454.36 | 3011.94 | 3410.66 | 3825.09 | 4463.34 | 4953.91 | 1761.76 | 2431.46 | 3176.41 | 3621.71 | 4046.73 | 4675.85 | 5168.13 |
|  | **60** |  |  |  |  |  |  |  | 1648.58 | 2034.66 | 2598.50 | 3005.61 | 3431.42 | 4091.44 | 4601.57 | 1583.93 | 2204.06 | 2893.77 | 3305.69 | 3698.62 | 4279.94 | 4734.62 |
|  | **80** |  |  |  |  |  |  |  | 1018.54 | 1357.29 | 1864.18 | 2236.45 | 3042.35 | 3702.16 | 4214.77 | 1264.65 | 1854.30 | 2511.68 | 2902.44 | 3273.97 | 3821.92 | 4249.39 |
|  |  |  |  |  |  |  |  |  |  |  |  |  | 2630.00 | 3246.45 | 3727.09 | 770.39 | 1356.40 | 2045.68 | 2453.09 | 2837.23 | 3398.91 | 3833.99 |
| **Peak expiratory flow, ml/s** | **20** |  |  |  |  |  |  |  | 5081.88 | 6457.54 | 8219.57 | 9376.97 | 10527.02 | 12249.46 | 13569.67 |  |  |  |  |  |  |  |
|  | **30** |  |  |  |  |  |  |  | 6067.23 | 7499.76 | 9336.34 | 10543.62 | 11743.77 | 13542.04 | 14920.88 |  |  |  |  |  |  |  |
|  | **40** |  |  |  |  |  |  |  | 5696.18 | 7127.42 | 8961.53 | 10166.77 | 11364.66 | 13159.15 | 14534.88 |  |  |  |  |  |  |  |
|  | **50** | 2643.56 | 3789.21 | 5519.49 | 6777.56 | 8074.03 | 9998.03 | 11386.17 | 4844.41 | 6297.66 | 8158.09 | 9379.47 | 10592.68 | 12409.11 | 13801.00 | 1970.19 | 3884.94 | 6603.50 | 8139.35 | 9484.87 | 11308.65 | 12639.45 |
|  | **60** | 1926.81 | 3033.09 | 4728.88 | 5971.08 | 7256.05 | 9169.18 | 10552.75 | 3669.60 | 5232.75 | 7236.74 | 8550.53 | 9854.13 | 11803.86 | 13296.60 | 1685.05 | 3400.98 | 5988.83 | 7488.06 | 8803.94 | 10582.64 | 11875.90 |
|  | **70** | 1201.44 | 2216.99 | 3826.50 | 5020.12 | 6261.51 | 8117.66 | 9464.09 | 2573.12 | 4196.48 | 6312.15 | 7700.30 | 9076.65 | 11133.20 | 12706.43 | 1250.19 | 2635.94 | 5047.67 | 6563.05 | 7911.99 | 9730.34 | 11042.35 |
|  | **80** | 570.97 | 1378.98 | 2784.33 | 3853.42 | 4975.03 | 6662.36 | 7891.21 | 1732.52 | 3294.82 | 5421.41 | 6824.90 | 8216.73 | 10295.40 | 11884.63 | 908.96 | 1971.95 | 4073.44 | 5541.82 | 6893.80 | 8729.76 | 10048.75 |
|  | **90** | 271.22 | 812.19 | 1866.79 | 2693.00 | 3567.16 | 4889.09 | 5854.83 |  |  |  |  |  |  |  |  |  |  |  |  |  |  |
|  | **100** | 117.49 | 422.00 | 1098.61 | 1648.38 | 2235.79 | 3129.07 | 3783.70 |  |  |  |  |  |  |  |  |  |  |  |  |  |  |
| **Fat-free mass index, kg/m^2^** | **20** |  |  |  |  |  |  |  | 13.7 | 15.0 | 17.1 | 18.9 | 21.0 | 24.9 | 28.7 |  |  |  |  |  |  |  |
|  | **30** |  |  |  |  |  |  |  | 14.9 | 16.3 | 18.5 | 20.3 | 22.5 | 26.6 | 30.5 |  |  |  |  |  |  |  |
|  | **40** |  |  |  |  |  |  |  | 15.3 | 16.6 | 18.8 | 20.6 | 22.7 | 26.5 | 30.1 |  |  |  |  |  |  |  |
|  | **50** |  |  |  |  |  |  |  | 15.3 | 16.6 | 18.7 | 20.4 | 22.5 | 26.1 | 29.5 | 15.3 | 16.4 | 18.0 | 19.3 | 20.6 | 22.9 | 24.9 |
|  | **60** |  |  |  |  |  |  |  | 14.8 | 16.1 | 18.3 | 20.0 | 22.1 | 25.9 | 29.5 | 15.4 | 16.4 | 17.8 | 18.9 | 20.0 | 22.0 | 23.6 |
|  | **70** |  |  |  |  |  |  |  |  |  |  |  |  |  |  | 15.4 | 16.3 | 17.6 | 18.6 | 19.7 | 21.5 | 23.0 |
|  | **80** |  |  |  |  |  |  |  |  |  |  |  |  |  |  | 15.0 | 15.8 | 17.1 | 18.0 | 19.1 | 20.8 | 22.1 |
| **Bone mineral content, kg** | **20** |  |  |  |  |  |  |  | 1.55 | 1.79 | 2.16 | 2.44 | 2.76 | 3.30 | 3.75 |  |  |  |  |  |  |  |
|  | **30** |  |  |  |  |  |  |  | 1.78 | 2.01 | 2.37 | 2.64 | 2.94 | 3.43 | 3.84 |  |  |  |  |  |  |  |
|  | **40** |  |  |  |  |  |  |  | 1.78 | 2.00 | 2.33 | 2.58 | 2.86 | 3.31 | 3.68 |  |  |  |  |  |  |  |
|  | **50** |  |  |  |  |  |  |  | 1.72 | 1.94 | 2.28 | 2.53 | 2.82 | 3.28 | 3.66 | 2.22 | 2.48 | 2.85 | 3.12 | 3.40 | 3.84 | 4.20 |
|  | **60** |  |  |  |  |  |  |  | 1.64 | 1.86 | 2.20 | 2.45 | 2.73 | 3.19 | 3.57 | 2.21 | 2.47 | 2.83 | 3.10 | 3.37 | 3.81 | 4.17 |
|  | **70** |  |  |  |  |  |  |  |  |  |  |  |  |  |  | 2.17 | 2.43 | 2.79 | 3.06 | 3.33 | 3.77 | 4.13 |
|  | **80** |  |  |  |  |  |  |  |  |  |  |  |  |  |  | 2.05 | 2.32 | 2.70 | 2.97 | 3.26 | 3.72 | 4.09 |
| **Bone mineral density, g/cm^2^** | **20** |  |  |  |  |  |  |  | 0.87 | 0.94 | 1.05 | 1.12 | 1.20 | 1.32 | 1.42 |  |  |  |  |  |  |  |
|  | **30** |  |  |  |  |  |  |  | 0.92 | 0.99 | 1.09 | 1.16 | 1.23 | 1.35 | 1.45 |  |  |  |  |  |  |  |
|  | **40** |  |  |  |  |  |  |  | 0.92 | 0.98 | 1.08 | 1.15 | 1.22 | 1.33 | 1.43 |  |  |  |  |  |  |  |
|  | **50** |  |  |  |  |  |  |  | 0.90 | 0.97 | 1.06 | 1.13 | 1.21 | 1.33 | 1.42 | 1.05 | 1.13 | 1.24 | 1.31 | 1.39 | 1.50 | 1.60 |
|  | **60** |  |  |  |  |  |  |  | 0.87 | 0.94 | 1.04 | 1.11 | 1.18 | 1.30 | 1.39 | 1.03 | 1.12 | 1.23 | 1.30 | 1.38 | 1.49 | 1.58 |
|  | **70** |  |  |  |  |  |  |  |  |  |  |  |  |  |  | 1.00 | 1.09 | 1.21 | 1.28 | 1.36 | 1.48 | 1.58 |
|  | **80** |  |  |  |  |  |  |  |  |  |  |  |  |  |  | 0.96 | 1.05 | 1.17 | 1.26 | 1.34 | 1.47 | 1.58 |
| **Frailty index_Lab** | **20** |  |  |  |  |  |  |  | 0.00 | 0.00 | 0.05 | 0.13 | 0.24 | 0.38 | 0.46 |  |  |  |  |  |  |  |
|  | **30** |  |  |  |  |  |  |  | 0.00 | 0.00 | 0.04 | 0.12 | 0.23 | 0.39 | 0.48 |  |  |  |  |  |  |  |
|  | **40** |  |  |  |  |  |  |  | 0.00 | 0.00 | 0.05 | 0.15 | 0.27 | 0.44 | 0.54 |  |  |  |  |  |  |  |
|  | **50** | 0.00 | 0.00 | 0.02 | 0.08 | 0.21 | 0.40 | 0.52 | 0.00 | 0.01 | 0.07 | 0.18 | 0.32 | 0.50 | 0.60 |  |  |  |  |  |  |  |
|  | **60** | 0.00 | 0.00 | 0.03 | 0.10 | 0.22 | 0.40 | 0.50 | 0.00 | 0.01 | 0.09 | 0.21 | 0.35 | 0.54 | 0.65 |  |  |  |  |  |  |  |
|  | **70** | 0.00 | 0.00 | 0.03 | 0.12 | 0.24 | 0.41 | 0.51 | 0.00 | 0.02 | 0.11 | 0.24 | 0.39 | 0.59 | 0.70 |  |  |  |  |  |  |  |
|  | **80** | 0.00 | 0.01 | 0.06 | 0.15 | 0.28 | 0.45 | 0.55 | 0.01 | 0.03 | 0.14 | 0.28 | 0.43 | 0.63 | 0.74 |  |  |  |  |  |  |  |
|  | **90** | 0.00 | 0.01 | 0.08 | 0.18 | 0.32 | 0.50 | 0.61 |  |  |  |  |  |  |  |  |  |  |  |  |  |  |
|  | **100** | 0.00 | 0.01 | 0.10 | 0.22 | 0.36 | 0.55 | 0.67 |  |  |  |  |  |  |  |  |  |  |  |  |  |  |

**Note:** FEV_1_, forced expiratory volume in the first second; CHARLS, the China Health and Retirement Longitudinal Study; NHANES, the National Health and Nutrition Examination Survey; UKB, the UK Biobank.

**Table S8 Age-specific percentile values of multi-systemic aging metrics for females in three datasets**

|  |  | **CHARLS** | | | | | | | **NHANES** | | | | | | | **UKB** | | | | | | |
| --- | --- | --- | --- | --- | --- | --- | --- | --- | --- | --- | --- | --- | --- | --- | --- | --- | --- | --- | --- | --- | --- | --- |
|  | **Age,**  **years** | **1st** | **5th** | **25th** | **50th** | **75th** | **95th** | **99th** | **1st** | **5th** | **25th** | **50th** | **75th** | **95th** | **99th** | **1st** | **5th** | **25th** | **50th** | **75th** | **95th** | **99th** |
| **Cognitive function score** | **50** | 2.0 | 4.4 | 8.3 | 11.1 | 14.0 | 18.2 | 21.2 |  |  |  |  |  |  |  | 2.1 | 3.3 | 5.2 | 6.6 | 8.1 | 10.2 | 11.8 |
|  | **60** | 0.6 | 2.3 | 6.1 | 9.1 | 12.3 | 17.0 | 20.4 | 7.0 | 9.8 | 14.3 | 17.7 | 21.5 | 27.3 | 31.8 | 2.2 | 3.4 | 5.2 | 6.6 | 7.9 | 10.0 | 11.5 |
|  | **70** | 0.2 | 1.0 | 4.0 | 7.1 | 10.6 | 15.9 | 19.8 | 5.1 | 7.7 | 12.1 | 15.5 | 19.2 | 25.2 | 29.8 | 2.0 | 3.1 | 5.0 | 6.3 | 7.7 | 9.7 | 11.1 |
|  | **80** | 0.1 | 0.5 | 2.4 | 4.8 | 7.6 | 12.2 | 15.7 | 3.9 | 6.2 | 10.2 | 13.3 | 16.7 | 22.2 | 26.5 | 1.6 | 2.6 | 4.1 | 5.2 | 6.3 | 8.0 | 9.2 |
|  | **90** | 0.0 | 0.3 | 1.5 | 3.2 | 5.4 | 9.1 | 11.9 |  |  |  |  |  |  |  |  |  |  |  |  |  |  |
|  | **100** | 0.0 | 0.2 | 1.0 | 2.2 | 3.8 | 6.5 | 8.6 |  |  |  |  |  |  |  |  |  |  |  |  |  |  |
| **Depression score** | **20** |  |  |  |  |  |  |  | 0.0 | 0.2 | 1.1 | 2.6 | 5.2 | 11.3 | 17.4 |  |  |  |  |  |  |  |
|  | **30** |  |  |  |  |  |  |  | 0.0 | 0.2 | 1.0 | 2.4 | 4.8 | 10.3 | 15.8 |  |  |  |  |  |  |  |
|  | **40** |  |  |  |  |  |  |  | 0.0 | 0.2 | 1.0 | 2.5 | 4.9 | 10.6 | 16.4 |  |  |  |  |  |  |  |
|  | **50** | 0.0 | 0.0 | 1.0 | 4.6 | 11.1 | 20.6 | 25.7 | 0.0 | 0.2 | 1.1 | 2.7 | 5.4 | 11.7 | 18.0 |  |  |  |  |  |  |  |
|  | **60** | 0.0 | 0.1 | 1.7 | 6.0 | 12.8 | 22.1 | 27.2 | 0.0 | 0.2 | 1.1 | 2.7 | 5.4 | 11.8 | 18.1 |  |  |  |  |  |  |  |
|  | **70** | 0.0 | 0.1 | 2.1 | 6.8 | 13.6 | 22.8 | 27.9 | 0.0 | 0.2 | 1.1 | 2.5 | 5.1 | 11.0 | 16.9 |  |  |  |  |  |  |  |
|  | **80** | 0.0 | 0.1 | 2.1 | 6.6 | 13.2 | 22.0 | 27.0 | 0.0 | 0.2 | 1.0 | 2.5 | 4.9 | 10.7 | 16.4 |  |  |  |  |  |  |  |
|  | **90** | 0.0 | 0.2 | 2.1 | 6.3 | 12.3 | 20.3 | 24.8 |  |  |  |  |  |  |  |  |  |  |  |  |  |  |
|  | **100** | 0.0 | 0.2 | 2.1 | 6.1 | 11.5 | 18.5 | 22.5 |  |  |  |  |  |  |  |  |  |  |  |  |  |  |
| **Systolic blood pressure, mmHg** | **20** |  |  |  |  |  |  |  | 88.1 | 93.4 | 101.2 | 107.0 | 113.4 | 124.4 | 134.2 |  |  |  |  |  |  |  |
|  | **30** |  |  |  |  |  |  |  | 88.1 | 94.2 | 103.2 | 110.1 | 117.7 | 131.0 | 143.2 |  |  |  |  |  |  |  |
|  | **40** |  |  |  |  |  |  |  | 88.6 | 95.6 | 106.2 | 114.3 | 123.6 | 140.2 | 155.9 |  |  |  |  |  |  |  |
|  | **50** | 89.0 | 96.7 | 109.4 | 119.8 | 131.6 | 151.9 | 168.9 | 90.7 | 98.7 | 110.8 | 120.4 | 131.4 | 151.7 | 171.3 | 94.7 | 102.0 | 113.5 | 122.5 | 132.5 | 148.8 | 161.9 |
|  | **60** | 91.3 | 99.7 | 113.8 | 125.5 | 138.9 | 162.2 | 182.1 | 93.9 | 102.6 | 116.1 | 126.9 | 139.4 | 162.9 | 186.0 | 98.1 | 106.5 | 120.2 | 130.9 | 143.0 | 163.1 | 179.5 |
|  | **70** | 95.0 | 104.2 | 119.9 | 132.8 | 147.9 | 174.4 | 197.3 | 97.2 | 106.7 | 121.5 | 133.3 | 147.3 | 173.6 | 200.1 | 103.7 | 113.0 | 128.2 | 140.2 | 153.8 | 176.5 | 195.2 |
|  | **80** | 97.7 | 107.7 | 124.7 | 139.0 | 155.7 | 185.5 | 211.6 | 99.7 | 110.1 | 126.5 | 139.9 | 155.9 | 186.7 | 218.5 | 111.6 | 121.4 | 137.4 | 150.1 | 164.3 | 188.1 | 207.8 |
|  | **90** | 98.0 | 109.0 | 128.0 | 144.1 | 163.5 | 198.6 | 230.2 |  |  |  |  |  |  |  |  |  |  |  |  |  |  |
|  | **100** | 96.5 | 108.5 | 129.7 | 148.2 | 170.8 | 213.1 | 252.7 |  |  |  |  |  |  |  |  |  |  |  |  |  |  |
| **Diastolic blood pressure, mmHg** | **20** |  |  |  |  |  |  |  | 41.7 | 49.2 | 58.3 | 64.2 | 70.3 | 79.9 | 88.2 |  |  |  |  |  |  |  |
|  | **30** |  |  |  |  |  |  |  | 45.5 | 53.4 | 63.0 | 69.2 | 75.5 | 85.6 | 94.3 |  |  |  |  |  |  |  |
|  | **40** |  |  |  |  |  |  |  | 47.6 | 56.0 | 66.2 | 72.7 | 79.4 | 90.1 | 99.4 |  |  |  |  |  |  |  |
|  | **50** | 52.1 | 57.7 | 66.7 | 73.7 | 81.4 | 93.8 | 103.5 | 48.3 | 56.9 | 67.5 | 74.3 | 81.2 | 92.3 | 101.8 | 55.7 | 61.3 | 69.7 | 76.0 | 82.6 | 93.0 | 100.9 |
|  | **60** | 52.4 | 58.0 | 67.0 | 74.0 | 81.6 | 94.0 | 103.6 | 46.2 | 55.1 | 65.9 | 73.0 | 80.1 | 91.6 | 101.5 | 56.3 | 62.0 | 70.7 | 77.1 | 83.9 | 94.6 | 102.7 |
|  | **70** | 51.7 | 57.3 | 66.3 | 73.2 | 80.9 | 93.2 | 102.8 | 41.4 | 50.7 | 62.2 | 69.6 | 77.2 | 89.4 | 99.9 | 55.4 | 61.2 | 70.0 | 76.6 | 83.6 | 94.5 | 102.9 |
|  | **80** | 50.4 | 56.1 | 65.1 | 72.2 | 80.0 | 92.6 | 102.6 | 35.8 | 45.6 | 57.7 | 65.6 | 73.6 | 86.7 | 97.9 | 53.7 | 59.7 | 68.8 | 75.7 | 83.1 | 94.6 | 103.5 |
|  | **90** | 48.5 | 54.4 | 64.1 | 71.7 | 80.1 | 93.9 | 104.9 |  |  |  |  |  |  |  |  |  |  |  |  |  |  |
|  | **100** | 47.3 | 53.5 | 63.6 | 71.6 | 80.6 | 95.4 | 107.3 |  |  |  |  |  |  |  |  |  |  |  |  |  |  |
| **Pulse** | **20** |  |  |  |  |  |  |  | 54.9 | 61.0 | 69.9 | 76.6 | 84.0 | 96.8 | 108.3 |  |  |  |  |  |  |  |
|  | **30** |  |  |  |  |  |  |  | 52.7 | 58.7 | 67.5 | 74.1 | 81.4 | 94.2 | 105.8 |  |  |  |  |  |  |  |
|  | **40** |  |  |  |  |  |  |  | 51.5 | 57.5 | 66.3 | 72.9 | 80.3 | 93.2 | 104.9 |  |  |  |  |  |  |  |
|  | **50** | 53.9 | 59.5 | 67.6 | 73.5 | 79.9 | 90.9 | 100.9 | 50.2 | 56.2 | 65.1 | 71.8 | 79.3 | 92.4 | 104.4 | 49.4 | 55.4 | 64.7 | 72.0 | 80.2 | 94.7 | 107.6 |
|  | **60** | 53.3 | 59.0 | 67.2 | 73.2 | 79.8 | 91.2 | 101.5 | 48.8 | 54.8 | 63.8 | 70.5 | 78.1 | 91.5 | 103.7 | 48.8 | 54.6 | 63.5 | 70.4 | 78.3 | 91.9 | 104.1 |
|  | **70** | 52.6 | 58.7 | 67.6 | 74.1 | 81.3 | 93.9 | 105.5 | 47.8 | 53.8 | 62.7 | 69.4 | 77.1 | 90.5 | 102.8 | 49.2 | 55.0 | 63.9 | 70.8 | 78.6 | 92.2 | 104.3 |
|  | **80** | 53.1 | 59.5 | 68.8 | 75.6 | 83.3 | 96.6 | 108.9 | 46.8 | 52.8 | 61.7 | 68.5 | 76.1 | 89.6 | 102.0 | 48.1 | 53.8 | 62.7 | 69.6 | 77.4 | 91.0 | 103.1 |
|  | **90** | 52.4 | 59.1 | 68.8 | 76.1 | 84.1 | 98.4 | 111.5 |  |  |  |  |  |  |  |  |  |  |  |  |  |  |
|  | **100** | 51.3 | 58.2 | 68.2 | 75.8 | 84.3 | 99.3 | 113.4 |  |  |  |  |  |  |  |  |  |  |  |  |  |  |
| **Body mass index, kg/m^2^** | **20** |  |  |  |  |  |  |  | 15.4 | 17.4 | 21.1 | 24.6 | 29.3 | 39.8 | 52.0 |  |  |  |  |  |  |  |
|  | **30** |  |  |  |  |  |  |  | 16.9 | 19.2 | 23.5 | 27.6 | 33.4 | 46.5 | 62.7 |  |  |  |  |  |  |  |
|  | **40** |  |  |  |  |  |  |  | 17.9 | 20.2 | 24.7 | 29.0 | 34.9 | 48.3 | 64.5 |  |  |  |  |  |  |  |
|  | **50** | 17.4 | 19.3 | 22.3 | 24.5 | 27.0 | 31.1 | 34.5 | 18.4 | 20.7 | 25.2 | 29.5 | 35.3 | 48.1 | 63.4 | 18.1 | 19.8 | 22.8 | 25.5 | 28.9 | 35.9 | 43.2 |
|  | **60** | 16.7 | 18.7 | 21.7 | 24.1 | 26.6 | 31.0 | 34.6 | 18.6 | 20.9 | 25.3 | 29.4 | 34.8 | 46.8 | 60.6 | 18.2 | 19.9 | 22.9 | 25.5 | 28.9 | 35.7 | 42.8 |
|  | **70** | 15.9 | 17.9 | 21.0 | 23.4 | 26.1 | 30.6 | 34.5 | 18.7 | 20.9 | 25.0 | 28.7 | 33.7 | 44.1 | 55.6 | 18.4 | 20.0 | 22.8 | 25.3 | 28.3 | 34.4 | 40.6 |
|  | **80** | 14.8 | 16.8 | 19.8 | 22.2 | 24.8 | 29.3 | 33.1 | 18.3 | 20.4 | 24.1 | 27.5 | 31.9 | 40.8 | 50.2 | 18.3 | 19.9 | 22.4 | 24.7 | 27.4 | 32.6 | 37.7 |
|  | **90** | 14.2 | 16.0 | 18.7 | 20.8 | 23.2 | 27.1 | 30.5 |  |  |  |  |  |  |  |  |  |  |  |  |  |  |
|  | **100** | 13.8 | 15.4 | 17.8 | 19.6 | 21.6 | 25.0 | 27.8 |  |  |  |  |  |  |  |  |  |  |  |  |  |  |
| **Waist circumference, cm** | **20** |  |  |  |  |  |  |  | 60.6 | 65.9 | 75.6 | 84.3 | 95.4 | 118.2 | 142.8 |  |  |  |  |  |  |  |
|  | **30** |  |  |  |  |  |  |  | 65.5 | 71.6 | 82.6 | 92.7 | 105.7 | 133.1 | 163.7 |  |  |  |  |  |  |  |
|  | **40** |  |  |  |  |  |  |  | 68.6 | 74.8 | 86.0 | 96.2 | 109.3 | 136.6 | 166.4 |  |  |  |  |  |  |  |
|  | **50** | 50.9 | 66.4 | 79.4 | 86.1 | 92.2 | 102.2 | 111.7 | 70.9 | 77.0 | 88.1 | 97.9 | 110.5 | 136.1 | 163.1 | 60.3 | 65.1 | 73.4 | 80.4 | 88.6 | 103.7 | 117.3 |
|  | **60** | 49.1 | 65.7 | 79.8 | 86.9 | 93.5 | 104.1 | 114.2 | 72.6 | 78.7 | 89.3 | 98.8 | 110.6 | 134.0 | 157.9 | 60.8 | 65.7 | 74.2 | 81.4 | 89.9 | 105.4 | 119.6 |
|  | **70** | 45.9 | 63.8 | 79.3 | 87.0 | 94.1 | 105.6 | 116.4 | 73.9 | 79.7 | 89.7 | 98.5 | 109.3 | 130.1 | 150.6 | 61.9 | 66.7 | 74.9 | 81.8 | 89.9 | 104.6 | 117.7 |
|  | **80** | 45.0 | 62.9 | 78.4 | 86.1 | 93.3 | 104.7 | 115.5 | 73.9 | 79.4 | 88.9 | 97.0 | 106.9 | 125.5 | 143.4 | 61.9 | 66.8 | 74.9 | 81.7 | 89.8 | 104.2 | 117.1 |
|  | **90** | 49.2 | 64.4 | 77.3 | 83.8 | 89.9 | 99.7 | 109.0 |  |  |  |  |  |  |  |  |  |  |  |  |  |  |
|  | **100** | 54.7 | 66.2 | 76.0 | 81.0 | 85.7 | 93.5 | 101.0 |  |  |  |  |  |  |  |  |  |  |  |  |  |  |
| **Grip strength, kg** | **20** |  |  |  |  |  |  |  | 18.0 | 21.3 | 26.1 | 29.7 | 33.6 | 40.1 | 45.6 |  |  |  |  |  |  |  |
|  | **30** |  |  |  |  |  |  |  | 20.7 | 24.1 | 28.9 | 32.5 | 36.3 | 42.7 | 48.2 |  |  |  |  |  |  |  |
|  | **40** |  |  |  |  |  |  |  | 20.4 | 23.7 | 28.4 | 31.8 | 35.6 | 41.7 | 47.0 |  |  |  |  |  |  |  |
|  | **50** | 11.7 | 17.1 | 23.6 | 27.7 | 31.7 | 37.7 | 42.5 | 19.2 | 22.3 | 26.9 | 30.2 | 33.8 | 39.8 | 44.9 | 12.6 | 17.8 | 24.0 | 27.9 | 31.8 | 37.8 | 42.6 |
|  | **60** | 9.4 | 14.6 | 20.9 | 24.9 | 28.7 | 34.5 | 39.0 | 17.2 | 20.2 | 24.7 | 28.0 | 31.5 | 37.5 | 42.6 | 11.4 | 16.1 | 21.9 | 25.5 | 29.1 | 34.6 | 39.1 |
|  | **70** | 6.4 | 11.4 | 17.6 | 21.5 | 25.4 | 31.1 | 35.6 | 14.4 | 17.3 | 21.6 | 24.9 | 28.4 | 34.4 | 39.6 | 9.4 | 14.0 | 19.6 | 23.2 | 26.6 | 32.0 | 36.3 |
|  | **80** | 3.4 | 7.8 | 13.9 | 17.9 | 21.7 | 27.5 | 31.9 | 11.1 | 13.8 | 17.9 | 21.1 | 24.6 | 30.6 | 35.9 | 7.3 | 11.7 | 17.1 | 20.5 | 23.9 | 29.1 | 33.3 |
|  | **90** | 1.5 | 4.5 | 10.7 | 15.1 | 19.4 | 25.9 | 31.0 |  |  |  |  |  |  |  |  |  |  |  |  |  |  |
|  | **100** | 0.8 | 2.8 | 8.8 | 14.0 | 19.4 | 27.7 | 34.2 |  |  |  |  |  |  |  |  |  |  |  |  |  |  |
| **FEV_1_, ml** | **20** |  |  |  |  |  |  |  | 1875.85 | 2195.58 | 2661.64 | 2995.45 | 3339.89 | 3859.73 | 4246.70 |  |  |  |  |  |  |  |
|  | **30** |  |  |  |  |  |  |  | 1976.30 | 2300.59 | 2772.55 | 3110.17 | 3458.26 | 3983.14 | 4373.59 |  |  |  |  |  |  |  |
|  | **40** |  |  |  |  |  |  |  | 1713.76 | 2030.72 | 2494.37 | 2827.37 | 3171.61 | 3692.09 | 4080.17 |  |  |  |  |  |  |  |
|  | **50** |  |  |  |  |  |  |  | 1446.88 | 1736.73 | 2162.31 | 2468.81 | 2786.25 | 3267.10 | 3626.20 | 1285.18 | 1785.45 | 2314.37 | 2622.01 | 2916.57 | 3368.11 | 3744.55 |
|  | **60** |  |  |  |  |  |  |  | 1244.35 | 1501.08 | 1878.55 | 2150.70 | 2432.75 | 2860.29 | 3179.77 | 1105.94 | 1566.39 | 2053.87 | 2336.93 | 2607.64 | 3022.11 | 3367.28 |
|  | **70** |  |  |  |  |  |  |  | 1022.99 | 1265.68 | 1625.04 | 1885.49 | 2156.33 | 2568.24 | 2876.89 | 812.24 | 1253.11 | 1727.11 | 2000.97 | 2261.66 | 2658.89 | 2988.34 |
|  | **80** |  |  |  |  |  |  |  | 808.99 | 1046.77 | 1403.20 | 1663.77 | 1936.18 | 2352.66 | 2666.08 | 590.21 | 980.24 | 1410.42 | 1658.63 | 1894.14 | 2251.72 | 2547.38 |
| **Peak expiratory flow, ml/s** | **20** |  |  |  |  |  |  |  | 3964.63 | 4944.61 | 6264.18 | 7148.28 | 8017.97 | 9263.18 | 10146.26 |  |  |  |  |  |  |  |
|  | **30** |  |  |  |  |  |  |  | 4518.80 | 5496.37 | 6816.65 | 7702.98 | 8575.85 | 9826.94 | 10714.93 |  |  |  |  |  |  |  |
|  | **40** |  |  |  |  |  |  |  | 4073.53 | 5094.79 | 6469.46 | 7390.29 | 8296.00 | 9592.63 | 10512.10 |  |  |  |  |  |  |  |
|  | **50** | 1890.40 | 2616.46 | 3727.51 | 4545.06 | 5395.04 | 6668.70 | 7595.72 | 3400.73 | 4454.57 | 5866.20 | 6808.73 | 7734.09 | 9056.70 | 9993.39 | 1381.94 | 2712.97 | 4603.55 | 5663.25 | 6595.18 | 7895.80 | 8897.17 |
|  | **60** | 1586.07 | 2304.05 | 3413.08 | 4233.75 | 5089.62 | 6375.69 | 7313.70 | 2820.39 | 3852.59 | 5230.65 | 6148.48 | 7048.33 | 8332.88 | 9241.76 | 1209.29 | 2398.29 | 4134.55 | 5119.57 | 5986.66 | 7195.14 | 8123.85 |
|  | **70** | 1065.87 | 1723.57 | 2761.50 | 3538.22 | 4353.01 | 5583.47 | 6484.23 | 2152.65 | 3176.79 | 4541.49 | 5447.31 | 6333.55 | 7596.33 | 8488.55 | 827.73 | 1729.15 | 3299.41 | 4287.38 | 5173.42 | 6403.52 | 7338.66 |
|  | **80** | 714.99 | 1274.34 | 2176.90 | 2858.77 | 3577.25 | 4666.25 | 5465.54 | 1425.44 | 2449.15 | 3836.82 | 4755.91 | 5652.76 | 6927.43 | 7826.31 | 622.79 | 1325.67 | 2658.75 | 3559.05 | 4384.33 | 5534.57 | 6405.51 |
|  | **90** | 410.77 | 900.44 | 1741.49 | 2390.24 | 3079.62 | 4131.23 | 4906.47 |  |  |  |  |  |  |  |  |  |  |  |  |  |  |
|  | **100** | 190.68 | 596.64 | 1423.14 | 2091.50 | 2812.73 | 3924.34 | 4749.16 |  |  |  |  |  |  |  |  |  |  |  |  |  |  |
| **Fat-free mass index, kg/m^2^** | **20** |  |  |  |  |  |  |  | 11.8 | 12.8 | 14.6 | 16.2 | 18.1 | 22.0 | 25.8 |  |  |  |  |  |  |  |
|  | **30** |  |  |  |  |  |  |  | 12.3 | 13.4 | 15.4 | 17.1 | 19.3 | 23.8 | 28.3 |  |  |  |  |  |  |  |
|  | **40** |  |  |  |  |  |  |  | 12.5 | 13.6 | 15.6 | 17.4 | 19.7 | 24.1 | 28.7 |  |  |  |  |  |  |  |
|  | **50** |  |  |  |  |  |  |  | 12.5 | 13.7 | 15.6 | 17.4 | 19.6 | 23.9 | 28.3 | 12.9 | 13.9 | 15.2 | 16.1 | 17.1 | 19.1 | 21.4 |
|  | **60** |  |  |  |  |  |  |  | 12.3 | 13.4 | 15.3 | 17.0 | 19.2 | 23.3 | 27.5 | 12.6 | 13.5 | 14.8 | 15.7 | 16.7 | 18.6 | 20.7 |
|  | **70** |  |  |  |  |  |  |  |  |  |  |  |  |  |  | 12.8 | 13.7 | 14.8 | 15.6 | 16.5 | 18.2 | 20.0 |
|  | **80** |  |  |  |  |  |  |  |  |  |  |  |  |  |  | 12.8 | 13.7 | 14.9 | 15.7 | 16.7 | 18.5 | 20.4 |
| **Bone mineral content, kg** | **20** |  |  |  |  |  |  |  | 1.38 | 1.55 | 1.82 | 2.04 | 2.28 | 2.68 | 3.00 |  |  |  |  |  |  |  |
|  | **30** |  |  |  |  |  |  |  | 1.50 | 1.66 | 1.93 | 2.14 | 2.38 | 2.76 | 3.06 |  |  |  |  |  |  |  |
|  | **40** |  |  |  |  |  |  |  | 1.48 | 1.65 | 1.91 | 2.12 | 2.36 | 2.74 | 3.04 |  |  |  |  |  |  |  |
|  | **50** |  |  |  |  |  |  |  | 1.39 | 1.55 | 1.82 | 2.04 | 2.28 | 2.67 | 2.99 | 1.77 | 1.96 | 2.23 | 2.44 | 2.66 | 3.03 | 3.33 |
|  | **60** |  |  |  |  |  |  |  | 1.18 | 1.35 | 1.63 | 1.86 | 2.12 | 2.55 | 2.91 | 1.58 | 1.75 | 2.00 | 2.19 | 2.40 | 2.75 | 3.03 |
|  | **70** |  |  |  |  |  |  |  |  |  |  |  |  |  |  | 1.48 | 1.65 | 1.90 | 2.09 | 2.29 | 2.64 | 2.92 |
|  | **80** |  |  |  |  |  |  |  |  |  |  |  |  |  |  | 1.38 | 1.55 | 1.81 | 2.00 | 2.22 | 2.57 | 2.88 |
| **Bone mineral density, g/cm^2^** | **20** |  |  |  |  |  |  |  | 0.86 | 0.92 | 1.00 | 1.07 | 1.14 | 1.24 | 1.31 |  |  |  |  |  |  |  |
|  | **30** |  |  |  |  |  |  |  | 0.90 | 0.95 | 1.04 | 1.10 | 1.16 | 1.26 | 1.34 |  |  |  |  |  |  |  |
|  | **40** |  |  |  |  |  |  |  | 0.89 | 0.95 | 1.04 | 1.10 | 1.17 | 1.27 | 1.34 |  |  |  |  |  |  |  |
|  | **50** |  |  |  |  |  |  |  | 0.85 | 0.91 | 1.00 | 1.07 | 1.14 | 1.25 | 1.33 | 0.98 | 1.05 | 1.15 | 1.21 | 1.29 | 1.40 | 1.49 |
|  | **60** |  |  |  |  |  |  |  | 0.76 | 0.82 | 0.93 | 1.00 | 1.08 | 1.21 | 1.30 | 0.87 | 0.94 | 1.04 | 1.12 | 1.19 | 1.31 | 1.41 |
|  | **70** |  |  |  |  |  |  |  |  |  |  |  |  |  |  | 0.82 | 0.89 | 0.99 | 1.07 | 1.15 | 1.27 | 1.37 |
|  | **80** |  |  |  |  |  |  |  |  |  |  |  |  |  |  | 0.76 | 0.84 | 0.95 | 1.03 | 1.12 | 1.25 | 1.36 |
| **Frailty index_Lab** | **20** |  |  |  |  |  |  |  | 0.00 | 0.00 | 0.03 | 0.11 | 0.23 | 0.39 | 0.47 |  |  |  |  |  |  |  |
|  | **30** |  |  |  |  |  |  |  | 0.00 | 0.00 | 0.02 | 0.10 | 0.23 | 0.41 | 0.51 |  |  |  |  |  |  |  |
|  | **40** |  |  |  |  |  |  |  | 0.00 | 0.00 | 0.02 | 0.10 | 0.24 | 0.44 | 0.56 |  |  |  |  |  |  |  |
|  | **50** | 0.00 | 0.00 | 0.01 | 0.05 | 0.16 | 0.34 | 0.45 | 0.00 | 0.00 | 0.02 | 0.11 | 0.26 | 0.48 | 0.60 |  |  |  |  |  |  |  |
|  | **60** | 0.00 | 0.00 | 0.01 | 0.06 | 0.19 | 0.40 | 0.52 | 0.00 | 0.00 | 0.03 | 0.12 | 0.28 | 0.51 | 0.64 |  |  |  |  |  |  |  |
|  | **70** | 0.00 | 0.00 | 0.02 | 0.09 | 0.22 | 0.42 | 0.54 | 0.00 | 0.00 | 0.04 | 0.15 | 0.31 | 0.53 | 0.65 |  |  |  |  |  |  |  |
|  | **80** | 0.00 | 0.00 | 0.03 | 0.11 | 0.24 | 0.43 | 0.54 | 0.00 | 0.01 | 0.07 | 0.19 | 0.34 | 0.53 | 0.64 |  |  |  |  |  |  |  |
|  | **90** | 0.00 | 0.00 | 0.05 | 0.13 | 0.25 | 0.41 | 0.51 |  |  |  |  |  |  |  |  |  |  |  |  |  |  |
|  | **100** | 0.00 | 0.01 | 0.06 | 0.14 | 0.24 | 0.37 | 0.45 |  |  |  |  |  |  |  |  |  |  |  |  |  |  |

**Note:** FEV_1_, forced expiratory volume in the first second; CHARLS, the China Health and Retirement Longitudinal Study; NHANES, the National Health and Nutrition Examination Survey; UKB, the UK Biobank.

**Table S9** **Age-specific percentile values of multi-systemic aging metrics for participants in the “low-income” group in three datasets**

|  |  | **CHARLS** | | | | | | | **NHANES** | | | | | | | **UKB** | | | | | | |
| --- | --- | --- | --- | --- | --- | --- | --- | --- | --- | --- | --- | --- | --- | --- | --- | --- | --- | --- | --- | --- | --- | --- |
|  | **Age,**  **years** | **1st** | **5th** | **25th** | **50th** | **75th** | **95th** | **99th** | **1st** | **5th** | **25th** | **50th** | **75th** | **95th** | **99th** | **1st** | **5th** | **25th** | **50th** | **75th** | **95th** | **99th** |
| **Cognitive function score** | **50** | 3.1 | 5.4 | 9.6 | 12.4 | 14.7 | 17.4 | 18.8 |  |  |  |  |  |  |  | 1.8 | 3.1 | 5.1 | 6.5 | 8.1 | 10.3 | 12.0 |
|  | **60** | 1.3 | 3.1 | 7.2 | 10.4 | 13.1 | 16.1 | 17.6 | 5.4 | 8.1 | 12.5 | 16.1 | 20.0 | 26.4 | 31.2 | 1.9 | 3.1 | 5.0 | 6.4 | 7.9 | 10.1 | 11.7 |
|  | **70** | 0.6 | 1.8 | 5.5 | 8.8 | 11.8 | 15.0 | 16.6 | 4.2 | 6.6 | 10.8 | 14.2 | 18.0 | 24.2 | 29.0 | 1.8 | 3.0 | 4.9 | 6.3 | 7.7 | 9.9 | 11.5 |
|  | **80** | 0.0 | 0.3 | 2.0 | 4.9 | 8.3 | 12.0 | 13.7 | 3.7 | 5.8 | 9.4 | 12.3 | 15.6 | 20.8 | 24.9 | 1.3 | 2.4 | 4.2 | 5.5 | 6.9 | 9.0 | 10.5 |
|  | **90** | 0.0 | 0.0 | 0.1 | 0.8 | 3.4 | 8.1 | 10.1 |  |  |  |  |  |  |  |  |  |  |  |  |  |  |
| **Depression score** | **20** |  |  |  |  |  |  |  | 0.0 | 0.2 | 1.0 | 2.4 | 4.7 | 10.2 | 15.7 |  |  |  |  |  |  |  |
|  | **30** |  |  |  |  |  |  |  | 0.0 | 0.2 | 1.1 | 2.7 | 5.4 | 11.7 | 18.0 |  |  |  |  |  |  |  |
|  | **40** |  |  |  |  |  |  |  | 0.0 | 0.2 | 1.3 | 3.2 | 6.4 | 13.9 | 21.3 |  |  |  |  |  |  |  |
|  | **50** | 0.1 | 0.4 | 2.1 | 5.1 | 10.2 | 22.1 | 30.0 | 0.1 | 0.3 | 1.5 | 3.7 | 7.3 | 15.8 | 24.3 |  |  |  |  |  |  |  |
|  | **60** | 0.1 | 0.4 | 2.4 | 5.8 | 11.6 | 25.1 | 30.0 | 0.1 | 0.3 | 1.5 | 3.5 | 7.1 | 15.3 | 23.5 |  |  |  |  |  |  |  |
|  | **70** | 0.1 | 0.5 | 2.6 | 6.2 | 12.3 | 26.7 | 30.0 | 0.0 | 0.2 | 1.2 | 2.9 | 5.8 | 12.5 | 19.3 |  |  |  |  |  |  |  |
|  | **80** | 0.1 | 0.5 | 2.7 | 6.5 | 12.9 | 27.9 | 30.0 | 0.0 | 0.2 | 1.0 | 2.4 | 4.7 | 10.2 | 15.7 |  |  |  |  |  |  |  |
|  | **90** | 0.1 | 0.6 | 3.4 | 8.1 | 16.2 | 30.0 | 30.0 |  |  |  |  |  |  |  |  |  |  |  |  |  |  |
|  | **100** |  |  |  |  |  |  |  |  |  |  |  |  |  |  |  |  |  |  |  |  |  |
| **Systolic blood pressure, mmHg** | **20** |  |  |  |  |  |  |  | 90.1 | 95.9 | 104.6 | 111.2 | 118.6 | 131.0 | 141.7 |  |  |  |  |  |  |  |
|  | **30** |  |  |  |  |  |  |  | 90.9 | 97.4 | 107.4 | 115.0 | 123.7 | 138.5 | 151.5 |  |  |  |  |  |  |  |
|  | **40** |  |  |  |  |  |  |  | 90.8 | 98.1 | 109.6 | 118.7 | 129.0 | 147.2 | 163.6 |  |  |  |  |  |  |  |
|  | **50** | 89.6 | 97.6 | 110.8 | 121.3 | 133.2 | 153.3 | 169.9 | 91.7 | 100.0 | 113.1 | 123.6 | 135.9 | 157.9 | 178.5 | 97.8 | 105.5 | 117.8 | 127.4 | 137.9 | 155.0 | 168.6 |
|  | **60** | 90.6 | 99.5 | 114.3 | 126.3 | 140.1 | 163.7 | 183.5 | 94.2 | 103.2 | 117.7 | 129.5 | 143.3 | 168.7 | 192.7 | 100.0 | 108.7 | 122.7 | 133.7 | 145.9 | 165.9 | 182.1 |
|  | **70** | 92.7 | 102.3 | 118.4 | 131.7 | 147.0 | 173.6 | 196.2 | 98.1 | 107.7 | 123.1 | 135.6 | 150.2 | 177.3 | 203.1 | 103.9 | 113.4 | 128.7 | 140.8 | 154.3 | 176.7 | 194.9 |
|  | **80** | 98.4 | 107.9 | 123.6 | 136.4 | 151.1 | 176.0 | 197.0 | 101.4 | 111.5 | 128.0 | 141.5 | 157.4 | 187.0 | 215.5 | 114.8 | 124.2 | 139.3 | 151.0 | 164.0 | 185.2 | 202.1 |
|  | **90** | 105.1 | 115.1 | 131.6 | 144.9 | 160.1 | 185.9 | 207.5 |  |  |  |  |  |  |  |  |  |  |  |  |  |  |
| **Diastolic blood pressure, mmHg** | **20** |  |  |  |  |  |  |  | 39.9 | 47.9 | 57.8 | 64.3 | 70.9 | 81.3 | 90.0 |  |  |  |  |  |  |  |
|  | **30** |  |  |  |  |  |  |  | 45.2 | 53.5 | 63.8 | 70.5 | 77.2 | 87.9 | 96.9 |  |  |  |  |  |  |  |
|  | **40** |  |  |  |  |  |  |  | 47.4 | 56.3 | 67.2 | 74.4 | 81.6 | 93.1 | 102.7 |  |  |  |  |  |  |  |
|  | **50** | 52.0 | 58.2 | 68.0 | 75.4 | 83.5 | 96.2 | 105.9 | 47.8 | 56.9 | 68.3 | 75.7 | 83.1 | 95.0 | 104.9 | 57.6 | 63.4 | 72.3 | 78.8 | 85.7 | 96.2 | 104.0 |
|  | **60** | 53.1 | 59.0 | 68.2 | 75.2 | 82.7 | 94.5 | 103.5 | 45.0 | 54.5 | 66.2 | 73.9 | 81.7 | 94.0 | 104.4 | 57.9 | 63.7 | 72.5 | 79.1 | 85.9 | 96.4 | 104.2 |
|  | **70** | 50.5 | 56.4 | 65.8 | 72.9 | 80.6 | 92.7 | 102.0 | 39.4 | 49.2 | 61.5 | 69.5 | 77.6 | 90.6 | 101.5 | 55.7 | 61.6 | 70.7 | 77.5 | 84.6 | 95.5 | 103.7 |
|  | **80** | 50.4 | 55.9 | 64.5 | 71.0 | 77.9 | 88.8 | 97.1 | 32.1 | 42.4 | 55.3 | 63.8 | 72.4 | 86.2 | 97.8 | 54.3 | 60.5 | 69.9 | 77.0 | 84.4 | 95.9 | 104.4 |
|  | **90** | 43.5 | 49.6 | 59.2 | 66.6 | 74.7 | 87.7 | 97.7 |  |  |  |  |  |  |  |  |  |  |  |  |  |  |
| **Pulse** | **20** |  |  |  |  |  |  |  | 51.2 | 57.1 | 66.4 | 73.8 | 82.2 | 96.9 | 109.8 |  |  |  |  |  |  |  |
|  | **30** |  |  |  |  |  |  |  | 50.0 | 55.8 | 65.0 | 72.2 | 80.5 | 94.9 | 107.7 |  |  |  |  |  |  |  |
|  | **40** |  |  |  |  |  |  |  | 50.5 | 56.2 | 65.3 | 72.5 | 80.6 | 94.7 | 107.2 |  |  |  |  |  |  |  |
|  | **50** | 55.3 | 60.1 | 68.0 | 74.3 | 81.5 | 93.6 | 103.7 | 50.0 | 55.8 | 65.1 | 72.4 | 80.7 | 95.3 | 108.2 | 47.3 | 53.5 | 63.1 | 70.6 | 79.2 | 94.2 | 107.6 |
|  | **60** | 54.2 | 58.8 | 66.4 | 72.4 | 79.3 | 90.8 | 100.5 | 48.9 | 54.7 | 64.0 | 71.4 | 79.8 | 94.6 | 107.8 | 46.7 | 52.7 | 62.2 | 69.6 | 78.0 | 92.7 | 105.9 |
|  | **70** | 51.7 | 56.5 | 64.2 | 70.5 | 77.6 | 89.8 | 100.0 | 47.3 | 53.1 | 62.2 | 69.5 | 77.8 | 92.5 | 105.6 | 45.9 | 51.9 | 61.2 | 68.5 | 76.8 | 91.4 | 104.3 |
|  | **80** | 53.3 | 58.2 | 66.3 | 72.9 | 80.4 | 93.2 | 104.0 | 45.6 | 51.3 | 60.3 | 67.5 | 75.7 | 90.3 | 103.4 | 44.6 | 50.7 | 60.2 | 67.8 | 76.4 | 91.5 | 105.1 |
|  | **90** | 53.6 | 59.1 | 68.1 | 75.6 | 84.2 | 99.3 | 112.2 |  |  |  |  |  |  |  |  |  |  |  |  |  |  |
| **Body mass index, kg/m^2^** | **20** |  |  |  |  |  |  |  | 15.7 | 17.7 | 21.3 | 24.8 | 29.5 | 40.2 | 53.4 |  |  |  |  |  |  |  |
|  | **30** |  |  |  |  |  |  |  | 17.6 | 19.8 | 24.0 | 28.0 | 33.5 | 46.2 | 62.3 |  |  |  |  |  |  |  |
|  | **40** |  |  |  |  |  |  |  | 18.7 | 21.0 | 25.3 | 29.4 | 34.9 | 47.5 | 62.9 |  |  |  |  |  |  |  |
|  | **50** | 17.5 | 19.2 | 21.9 | 23.9 | 26.2 | 30.3 | 34.1 | 18.8 | 21.1 | 25.4 | 29.4 | 34.9 | 47.3 | 62.4 | 18.7 | 20.6 | 23.8 | 26.7 | 30.2 | 37.0 | 44.0 |
|  | **60** | 16.1 | 18.0 | 20.9 | 23.2 | 25.8 | 30.7 | 35.4 | 18.5 | 20.7 | 24.9 | 28.9 | 34.3 | 46.4 | 61.2 | 18.6 | 20.5 | 23.7 | 26.4 | 29.9 | 36.6 | 43.3 |
|  | **70** | 16.0 | 17.7 | 20.3 | 22.3 | 24.6 | 28.7 | 32.5 | 18.2 | 20.4 | 24.4 | 28.2 | 33.2 | 44.4 | 57.7 | 18.9 | 20.6 | 23.6 | 26.2 | 29.4 | 35.3 | 41.2 |
|  | **80** | 15.2 | 16.8 | 19.1 | 20.9 | 22.9 | 26.5 | 29.9 | 17.7 | 19.7 | 23.5 | 27.1 | 31.8 | 42.1 | 54.0 | 18.7 | 20.3 | 22.8 | 25.0 | 27.5 | 32.0 | 36.3 |
|  | **90** | 14.3 | 15.7 | 17.7 | 19.2 | 21.0 | 24.0 | 26.8 |  |  |  |  |  |  |  |  |  |  |  |  |  |  |
| **Waist circumference, cm** | **20** |  |  |  |  |  |  |  | 61.5 | 66.8 | 76.6 | 85.5 | 97.3 | 122.7 | 152.4 |  |  |  |  |  |  |  |
|  | **30** |  |  |  |  |  |  |  | 68.2 | 74.2 | 85.1 | 95.2 | 108.5 | 137.5 | 171.6 |  |  |  |  |  |  |  |
|  | **40** |  |  |  |  |  |  |  | 72.1 | 78.2 | 89.3 | 99.4 | 112.5 | 140.5 | 172.3 |  |  |  |  |  |  |  |
|  | **50** | 54.8 | 67.4 | 79.3 | 85.8 | 92.0 | 102.2 | 111.7 | 73.7 | 79.8 | 90.7 | 100.6 | 113.4 | 139.9 | 169.3 | 62.0 | 68.5 | 79.0 | 87.4 | 96.7 | 111.9 | 124.1 |
|  | **60** | 48.9 | 63.7 | 77.4 | 84.9 | 92.1 | 103.6 | 114.4 | 74.4 | 80.4 | 91.1 | 100.7 | 113.0 | 138.2 | 165.4 | 61.9 | 68.6 | 79.4 | 87.9 | 97.5 | 113.2 | 125.9 |
|  | **70** | 54.1 | 66.5 | 78.2 | 84.6 | 90.7 | 100.7 | 110.0 | 74.8 | 80.6 | 90.9 | 100.0 | 111.6 | 134.9 | 159.3 | 63.2 | 69.8 | 80.6 | 89.2 | 98.7 | 114.3 | 126.9 |
|  | **80** | 52.8 | 65.5 | 77.4 | 83.9 | 90.1 | 100.3 | 109.8 | 74.3 | 79.9 | 89.8 | 98.5 | 109.4 | 131.0 | 153.3 | 65.1 | 70.8 | 80.0 | 87.0 | 94.8 | 107.3 | 117.1 |
|  | **90** | 53.4 | 65.2 | 76.3 | 82.3 | 88.2 | 97.7 | 106.7 |  |  |  |  |  |  |  |  |  |  |  |  |  |  |
| **Grip strength, kg** | **20** |  |  |  |  |  |  |  | 18.5 | 22.6 | 30.0 | 36.3 | 43.6 | 56.4 | 67.2 |  |  |  |  |  |  |  |
|  | **30** |  |  |  |  |  |  |  | 20.5 | 25.2 | 33.7 | 40.9 | 49.4 | 64.3 | 76.8 |  |  |  |  |  |  |  |
|  | **40** |  |  |  |  |  |  |  | 20.1 | 24.7 | 32.7 | 39.6 | 47.6 | 61.6 | 73.5 |  |  |  |  |  |  |  |
|  | **50** | 11.0 | 17.4 | 26.8 | 33.6 | 40.5 | 50.5 | 57.6 | 18.9 | 23.1 | 30.6 | 37.0 | 44.4 | 57.3 | 68.2 | 13.1 | 18.3 | 27.2 | 34.3 | 42.3 | 55.1 | 65.2 |
|  | **60** | 10.7 | 16.5 | 24.9 | 31.0 | 37.1 | 46.1 | 52.4 | 16.9 | 20.8 | 27.8 | 33.7 | 40.7 | 52.8 | 63.1 | 11.8 | 16.7 | 24.8 | 31.4 | 38.7 | 50.5 | 59.8 |
|  | **70** | 10.4 | 15.3 | 22.6 | 27.8 | 33.0 | 40.7 | 46.2 | 14.3 | 17.9 | 24.4 | 30.1 | 36.8 | 48.7 | 58.9 | 10.9 | 15.5 | 23.3 | 29.5 | 36.6 | 47.9 | 56.9 |
|  | **80** | 7.8 | 11.7 | 17.4 | 21.5 | 25.7 | 31.8 | 36.1 | 11.8 | 15.1 | 21.2 | 26.5 | 33.0 | 44.8 | 55.0 | 10.5 | 14.6 | 21.6 | 27.1 | 33.3 | 43.3 | 51.1 |
|  | **90** | 6.5 | 8.6 | 11.7 | 13.9 | 16.1 | 19.3 | 21.6 |  |  |  |  |  |  |  |  |  |  |  |  |  |  |
| **FEV_1_, ml** | **20** |  |  |  |  |  |  |  | 1947.36 | 2318.75 | 2935.26 | 3430.31 | 3984.86 | 4896.14 | 5622.84 |  |  |  |  |  |  |  |
|  | **30** |  |  |  |  |  |  |  | 1998.55 | 2396.86 | 3062.13 | 3599.24 | 4203.40 | 5200.78 | 5999.47 |  |  |  |  |  |  |  |
|  | **40** |  |  |  |  |  |  |  | 1738.37 | 2110.71 | 2739.19 | 3251.35 | 3831.46 | 4796.65 | 5575.03 |  |  |  |  |  |  |  |
|  | **50** |  |  |  |  |  |  |  | 1474.11 | 1802.26 | 2359.42 | 2815.80 | 3334.73 | 4201.85 | 4903.84 | 1377.15 | 1801.58 | 2462.17 | 2964.40 | 3508.78 | 4384.81 | 5084.08 |
|  | **60** |  |  |  |  |  |  |  | 1238.49 | 1516.26 | 1988.44 | 2375.60 | 2816.17 | 3552.95 | 4149.88 | 1197.65 | 1579.94 | 2177.22 | 2632.57 | 3127.03 | 3924.11 | 4561.32 |
|  | **70** |  |  |  |  |  |  |  | 985.84 | 1228.15 | 1646.19 | 1993.39 | 2392.27 | 3066.44 | 3617.83 | 999.61 | 1350.76 | 1905.38 | 2331.48 | 2796.44 | 3549.66 | 4154.25 |
|  | **80** |  |  |  |  |  |  |  | 740.83 | 957.31 | 1342.48 | 1670.91 | 2055.60 | 2719.77 | 3273.29 | 682.93 | 1009.02 | 1544.58 | 1967.07 | 2435.63 | 3206.74 | 3833.70 |
| **Peak expiratory flow, ml/s** | **20** |  |  |  |  |  |  |  | 4200.6 | 5314.7 | 7010.9 | 8262.1 | 9572.0 | 11561.8 | 13036.9 |  |  |  |  |  |  |  |
|  | **30** |  |  |  |  |  |  |  | 4323.2 | 5592.2 | 7536.6 | 8977.6 | 10490.3 | 12794.5 | 14506.5 |  |  |  |  |  |  |  |
|  | **40** |  |  |  |  |  |  |  | 3676.8 | 4952.2 | 6929.2 | 8405.8 | 9963.4 | 12346.4 | 14123.4 |  |  |  |  |  |  |  |
|  | **50** | 1963.4 | 2772.7 | 4208.8 | 5413.0 | 6793.5 | 9100.4 | 10954.7 | 3209.4 | 4377.5 | 6194.7 | 7555.2 | 8992.2 | 11193.7 | 12836.9 | 1853.0 | 3061.0 | 4962.7 | 6392.1 | 7911.7 | 10277.7 | 12090.9 |
|  | **60** | 1683.1 | 2415.6 | 3727.6 | 4835.2 | 6110.6 | 8251.2 | 9977.8 | 2677.3 | 3743.4 | 5413.3 | 6669.0 | 7998.9 | 10041.1 | 11568.2 | 1399.8 | 2527.5 | 4346.0 | 5727.6 | 7204.0 | 9512.9 | 11287.9 |
|  | **70** | 1301.4 | 1929.7 | 3076.3 | 4057.5 | 5196.9 | 7125.2 | 8690.9 | 1837.7 | 2857.4 | 4500.7 | 5757.8 | 7102.0 | 9183.9 | 10751.0 | 1030.1 | 2054.4 | 3757.5 | 5066.7 | 6473.0 | 8681.8 | 10385.0 |
|  | **80** | 965.8 | 1423.1 | 2254.8 | 2964.7 | 3787.7 | 5178.4 | 6306.2 | 967.1 | 1916.6 | 3557.2 | 4853.9 | 6263.0 | 8475.3 | 10157.0 | 396.3 | 1216.2 | 2863.9 | 4206.6 | 5680.3 | 8031.4 | 9863.7 |
|  | **90** | 685.3 | 984.8 | 1521.6 | 1975.1 | 2497.4 | 3374.4 | 4082.0 |  |  |  |  |  |  |  |  |  |  |  |  |  |  |
|  | **100** | 351.7 | 498.0 | 758.1 | 976.4 | 1226.9 | 1645.8 | 1982.7 |  |  |  |  |  |  |  |  |  |  |  |  |  |  |
| **Fat-free mass index, kg/m^2^** | **20** |  |  |  |  |  |  |  | 12.1 | 13.4 | 15.8 | 17.7 | 20.1 | 24.3 | 28.0 |  |  |  |  |  |  |  |
|  | **30** |  |  |  |  |  |  |  | 13.1 | 14.5 | 17.0 | 19.1 | 21.5 | 25.9 | 29.7 |  |  |  |  |  |  |  |
|  | **40** |  |  |  |  |  |  |  | 13.3 | 14.7 | 17.2 | 19.3 | 21.7 | 26.0 | 29.8 |  |  |  |  |  |  |  |
|  | **50** |  |  |  |  |  |  |  | 13.0 | 14.5 | 16.9 | 18.9 | 21.3 | 25.6 | 29.4 | 13.3 | 14.4 | 16.2 | 17.6 | 19.2 | 22.0 | 24.2 |
|  | **60** |  |  |  |  |  |  |  | 12.5 | 14.0 | 16.4 | 18.5 | 20.9 | 25.4 | 29.3 | 13.1 | 14.1 | 15.8 | 17.1 | 18.6 | 21.2 | 23.2 |
|  | **70** |  |  |  |  |  |  |  |  |  |  |  |  |  |  | 13.1 | 14.1 | 15.7 | 17.0 | 18.5 | 20.9 | 22.8 |
|  | **80** |  |  |  |  |  |  |  |  |  |  |  |  |  |  | 13.7 | 14.5 | 15.8 | 16.8 | 17.9 | 19.7 | 21.1 |
| **Bone mineral content, kg** | **20** |  |  |  |  |  |  |  | 1.39 | 1.60 | 1.95 | 2.24 | 2.56 | 3.12 | 3.58 |  |  |  |  |  |  |  |
|  | **30** |  |  |  |  |  |  |  | 1.54 | 1.74 | 2.08 | 2.35 | 2.66 | 3.18 | 3.60 |  |  |  |  |  |  |  |
|  | **40** |  |  |  |  |  |  |  | 1.50 | 1.69 | 2.02 | 2.28 | 2.57 | 3.06 | 3.46 |  |  |  |  |  |  |  |
|  | **50** |  |  |  |  |  |  |  | 1.38 | 1.58 | 1.92 | 2.20 | 2.52 | 3.06 | 3.51 | 1.78 | 2.02 | 2.41 | 2.72 | 3.07 | 3.65 | 4.12 |
|  | **60** |  |  |  |  |  |  |  | 1.19 | 1.41 | 1.78 | 2.09 | 2.46 | 3.11 | 3.67 | 1.56 | 1.80 | 2.22 | 2.56 | 2.95 | 3.61 | 4.16 |
|  | **70** |  |  |  |  |  |  |  |  |  |  |  |  |  |  | 1.45 | 1.70 | 2.14 | 2.50 | 2.92 | 3.65 | 4.26 |
|  | **80** |  |  |  |  |  |  |  |  |  |  |  |  |  |  | 1.44 | 1.69 | 2.13 | 2.50 | 2.92 | 3.65 | 4.26 |
| **Bone mineral density, g/cm^2^** | **20** |  |  |  |  |  |  |  | 0.86 | 0.93 | 1.03 | 1.10 | 1.17 | 1.29 | 1.38 |  |  |  |  |  |  |  |
|  | **30** |  |  |  |  |  |  |  | 0.90 | 0.97 | 1.06 | 1.13 | 1.20 | 1.31 | 1.39 |  |  |  |  |  |  |  |
|  | **40** |  |  |  |  |  |  |  | 0.89 | 0.96 | 1.05 | 1.11 | 1.18 | 1.28 | 1.36 |  |  |  |  |  |  |  |
|  | **50** |  |  |  |  |  |  |  | 0.85 | 0.91 | 1.01 | 1.09 | 1.17 | 1.29 | 1.37 | 0.98 | 1.06 | 1.18 | 1.26 | 1.34 | 1.47 | 1.55 |
|  | **60** |  |  |  |  |  |  |  | 0.75 | 0.83 | 0.96 | 1.05 | 1.15 | 1.30 | 1.42 | 0.88 | 0.97 | 1.11 | 1.20 | 1.30 | 1.44 | 1.54 |
|  | **70** |  |  |  |  |  |  |  |  |  |  |  |  |  |  | 0.82 | 0.92 | 1.07 | 1.17 | 1.28 | 1.44 | 1.56 |
|  | **80** |  |  |  |  |  |  |  |  |  |  |  |  |  |  | 0.80 | 0.90 | 1.05 | 1.15 | 1.26 | 1.42 | 1.54 |
| **Frailty index_Lab** | **20** |  |  |  |  |  |  |  | 0.00 | 0.00 | 0.04 | 0.13 | 0.24 | 0.39 | 0.48 |  |  |  |  |  |  |  |
|  | **30** |  |  |  |  |  |  |  | 0.00 | 0.00 | 0.04 | 0.13 | 0.25 | 0.41 | 0.50 |  |  |  |  |  |  |  |
|  | **40** |  |  |  |  |  |  |  | 0.00 | 0.01 | 0.06 | 0.17 | 0.30 | 0.47 | 0.56 |  |  |  |  |  |  |  |
|  | **50** | 0 | 0.01 | 0.04 | 0.10 | 0.19 | 0.42 | 0.65 | 0.00 | 0.01 | 0.07 | 0.19 | 0.33 | 0.52 | 0.62 |  |  |  |  |  |  |  |
|  | **60** | 0 | 0.01 | 0.04 | 0.10 | 0.20 | 0.44 | 0.67 | 0.00 | 0.01 | 0.09 | 0.21 | 0.36 | 0.55 | 0.66 |  |  |  |  |  |  |  |
|  | **70** | 0 | 0.01 | 0.05 | 0.11 | 0.22 | 0.48 | 0.73 | 0.00 | 0.02 | 0.11 | 0.25 | 0.40 | 0.59 | 0.69 |  |  |  |  |  |  |  |
|  | **80** | 0 | 0.01 | 0.05 | 0.12 | 0.23 | 0.50 | 0.77 | 0.00 | 0.02 | 0.12 | 0.26 | 0.41 | 0.60 | 0.71 |  |  |  |  |  |  |  |
|  | **90** | 0 | 0.01 | 0.05 | 0.13 | 0.26 | 0.56 | 0.86 |  |  |  |  |  |  |  |  |  |  |  |  |  |  |

**Note:** FEV_1_, forced expiratory volume in the first second; CHARLS, the China Health and Retirement Longitudinal Study; NHANES, the National Health and Nutrition Examination Survey; UKB, the UK Biobank.

**Table S10** **Age-specific percentile values of multi-systemic aging metrics for participants in the “high-income” group in three datasets**

|  |  | **CHARLS** | | | | | | | **NHANES** | | | | | | | **UKB** | | | | | | |
| --- | --- | --- | --- | --- | --- | --- | --- | --- | --- | --- | --- | --- | --- | --- | --- | --- | --- | --- | --- | --- | --- | --- |
|  | **Age,**  **years** | **1st** | **5th** | **25th** | **50th** | **75th** | **95th** | **99th** | **1st** | **5th** | **25th** | **50th** | **75th** | **95th** | **99th** | **1st** | **5th** | **25th** | **50th** | **75th** | **95th** | **99th** |
| **Cognitive function score** | **50** | 4.3 | 7.5 | 11.5 | 13.7 | 15.6 | 17.8 | 19.2 |  |  |  |  |  |  |  | 2.2 | 3.5 | 5.4 | 6.8 | 8.3 | 10.4 | 12.0 |
|  | **60** | 3.1 | 5.8 | 9.9 | 12.3 | 14.3 | 16.8 | 18.2 | 6.9 | 9.6 | 14.2 | 17.7 | 21.5 | 27.5 | 32.1 | 2.3 | 3.5 | 5.4 | 6.7 | 8.1 | 10.2 | 11.6 |
|  | **70** | 2.5 | 4.8 | 8.9 | 11.6 | 13.9 | 16.6 | 18.2 | 6.0 | 8.6 | 13.0 | 16.4 | 20.0 | 25.8 | 30.3 | 2.1 | 3.3 | 5.1 | 6.5 | 7.9 | 10.0 | 11.4 |
|  | **80** | 2.2 | 4.3 | 8.1 | 10.8 | 13.1 | 15.9 | 17.5 | 4.8 | 7.1 | 10.9 | 14.0 | 17.3 | 22.6 | 26.6 | 1.6 | 2.7 | 4.4 | 5.7 | 7.0 | 8.9 | 10.2 |
|  | **90** | 1.9 | 3.8 | 7.3 | 9.8 | 12.0 | 14.8 | 16.4 |  |  |  |  |  |  |  |  |  |  |  |  |  |  |
| **Depression score** | **20** |  |  |  |  |  |  |  | 0.0 | 0.2 | 0.9 | 2.2 | 4.4 | 9.6 | 14.7 |  |  |  |  |  |  |  |
|  | **30** |  |  |  |  |  |  |  | 0.0 | 0.1 | 0.8 | 2.0 | 3.9 | 8.5 | 13.0 |  |  |  |  |  |  |  |
|  | **40** |  |  |  |  |  |  |  | 0.0 | 0.1 | 0.8 | 1.9 | 3.8 | 8.3 | 12.7 |  |  |  |  |  |  |  |
|  | **50** | 0.1 | 0.3 | 1.6 | 3.9 | 7.7 | 16.6 | 25.6 | 0.0 | 0.1 | 0.8 | 2.0 | 4.0 | 8.6 | 13.3 |  |  |  |  |  |  |  |
|  | **60** | 0.1 | 0.3 | 1.7 | 4.1 | 8.2 | 17.6 | 27.1 | 0.0 | 0.2 | 0.9 | 2.0 | 4.1 | 8.9 | 13.6 |  |  |  |  |  |  |  |
|  | **70** | 0.1 | 0.3 | 1.8 | 4.3 | 8.7 | 18.7 | 28.8 | 0.0 | 0.1 | 0.8 | 2.0 | 3.9 | 8.5 | 13.1 |  |  |  |  |  |  |  |
|  | **80** | 0.1 | 0.3 | 1.7 | 4.2 | 8.3 | 18.0 | 27.7 | 0.0 | 0.1 | 0.8 | 2.0 | 3.9 | 8.5 | 13.1 |  |  |  |  |  |  |  |
|  | **90** | 0.1 | 0.3 | 1.7 | 4.1 | 8.2 | 17.7 | 27.3 |  |  |  |  |  |  |  |  |  |  |  |  |  |  |
|  | **100** |  |  |  |  |  |  |  |  |  |  |  |  |  |  |  |  |  |  |  |  |  |
| **Systolic blood pressure, mmHg** | **20** |  |  |  |  |  |  |  | 89.7 | 95.7 | 104.4 | 110.9 | 118.1 | 130.3 | 141.1 |  |  |  |  |  |  |  |
|  | **30** |  |  |  |  |  |  |  | 90.6 | 97.3 | 107.3 | 114.7 | 123.1 | 137.5 | 150.6 |  |  |  |  |  |  |  |
|  | **40** |  |  |  |  |  |  |  | 91.2 | 98.5 | 109.5 | 117.8 | 127.2 | 143.6 | 158.7 |  |  |  |  |  |  |  |
|  | **50** | 92.0 | 99.4 | 111.8 | 122.2 | 134.3 | 156.0 | 175.1 | 92.7 | 100.7 | 112.8 | 122.0 | 132.5 | 151.2 | 168.6 | 96.6 | 105.0 | 117.7 | 127.4 | 137.8 | 154.8 | 168.3 |
|  | **60** | 95.1 | 102.7 | 115.6 | 126.3 | 138.9 | 161.2 | 181.0 | 94.7 | 103.4 | 116.7 | 127.0 | 138.7 | 159.9 | 179.9 | 100.6 | 109.7 | 123.7 | 134.3 | 145.9 | 164.7 | 179.8 |
|  | **70** | 97.6 | 105.5 | 119.0 | 130.2 | 143.4 | 166.9 | 187.8 | 96.1 | 105.6 | 120.3 | 131.8 | 145.1 | 169.4 | 192.9 | 106.1 | 115.9 | 130.9 | 142.4 | 154.9 | 175.2 | 191.7 |
|  | **80** | 100.7 | 109.6 | 125.1 | 138.2 | 153.9 | 182.8 | 209.2 | 96.3 | 106.8 | 123.3 | 136.5 | 152.0 | 181.2 | 210.1 | 109.0 | 119.6 | 136.2 | 148.8 | 162.7 | 185.4 | 203.8 |
|  | **90** | 105.8 | 116.0 | 133.8 | 149.3 | 168.1 | 203.5 | 237.0 |  |  |  |  |  |  |  |  |  |  |  |  |  |  |
| **Diastolic blood pressure, mmHg** | **20** |  |  |  |  |  |  |  | 40.8 | 48.9 | 58.6 | 64.8 | 71.1 | 81.1 | 89.8 |  |  |  |  |  |  |  |
|  | **30** |  |  |  |  |  |  |  | 46.0 | 54.3 | 64.3 | 70.7 | 77.2 | 87.6 | 96.5 |  |  |  |  |  |  |  |
|  | **40** |  |  |  |  |  |  |  | 48.7 | 57.4 | 67.8 | 74.5 | 81.3 | 92.1 | 101.4 |  |  |  |  |  |  |  |
|  | **50** | 53.3 | 59.0 | 68.4 | 75.8 | 84.1 | 97.9 | 109.2 | 49.5 | 58.3 | 68.9 | 75.7 | 82.6 | 93.5 | 103.0 | 56.6 | 62.6 | 71.7 | 78.3 | 85.3 | 96.0 | 104.0 |
|  | **60** | 54.7 | 60.2 | 69.0 | 75.9 | 83.5 | 96.1 | 106.3 | 47.1 | 56.1 | 67.0 | 74.0 | 81.1 | 92.4 | 102.1 | 57.9 | 63.8 | 72.7 | 79.2 | 86.0 | 96.5 | 104.2 |
|  | **70** | 54.5 | 59.4 | 67.2 | 73.3 | 79.9 | 90.8 | 99.4 | 42.1 | 51.5 | 62.9 | 70.2 | 77.6 | 89.4 | 99.6 | 57.0 | 62.9 | 71.8 | 78.3 | 85.1 | 95.5 | 103.3 |
|  | **80** | 50.7 | 55.8 | 63.9 | 70.3 | 77.4 | 89.0 | 98.5 | 36.5 | 46.2 | 58.0 | 65.5 | 73.2 | 85.5 | 96.2 | 54.0 | 60.3 | 69.7 | 76.6 | 83.9 | 95.1 | 103.5 |
|  | **90** | 47.9 | 53.2 | 61.8 | 68.5 | 76.2 | 88.8 | 99.2 |  |  |  |  |  |  |  |  |  |  |  |  |  |  |
|  | **100** |  |  |  |  |  |  |  |  |  |  |  |  |  |  |  |  |  |  |  |  |  |
| **Pulse** | **20** |  |  |  |  |  |  |  | 51.3 | 57.5 | 66.9 | 74.1 | 82.0 | 95.4 | 107.1 |  |  |  |  |  |  |  |
|  | **30** |  |  |  |  |  |  |  | 49.8 | 55.9 | 65.1 | 72.0 | 79.7 | 92.9 | 104.3 |  |  |  |  |  |  |  |
|  | **40** |  |  |  |  |  |  |  | 49.3 | 55.3 | 64.5 | 71.4 | 79.1 | 92.3 | 103.7 |  |  |  |  |  |  |  |
|  | **50** | 53.0 | 58.9 | 67.4 | 73.6 | 80.5 | 92.0 | 102.1 | 48.3 | 54.4 | 63.6 | 70.6 | 78.4 | 91.8 | 103.4 | 47.7 | 53.6 | 63.0 | 70.5 | 78.9 | 93.4 | 105.8 |
|  | **60** | 51.9 | 57.8 | 66.5 | 72.8 | 79.8 | 91.6 | 101.9 | 47.0 | 53.1 | 62.4 | 69.5 | 77.4 | 90.9 | 102.8 | 46.5 | 52.2 | 61.5 | 68.7 | 77.0 | 91.2 | 103.3 |
|  | **70** | 50.7 | 56.5 | 64.9 | 71.1 | 77.9 | 89.4 | 99.5 | 46.2 | 52.2 | 61.4 | 68.4 | 76.2 | 89.6 | 101.3 | 46.2 | 52.0 | 61.3 | 68.6 | 77.0 | 91.3 | 103.6 |
|  | **80** | 48.8 | 55.0 | 64.1 | 70.9 | 78.5 | 91.4 | 102.9 | 45.6 | 51.5 | 60.5 | 67.3 | 75.0 | 88.1 | 99.6 | 45.4 | 51.0 | 59.9 | 66.9 | 74.9 | 88.6 | 100.3 |
|  | **90** | 48.7 | 55.2 | 64.9 | 72.1 | 80.2 | 94.1 | 106.6 |  |  |  |  |  |  |  |  |  |  |  |  |  |  |
| **Body mass index, kg/m^2^** | **20** |  |  |  |  |  |  |  | 15.8 | 17.6 | 21.0 | 24.2 | 28.5 | 38.0 | 49.4 |  |  |  |  |  |  |  |
|  | **30** |  |  |  |  |  |  |  | 17.5 | 19.6 | 23.5 | 27.2 | 32.3 | 43.9 | 58.2 |  |  |  |  |  |  |  |
|  | **40** |  |  |  |  |  |  |  | 18.6 | 20.7 | 24.7 | 28.6 | 33.7 | 45.3 | 59.2 |  |  |  |  |  |  |  |
|  | **50** | 17.7 | 19.4 | 22.1 | 24.2 | 26.5 | 30.3 | 33.3 | 19.0 | 21.2 | 25.2 | 28.9 | 33.9 | 44.9 | 57.8 | 18.4 | 20.3 | 23.4 | 25.9 | 29.0 | 35.1 | 41.3 |
|  | **60** | 17.1 | 18.8 | 21.5 | 23.6 | 26.0 | 29.8 | 33.0 | 19.1 | 21.2 | 25.1 | 28.7 | 33.5 | 43.8 | 55.5 | 18.6 | 20.5 | 23.5 | 26.0 | 29.0 | 34.7 | 40.6 |
|  | **70** | 16.8 | 18.4 | 21.0 | 23.1 | 25.3 | 29.0 | 31.9 | 19.1 | 21.2 | 24.8 | 28.2 | 32.5 | 41.6 | 51.6 | 18.9 | 20.7 | 23.6 | 25.9 | 28.7 | 34.0 | 39.2 |
|  | **80** | 16.2 | 17.7 | 20.1 | 22.0 | 24.0 | 27.4 | 30.1 | 19.0 | 20.8 | 24.2 | 27.2 | 31.1 | 38.9 | 47.2 | 19.0 | 20.6 | 23.2 | 25.4 | 27.9 | 32.5 | 36.9 |
|  | **90** | 15.8 | 17.1 | 19.2 | 20.8 | 22.5 | 25.2 | 27.4 |  |  |  |  |  |  |  |  |  |  |  |  |  |  |
| **Waist circumference, cm** | **20** |  |  |  |  |  |  |  | 61.4 | 66.6 | 76.0 | 84.4 | 95.1 | 117.0 | 140.5 |  |  |  |  |  |  |  |
|  | **30** |  |  |  |  |  |  |  | 67.4 | 73.3 | 83.9 | 93.6 | 106.0 | 131.9 | 160.3 |  |  |  |  |  |  |  |
|  | **40** |  |  |  |  |  |  |  | 70.8 | 76.8 | 87.7 | 97.5 | 109.9 | 135.5 | 163.0 |  |  |  |  |  |  |  |
|  | **50** | 57.0 | 68.7 | 80.6 | 87.4 | 93.9 | 103.8 | 112.1 | 73.0 | 79.0 | 89.7 | 99.2 | 111.2 | 135.3 | 160.5 | 60.8 | 67.1 | 77.4 | 85.4 | 94.2 | 108.3 | 119.5 |
|  | **60** | 57.2 | 68.8 | 80.6 | 87.3 | 93.8 | 103.6 | 111.8 | 74.6 | 80.5 | 91.0 | 100.1 | 111.6 | 134.2 | 157.1 | 61.7 | 68.1 | 78.5 | 86.6 | 95.5 | 109.8 | 121.1 |
|  | **70** | 58.1 | 69.2 | 80.5 | 87.0 | 93.3 | 102.8 | 110.7 | 76.0 | 81.7 | 91.7 | 100.3 | 111.0 | 131.5 | 151.8 | 63.8 | 70.2 | 80.4 | 88.3 | 96.9 | 110.8 | 121.7 |
|  | **80** | 53.1 | 65.7 | 78.5 | 85.8 | 92.7 | 103.2 | 111.9 | 76.5 | 82.0 | 91.5 | 99.6 | 109.5 | 128.1 | 145.9 | 65.0 | 71.4 | 81.5 | 89.3 | 97.8 | 111.5 | 122.2 |
|  | **90** | 49.9 | 63.0 | 76.2 | 83.7 | 90.8 | 101.5 | 110.4 |  |  |  |  |  |  |  |  |  |  |  |  |  |  |
| **Grip strength, kg** | **20** |  |  |  |  |  |  |  | 17.7 | 22.1 | 29.8 | 36.3 | 43.7 | 56.5 | 67.1 |  |  |  |  |  |  |  |
|  | **30** |  |  |  |  |  |  |  | 19.7 | 24.9 | 34.0 | 41.7 | 50.7 | 66.2 | 79.2 |  |  |  |  |  |  |  |
|  | **40** |  |  |  |  |  |  |  | 20.0 | 25.0 | 33.7 | 41.0 | 49.5 | 64.1 | 76.1 |  |  |  |  |  |  |  |
|  | **50** | 15.2 | 21.4 | 30.7 | 37.4 | 44.1 | 54.1 | 61.2 | 19.2 | 23.8 | 31.9 | 38.6 | 46.4 | 59.7 | 70.7 | 14.0 | 19.1 | 27.9 | 35.0 | 42.9 | 55.7 | 65.7 |
|  | **60** | 15.8 | 21.6 | 30.0 | 36.0 | 42.2 | 51.2 | 57.6 | 17.6 | 22.0 | 29.5 | 35.9 | 43.2 | 55.8 | 66.1 | 12.0 | 16.9 | 25.2 | 32.0 | 39.6 | 52.0 | 61.8 |
|  | **70** | 13.5 | 18.7 | 26.4 | 31.9 | 37.5 | 45.7 | 51.6 | 15.5 | 19.4 | 26.4 | 32.3 | 39.2 | 51.0 | 60.9 | 11.0 | 15.6 | 23.6 | 30.2 | 37.6 | 49.7 | 59.2 |
|  | **80** | 9.1 | 14.5 | 22.5 | 28.3 | 34.3 | 43.0 | 49.3 | 12.7 | 16.2 | 22.5 | 27.9 | 34.3 | 45.3 | 54.5 | 9.9 | 14.1 | 21.5 | 27.5 | 34.3 | 45.5 | 54.2 |
|  | **90** | 4.5 | 9.9 | 18.6 | 25.0 | 31.5 | 41.3 | 48.3 |  |  |  |  |  |  |  |  |  |  |  |  |  |  |
| **FEV_1_, ml** | **20** |  |  |  |  |  |  |  | 1950.19 | 2333.23 | 2963.69 | 3465.16 | 4022.14 | 4927.25 | 5640.73 |  |  |  |  |  |  |  |
|  | **30** |  |  |  |  |  |  |  | 2083.05 | 2490.42 | 3160.54 | 3693.31 | 4284.84 | 5245.71 | 6002.88 |  |  |  |  |  |  |  |
|  | **40** |  |  |  |  |  |  |  | 1872.64 | 2246.93 | 2864.39 | 3356.49 | 3903.85 | 4794.76 | 5498.05 |  |  |  |  |  |  |  |
|  | **50** |  |  |  |  |  |  |  | 1573.46 | 1911.84 | 2475.55 | 2928.63 | 3435.75 | 4266.83 | 4926.91 | 1462.59 | 1885.97 | 2549.86 | 3051.68 | 3585.87 | 4411.21 | 5031.65 |
|  | **60** |  |  |  |  |  |  |  | 1307.88 | 1620.55 | 2149.40 | 2580.01 | 3066.56 | 3872.21 | 4517.97 | 1248.16 | 1641.30 | 2262.20 | 2733.93 | 3237.71 | 4018.48 | 4606.89 |
|  | **70** |  |  |  |  |  |  |  | 1091.77 | 1378.49 | 1870.55 | 2276.14 | 2738.48 | 3511.43 | 4136.19 | 1010.95 | 1378.72 | 1967.22 | 2418.42 | 2902.96 | 3657.92 | 4229.29 |
|  | **80** |  |  |  |  |  |  |  | 925.13 | 1179.87 | 1620.41 | 1985.87 | 2404.40 | 3107.58 | 3678.40 | 774.74 | 1109.95 | 1655.68 | 2078.90 | 2536.51 | 3254.05 | 3799.81 |
| **Peak expiratory flow, ml/s** | **20** |  |  |  |  |  |  |  | 4321.0 | 5397.9 | 7078.7 | 8343.4 | 9683.1 | 11736.7 | 13264.3 |  |  |  |  |  |  |  |
|  | **30** |  |  |  |  |  |  |  | 4789.4 | 5983.9 | 7848.2 | 9251.0 | 10737.1 | 13015.0 | 14709.6 |  |  |  |  |  |  |  |
|  | **40** |  |  |  |  |  |  |  | 4506.3 | 5674.2 | 7502.3 | 8880.8 | 10343.3 | 12588.2 | 14260.1 |  |  |  |  |  |  |  |
|  | **50** | 2107.5 | 3091.6 | 4722.9 | 6003.1 | 7395.1 | 9582.5 | 11242.5 | 3876.1 | 5010.5 | 6803.7 | 8165.6 | 9617.3 | 11855.4 | 13528.3 | 1801.2 | 3065.5 | 5087.3 | 6610.4 | 8213.7 | 10642.5 | 12424.7 |
|  | **60** | 1853.0 | 2789.3 | 4355.6 | 5591.7 | 6940.0 | 9065.0 | 10681.2 | 3154.3 | 4265.9 | 6052.6 | 7425.4 | 8899.2 | 11187.3 | 12907.1 | 1518.2 | 2682.3 | 4560.4 | 5980.4 | 7477.8 | 9749.3 | 11417.7 |
|  | **70** | 1420.7 | 2243.4 | 3642.3 | 4757.2 | 5980.1 | 7917.1 | 9395.8 | 2551.9 | 3611.9 | 5343.1 | 6687.9 | 8141.0 | 10410.7 | 12124.8 | 917.0 | 1990.2 | 3831.1 | 5252.2 | 6763.7 | 9071.7 | 10774.6 |
|  | **80** | 791.9 | 1447.7 | 2618.6 | 3577.2 | 4643.7 | 6353.8 | 7671.3 | 2098.7 | 3075.2 | 4689.5 | 5953.2 | 7325.0 | 9476.5 | 11106.6 | 611.0 | 1533.9 | 3211.6 | 4528.6 | 5937.6 | 8098.2 | 9696.7 |
|  | **90** | 396.8 | 846.7 | 1694.3 | 2405.6 | 3206.7 | 4504.3 | 5511.0 |  |  |  |  |  |  |  |  |  |  |  |  |  |  |
|  | **100** | 142.3 | 370.6 | 835.7 | 1238.0 | 1697.3 | 2449.2 | 3036.9 |  |  |  |  |  |  |  |  |  |  |  |  |  |  |
| **Fat-free mass index, kg/m^2^** | **20** |  |  |  |  |  |  |  | 12.0 | 13.3 | 15.7 | 17.6 | 19.9 | 24.0 | 27.6 |  |  |  |  |  |  |  |
|  | **30** |  |  |  |  |  |  |  | 12.6 | 14.1 | 16.6 | 18.8 | 21.4 | 26.0 | 30.1 |  |  |  |  |  |  |  |
|  | **40** |  |  |  |  |  |  |  | 12.9 | 14.3 | 16.9 | 19.0 | 21.5 | 26.0 | 30.0 |  |  |  |  |  |  |  |
|  | **50** |  |  |  |  |  |  |  | 13.0 | 14.5 | 16.9 | 18.9 | 21.3 | 25.6 | 29.3 | 12.6 | 13.9 | 15.9 | 17.4 | 19.0 | 21.7 | 23.9 |
|  | **60** |  |  |  |  |  |  |  | 12.8 | 14.2 | 16.6 | 18.6 | 21.0 | 25.2 | 28.8 | 12.4 | 13.7 | 15.6 | 17.1 | 18.7 | 21.3 | 23.5 |
|  | **70** |  |  |  |  |  |  |  |  |  |  |  |  |  |  | 12.7 | 14.0 | 15.8 | 17.2 | 18.6 | 21.1 | 23.0 |
|  | **80** |  |  |  |  |  |  |  |  |  |  |  |  |  |  | 13.2 | 14.3 | 16.0 | 17.3 | 18.6 | 20.8 | 22.6 |
| **Bone mineral content, kg** | **20** |  |  |  |  |  |  |  | 1.40 | 1.61 | 1.97 | 2.26 | 2.59 | 3.14 | 3.60 |  |  |  |  |  |  |  |
|  | **30** |  |  |  |  |  |  |  | 1.54 | 1.75 | 2.12 | 2.41 | 2.74 | 3.30 | 3.75 |  |  |  |  |  |  |  |
|  | **40** |  |  |  |  |  |  |  | 1.54 | 1.75 | 2.09 | 2.36 | 2.67 | 3.18 | 3.59 |  |  |  |  |  |  |  |
|  | **50** |  |  |  |  |  |  |  | 1.45 | 1.66 | 2.01 | 2.29 | 2.60 | 3.13 | 3.56 | 1.72 | 1.99 | 2.41 | 2.74 | 3.10 | 3.66 | 4.10 |
|  | **60** |  |  |  |  |  |  |  | 1.28 | 1.50 | 1.86 | 2.16 | 2.51 | 3.11 | 3.60 | 1.47 | 1.75 | 2.21 | 2.57 | 2.97 | 3.61 | 4.12 |
|  | **70** |  |  |  |  |  |  |  |  |  |  |  |  |  |  | 1.41 | 1.70 | 2.19 | 2.57 | 3.00 | 3.69 | 4.24 |
|  | **80** |  |  |  |  |  |  |  |  |  |  |  |  |  |  | 1.39 | 1.69 | 2.19 | 2.59 | 3.03 | 3.75 | 4.33 |
| **Bone mineral density, g/cm^2^** | **20** |  |  |  |  |  |  |  | 0.86 | 0.93 | 1.02 | 1.09 | 1.17 | 1.29 | 1.38 |  |  |  |  |  |  |  |
|  | **30** |  |  |  |  |  |  |  | 0.90 | 0.97 | 1.06 | 1.13 | 1.21 | 1.32 | 1.41 |  |  |  |  |  |  |  |
|  | **40** |  |  |  |  |  |  |  | 0.90 | 0.96 | 1.06 | 1.13 | 1.20 | 1.31 | 1.40 |  |  |  |  |  |  |  |
|  | **50** |  |  |  |  |  |  |  | 0.87 | 0.93 | 1.03 | 1.10 | 1.18 | 1.30 | 1.40 | 0.99 | 1.07 | 1.18 | 1.26 | 1.34 | 1.46 | 1.55 |
|  | **60** |  |  |  |  |  |  |  | 0.81 | 0.88 | 0.98 | 1.06 | 1.14 | 1.27 | 1.37 | 0.87 | 0.96 | 1.10 | 1.20 | 1.30 | 1.44 | 1.55 |
|  | **70** |  |  |  |  |  |  |  |  |  |  |  |  |  |  | 0.82 | 0.93 | 1.08 | 1.18 | 1.29 | 1.45 | 1.57 |
|  | **80** |  |  |  |  |  |  |  |  |  |  |  |  |  |  | 0.79 | 0.90 | 1.06 | 1.18 | 1.29 | 1.46 | 1.59 |
| **Frailty index_Lab** | **20** |  |  |  |  |  |  |  | 0.00 | 0.00 | 0.03 | 0.11 | 0.23 | 0.38 | 0.47 |  |  |  |  |  |  |  |
|  | **30** |  |  |  |  |  |  |  | 0.00 | 0.00 | 0.02 | 0.09 | 0.22 | 0.39 | 0.49 |  |  |  |  |  |  |  |
|  | **40** |  |  |  |  |  |  |  | 0.00 | 0.00 | 0.03 | 0.10 | 0.24 | 0.42 | 0.53 |  |  |  |  |  |  |  |
|  | **50** | 0 | 0.01 | 0.04 | 0.09 | 0.19 | 0.41 | 0.63 | 0.00 | 0.00 | 0.03 | 0.12 | 0.26 | 0.46 | 0.57 |  |  |  |  |  |  |  |
|  | **60** | 0 | 0.01 | 0.05 | 0.11 | 0.22 | 0.47 | 0.73 | 0.00 | 0.00 | 0.04 | 0.14 | 0.29 | 0.50 | 0.62 |  |  |  |  |  |  |  |
|  | **70** | 0 | 0.01 | 0.05 | 0.12 | 0.24 | 0.51 | 0.79 | 0.00 | 0.00 | 0.06 | 0.17 | 0.34 | 0.55 | 0.68 |  |  |  |  |  |  |  |
|  | **80** | 0 | 0.01 | 0.05 | 0.12 | 0.24 | 0.51 | 0.79 | 0.00 | 0.01 | 0.09 | 0.22 | 0.38 | 0.59 | 0.71 |  |  |  |  |  |  |  |
|  | **90** | 0 | 0.01 | 0.04 | 0.10 | 0.20 | 0.43 | 0.66 |  |  |  |  |  |  |  |  |  |  |  |  |  |  |
|  | **100** |  |  |  |  |  |  |  |  |  |  |  |  |  |  |  |  |  |  |  |  |  |

**Note:** FEV_1_, forced expiratory volume in the first second; CHARLS, the China Health and Retirement Longitudinal Study; NHANES, the National Health and Nutrition Examination Survey; UKB, the UK Biobank.

**Table S11** **Percentile values of multi-systemic aging metrics for participants in the “low-education” group in three datasets**

|  |  | **CHARLS** | | | | | | | **NHANES** | | | | | | | **UKB** | | | | | | |
| --- | --- | --- | --- | --- | --- | --- | --- | --- | --- | --- | --- | --- | --- | --- | --- | --- | --- | --- | --- | --- | --- | --- |
|  | **Age,**  **years** | **1st** | **5th** | **25th** | **50th** | **75th** | **95th** | **99th** | **1st** | **5th** | **25th** | **50th** | **75th** | **95th** | **99th** | **1st** | **5th** | **25th** | **50th** | **75th** | **95th** | **99th** |
| **Cognitive function score** | **50** | 1.2 | 2.9 | 7.1 | 10.4 | 13.3 | 16.3 | 17.8 |  |  |  |  |  |  |  | 1.4 | 2.4 | 4.1 | 5.3 | 6.6 | 8.7 | 10.2 |
|  | **60** | 0.5 | 1.7 | 5.4 | 8.9 | 12.1 | 15.4 | 17.1 | 6.4 | 8.8 | 12.1 | 14.4 | 16.9 | 21.0 | 24.7 | 1.5 | 2.6 | 4.2 | 5.4 | 6.8 | 8.8 | 10.3 |
|  | **70** | 0.2 | 0.9 | 4.0 | 7.7 | 11.3 | 15.0 | 16.7 | 5.4 | 7.8 | 11.2 | 13.6 | 16.1 | 20.5 | 24.3 | 1.4 | 2.4 | 4.1 | 5.3 | 6.6 | 8.6 | 10.1 |
|  | **80** | 0.0 | 0.1 | 1.6 | 4.6 | 8.7 | 13.1 | 15.0 | 3.1 | 5.4 | 8.8 | 11.4 | 14.2 | 18.9 | 23.3 | 1.0 | 2.0 | 3.5 | 4.7 | 5.9 | 7.9 | 9.3 |
|  | **90** | 0.0 | 0.0 | 0.3 | 2.0 | 5.7 | 10.9 | 13.0 |  |  |  |  |  |  |  |  |  |  |  |  |  |  |
|  | **100** | 0.0 | 0.0 | 0.0 | 0.5 | 3.1 | 8.8 | 11.2 |  |  |  |  |  |  |  |  |  |  |  |  |  |  |
| **Depression score** | **20** |  |  |  |  |  |  |  | 0.0 | 0.2 | 1.2 | 2.9 | 5.7 | 12.4 | 19.1 |  |  |  |  |  |  |  |
|  | **30** |  |  |  |  |  |  |  | 0.0 | 0.2 | 1.0 | 2.3 | 4.7 | 10.1 | 15.5 |  |  |  |  |  |  |  |
|  | **40** |  |  |  |  |  |  |  | 0.0 | 0.2 | 1.0 | 2.4 | 4.8 | 10.5 | 16.1 |  |  |  |  |  |  |  |
|  | **50** | 0.0 | 0.0 | 1.1 | 4.6 | 11.0 | 20.4 | 25.8 | 0.0 | 0.2 | 1.1 | 2.6 | 5.3 | 11.4 | 17.5 |  |  |  |  |  |  |  |
|  | **60** | 0.0 | 0.1 | 1.5 | 5.6 | 12.3 | 21.8 | 27.2 | 0.0 | 0.2 | 1.2 | 2.8 | 5.6 | 12.2 | 18.7 |  |  |  |  |  |  |  |
|  | **70** | 0.0 | 0.1 | 1.8 | 6.1 | 12.8 | 22.1 | 27.4 | 0.0 | 0.2 | 1.2 | 2.8 | 5.6 | 12.1 | 18.6 |  |  |  |  |  |  |  |
|  | **80** | 0.0 | 0.1 | 1.4 | 5.4 | 11.8 | 21.0 | 26.1 | 0.0 | 0.2 | 1.2 | 2.8 | 5.5 | 12.0 | 18.4 |  |  |  |  |  |  |  |
|  | **90** | 0.0 | 0.0 | 1.0 | 4.4 | 10.3 | 18.9 | 23.8 |  |  |  |  |  |  |  |  |  |  |  |  |  |  |
|  | **100** | 0.0 | 0.0 | 0.7 | 3.5 | 8.9 | 16.9 | 21.5 |  |  |  |  |  |  |  |  |  |  |  |  |  |  |
| **Systolic blood pressure, mmHg** | **20** |  |  |  |  |  |  |  | 89.2 | 95.5 | 105.0 | 112.2 | 120.4 | 134.8 | 148.2 |  |  |  |  |  |  |  |
|  | **30** |  |  |  |  |  |  |  | 88.9 | 95.8 | 106.1 | 114.0 | 123.1 | 139.3 | 154.7 |  |  |  |  |  |  |  |
|  | **40** |  |  |  |  |  |  |  | 90.2 | 97.5 | 108.5 | 117.0 | 126.9 | 144.7 | 161.7 |  |  |  |  |  |  |  |
|  | **50** | 90.2 | 98.0 | 111.0 | 121.4 | 133.4 | 153.6 | 170.5 | 94.1 | 101.9 | 113.9 | 123.2 | 133.9 | 153.5 | 172.4 | 98.3 | 107.0 | 119.8 | 129.4 | 139.5 | 155.8 | 168.8 |
|  | **60** | 91.8 | 100.4 | 114.6 | 126.2 | 139.5 | 162.4 | 181.8 | 97.7 | 106.2 | 119.3 | 129.6 | 141.5 | 163.6 | 185.3 | 101.5 | 111.2 | 125.6 | 136.4 | 147.9 | 166.5 | 181.4 |
|  | **70** | 94.3 | 103.5 | 119.1 | 132.0 | 146.9 | 172.9 | 195.3 | 99.9 | 109.2 | 123.5 | 134.9 | 148.3 | 173.4 | 198.6 | 106.1 | 116.5 | 132.1 | 143.8 | 156.3 | 176.5 | 192.8 |
|  | **80** | 96.5 | 106.3 | 122.9 | 136.7 | 152.8 | 181.0 | 205.4 | 100.5 | 110.8 | 127.1 | 140.4 | 156.3 | 187.1 | 219.2 | 108.3 | 119.6 | 136.6 | 149.3 | 163.0 | 185.3 | 203.4 |
|  | **90** | 98.1 | 108.6 | 126.5 | 141.4 | 159.1 | 190.3 | 217.8 |  |  |  |  |  |  |  |  |  |  |  |  |  |  |
|  | **100** | 98.4 | 109.6 | 129.0 | 145.5 | 165.1 | 200.5 | 232.1 |  |  |  |  |  |  |  |  |  |  |  |  |  |  |
| **Diastolic blood pressure, mmHg** | **20** |  |  |  |  |  |  |  | 41.6 | 48.6 | 57.0 | 62.4 | 67.9 | 77.5 | 86.5 |  |  |  |  |  |  |  |
|  | **30** |  |  |  |  |  |  |  | 44.3 | 52.4 | 61.9 | 68.0 | 74.4 | 85.3 | 95.8 |  |  |  |  |  |  |  |
|  | **40** |  |  |  |  |  |  |  | 46.8 | 55.5 | 65.7 | 72.4 | 79.2 | 91.0 | 102.3 |  |  |  |  |  |  |  |
|  | **50** | 52.3 | 58.2 | 67.6 | 74.9 | 82.8 | 95.6 | 105.6 | 49.3 | 57.9 | 68.1 | 74.6 | 81.3 | 92.9 | 104.0 | 58.0 | 64.0 | 73.0 | 79.6 | 86.4 | 96.7 | 104.3 |
|  | **60** | 52.8 | 58.6 | 67.8 | 74.9 | 82.6 | 95.0 | 104.7 | 48.0 | 56.5 | 66.6 | 73.1 | 79.9 | 91.5 | 102.5 | 59.4 | 65.3 | 74.2 | 80.6 | 87.3 | 97.4 | 104.8 |
|  | **70** | 51.9 | 57.7 | 66.8 | 73.9 | 81.6 | 93.9 | 103.6 | 40.2 | 49.6 | 60.9 | 68.3 | 76.1 | 89.5 | 102.4 | 57.1 | 63.1 | 72.1 | 78.6 | 85.5 | 95.8 | 103.4 |
|  | **80** | 50.2 | 56.0 | 65.0 | 72.0 | 79.7 | 91.9 | 101.6 | 31.3 | 41.4 | 54.0 | 62.3 | 71.1 | 86.5 | 101.5 | 54.3 | 60.5 | 69.9 | 76.8 | 83.9 | 94.8 | 102.8 |
|  | **90** | 48.0 | 53.9 | 63.3 | 70.6 | 78.6 | 91.6 | 101.9 |  |  |  |  |  |  |  |  |  |  |  |  |  |  |
|  | **100** | 45.5 | 51.6 | 61.5 | 69.2 | 77.7 | 91.7 | 102.8 |  |  |  |  |  |  |  |  |  |  |  |  |  |  |
| **Pulse** | **20** |  |  |  |  |  |  |  | 51.7 | 57.4 | 66.5 | 73.7 | 81.8 | 96.1 | 108.6 |  |  |  |  |  |  |  |
|  | **30** |  |  |  |  |  |  |  | 51.0 | 56.5 | 65.2 | 72.1 | 79.9 | 93.4 | 105.3 |  |  |  |  |  |  |  |
|  | **40** |  |  |  |  |  |  |  | 49.6 | 55.0 | 63.6 | 70.4 | 78.2 | 91.6 | 103.5 |  |  |  |  |  |  |  |
|  | **50** | 53.1 | 58.9 | 67.4 | 73.7 | 80.6 | 92.2 | 102.3 | 48.3 | 53.8 | 62.6 | 69.6 | 77.6 | 91.6 | 104.0 | 48.0 | 54.2 | 64.0 | 71.8 | 80.6 | 95.5 | 108.1 |
|  | **60** | 52.4 | 58.3 | 66.8 | 73.2 | 80.1 | 91.9 | 102.2 | 47.8 | 53.4 | 62.3 | 69.4 | 77.6 | 92.0 | 104.9 | 46.6 | 52.7 | 62.5 | 70.2 | 79.0 | 93.9 | 106.5 |
|  | **70** | 51.0 | 57.2 | 66.4 | 73.3 | 81.0 | 94.0 | 105.4 | 47.6 | 53.0 | 61.8 | 68.9 | 76.9 | 91.1 | 103.7 | 46.5 | 52.4 | 61.9 | 69.4 | 77.8 | 92.1 | 104.2 |
|  | **80** | 51.1 | 57.6 | 67.1 | 74.3 | 82.3 | 95.9 | 108.0 | 45.4 | 51.2 | 60.5 | 68.0 | 76.8 | 92.5 | 106.8 | 44.0 | 49.9 | 59.4 | 67.0 | 75.5 | 90.2 | 102.6 |
|  | **90** | 50.4 | 57.0 | 67.0 | 74.5 | 82.8 | 97.3 | 110.1 |  |  |  |  |  |  |  |  |  |  |  |  |  |  |
|  | **100** | 49.2 | 56.0 | 66.4 | 74.3 | 83.1 | 98.4 | 112.2 |  |  |  |  |  |  |  |  |  |  |  |  |  |  |
| **Body mass index, kg/m^2^** | **20** |  |  |  |  |  |  |  | 17.2 | 19.4 | 23.2 | 26.5 | 30.7 | 39.1 | 48.1 |  |  |  |  |  |  |  |
|  | **30** |  |  |  |  |  |  |  | 18.7 | 21.0 | 24.9 | 28.2 | 32.3 | 40.5 | 49.1 |  |  |  |  |  |  |  |
|  | **40** |  |  |  |  |  |  |  | 19.4 | 21.7 | 25.5 | 28.7 | 32.6 | 40.4 | 48.3 |  |  |  |  |  |  |  |
|  | **50** | 17.2 | 19.0 | 21.8 | 24.1 | 26.6 | 30.9 | 34.5 | 19.8 | 22.0 | 25.7 | 28.9 | 32.8 | 40.3 | 47.9 | 18.7 | 20.9 | 24.4 | 27.4 | 30.9 | 37.8 | 45.0 |
|  | **60** | 16.7 | 18.4 | 21.3 | 23.5 | 26.1 | 30.4 | 34.1 | 19.5 | 21.8 | 25.7 | 29.0 | 33.1 | 41.3 | 49.7 | 19.1 | 21.2 | 24.5 | 27.2 | 30.4 | 36.5 | 42.7 |
|  | **70** | 15.9 | 17.6 | 20.5 | 22.8 | 25.4 | 29.8 | 33.7 | 19.0 | 21.2 | 25.0 | 28.3 | 32.3 | 40.3 | 48.5 | 19.3 | 21.2 | 24.3 | 26.8 | 29.7 | 35.0 | 40.3 |
|  | **80** | 15.1 | 16.8 | 19.5 | 21.8 | 24.3 | 28.7 | 32.4 | 17.8 | 20.0 | 23.7 | 26.9 | 30.8 | 38.8 | 47.1 | 19.2 | 20.9 | 23.7 | 25.9 | 28.4 | 32.9 | 37.3 |
|  | **90** | 14.6 | 16.2 | 18.7 | 20.7 | 22.9 | 26.8 | 30.1 |  |  |  |  |  |  |  |  |  |  |  |  |  |  |
|  | **100** | 14.3 | 15.6 | 17.8 | 19.6 | 21.5 | 24.8 | 27.5 |  |  |  |  |  |  |  |  |  |  |  |  |  |  |
| **Waist circumference, cm** | **20** |  |  |  |  |  |  |  | 61.5 | 68.7 | 80.1 | 89.3 | 100.0 | 119.8 | 138.8 |  |  |  |  |  |  |  |
|  | **30** |  |  |  |  |  |  |  | 67.6 | 74.7 | 85.7 | 94.4 | 104.3 | 122.3 | 139.0 |  |  |  |  |  |  |  |
|  | **40** |  |  |  |  |  |  |  | 71.3 | 78.1 | 88.5 | 96.6 | 105.9 | 122.1 | 136.9 |  |  |  |  |  |  |  |
|  | **50** | 49.0 | 65.4 | 79.3 | 86.3 | 92.7 | 103.3 | 113.5 | 74.0 | 80.7 | 90.8 | 98.7 | 107.5 | 122.9 | 136.8 | 61.7 | 68.8 | 80.1 | 88.9 | 98.4 | 113.5 | 125.3 |
|  | **60** | 47.5 | 64.5 | 78.8 | 86.0 | 92.7 | 103.6 | 114.0 | 75.1 | 81.9 | 92.4 | 100.5 | 109.6 | 125.6 | 140.1 | 63.6 | 70.5 | 81.5 | 90.0 | 99.1 | 113.7 | 124.9 |
|  | **70** | 44.1 | 62.1 | 77.6 | 85.4 | 92.5 | 104.1 | 115.2 | 75.5 | 82.3 | 92.8 | 100.8 | 109.9 | 125.7 | 140.0 | 64.7 | 71.4 | 82.1 | 90.3 | 99.1 | 113.1 | 123.9 |
|  | **80** | 43.1 | 61.2 | 76.8 | 84.5 | 91.7 | 103.3 | 114.4 | 72.6 | 79.6 | 90.3 | 98.7 | 108.1 | 124.9 | 140.2 | 65.2 | 71.8 | 82.1 | 90.0 | 98.6 | 112.0 | 122.3 |
|  | **90** | 45.8 | 62.2 | 76.2 | 83.2 | 89.6 | 100.2 | 110.4 |  |  |  |  |  |  |  |  |  |  |  |  |  |  |
|  | **100** | 49.5 | 63.7 | 75.5 | 81.5 | 87.1 | 96.3 | 105.2 |  |  |  |  |  |  |  |  |  |  |  |  |  |  |
| **Grip strength, kg** | **20** |  |  |  |  |  |  |  | 24.2 | 28.6 | 35.5 | 40.9 | 46.6 | 55.7 | 62.6 |  |  |  |  |  |  |  |
|  | **30** |  |  |  |  |  |  |  | 18.7 | 23.7 | 32.1 | 38.8 | 46.1 | 58.0 | 67.2 |  |  |  |  |  |  |  |
|  | **40** |  |  |  |  |  |  |  | 17.1 | 22.1 | 30.7 | 37.5 | 45.1 | 57.5 | 67.1 |  |  |  |  |  |  |  |
|  | **50** | 12.0 | 17.3 | 25.7 | 32.1 | 39.0 | 49.5 | 57.4 | 17.6 | 22.5 | 30.6 | 37.1 | 44.3 | 55.8 | 64.8 | 12.1 | 17.8 | 27.5 | 35.1 | 43.6 | 56.9 | 67.1 |
|  | **60** | 10.1 | 15.0 | 22.8 | 28.8 | 35.2 | 45.1 | 52.5 | 16.8 | 21.3 | 28.7 | 34.6 | 41.1 | 51.5 | 59.7 | 10.2 | 15.6 | 24.8 | 32.1 | 40.2 | 53.1 | 63.0 |
|  | **70** | 8.2 | 12.7 | 20.1 | 25.7 | 31.8 | 41.2 | 48.2 | 13.2 | 17.4 | 24.6 | 30.4 | 36.8 | 47.4 | 55.7 | 9.8 | 14.8 | 23.2 | 29.9 | 37.2 | 48.9 | 57.8 |
|  | **80** | 5.5 | 9.5 | 16.1 | 21.3 | 26.9 | 35.6 | 42.2 | 10.0 | 13.8 | 20.5 | 26.1 | 32.4 | 42.8 | 51.0 | 8.0 | 12.6 | 20.4 | 26.7 | 33.6 | 44.7 | 53.2 |
|  | **90** | 2.9 | 6.4 | 12.5 | 17.4 | 22.7 | 31.2 | 37.6 |  |  |  |  |  |  |  |  |  |  |  |  |  |  |
|  | **100** | 1.3 | 3.9 | 9.6 | 14.4 | 19.7 | 28.3 | 34.8 |  |  |  |  |  |  |  |  |  |  |  |  |  |  |
| **FEV_1_, ml** | **20** |  |  |  |  |  |  |  | 1917.48 | 2336.53 | 3017.88 | 3552.06 | 4137.46 | 5072.09 | 5795.43 |  |  |  |  |  |  |  |
|  | **30** |  |  |  |  |  |  |  | 1957.49 | 2346.39 | 2972.05 | 3458.29 | 3987.83 | 4827.68 | 5473.92 |  |  |  |  |  |  |  |
|  | **40** |  |  |  |  |  |  |  | 1931.35 | 2292.97 | 2871.16 | 3318.16 | 3803.17 | 4569.31 | 5156.76 |  |  |  |  |  |  |  |
|  | **50** |  |  |  |  |  |  |  | 1692.45 | 2017.07 | 2537.41 | 2940.52 | 3378.57 | 4071.64 | 4603.83 | 1378.50 | 1806.20 | 2460.61 | 2949.03 | 3469.31 | 4285.99 | 4919.43 |
|  | **60** |  |  |  |  |  |  |  | 1348.50 | 1646.72 | 2132.26 | 2513.35 | 2931.32 | 3599.19 | 4116.45 | 1176.26 | 1582.87 | 2210.99 | 2682.89 | 3187.65 | 3983.09 | 4602.06 |
|  | **70** |  |  |  |  |  |  |  | 1132.16 | 1398.12 | 1834.27 | 2178.60 | 2557.82 | 3166.41 | 3639.53 | 965.62 | 1335.99 | 1913.76 | 2350.70 | 2819.92 | 3562.20 | 4141.59 |
|  | **80** |  |  |  |  |  |  |  | 894.37 | 1152.06 | 1585.99 | 1935.96 | 2327.10 | 2964.54 | 3466.59 | 753.25 | 1088.06 | 1618.16 | 2022.95 | 2460.14 | 3155.48 | 3700.56 |
| **Peak expiratory flow, ml/s** | **20** |  |  |  |  |  |  |  | 4789.7 | 5764.9 | 7314.1 | 8502.6 | 9783.1 | 11787.6 | 13310.1 |  |  |  |  |  |  |  |
|  | **30** |  |  |  |  |  |  |  | 4259.6 | 5305.9 | 6999.9 | 8319.6 | 9756.5 | 12030.4 | 13773.9 |  |  |  |  |  |  |  |
|  | **40** |  |  |  |  |  |  |  | 3866.2 | 4951.2 | 6734.5 | 8140.3 | 9683.1 | 12144.5 | 14044.5 |  |  |  |  |  |  |  |
|  | **50** | 1920.8 | 2660.3 | 3977.0 | 5088.1 | 6370.1 | 8531.4 | 10284.7 | 3500.0 | 4526.6 | 6223.0 | 7565.7 | 9043.4 | 11407.3 | 13236.3 | 1843.3 | 3005.6 | 4915.9 | 6392.3 | 7975.3 | 10421.0 | 12247.1 |
|  | **60** | 1695.2 | 2371.1 | 3582.0 | 4608.5 | 5796.7 | 7806.2 | 9440.4 | 2878.8 | 3860.0 | 5512.8 | 6840.2 | 8314.8 | 10696.4 | 12553.3 | 1490.1 | 2594.3 | 4441.5 | 5881.8 | 7433.0 | 9838.6 | 11639.8 |
|  | **70** | 1284.0 | 1893.0 | 3020.8 | 4000.5 | 5152.6 | 7132.0 | 8762.4 | 2343.9 | 3250.9 | 4806.2 | 6071.3 | 7488.6 | 9796.1 | 11607.2 | 973.5 | 1955.6 | 3669.6 | 5030.5 | 6508.7 | 8817.2 | 10554.0 |
|  | **80** | 923.1 | 1408.5 | 2326.7 | 3136.4 | 4097.9 | 5765.5 | 7149.5 | 1729.9 | 2573.5 | 4072.4 | 5322.6 | 6744.9 | 9095.5 | 10962.4 | 717.3 | 1575.4 | 3118.8 | 4358.0 | 5710.5 | 7830.5 | 9429.7 |
|  | **90** | 657.5 | 1042.2 | 1787.1 | 2454.6 | 3255.2 | 4657.6 | 5830.5 |  |  |  |  |  |  |  |  |  |  |  |  |  |  |
|  | **100** | 470.0 | 789.1 | 1428.4 | 2014.7 | 2728.0 | 3994.3 | 5064.7 |  |  |  |  |  |  |  |  |  |  |  |  |  |  |
| **Fat-free mass index, kg/m^2^** | **20** |  |  |  |  |  |  |  | 14.0 | 15.3 | 17.4 | 18.9 | 20.6 | 23.2 | 25.1 |  |  |  |  |  |  |  |
|  | **30** |  |  |  |  |  |  |  | 13.8 | 15.2 | 17.6 | 19.4 | 21.4 | 24.5 | 26.9 |  |  |  |  |  |  |  |
|  | **40** |  |  |  |  |  |  |  | 13.1 | 14.7 | 17.2 | 19.1 | 21.2 | 24.6 | 27.3 |  |  |  |  |  |  |  |
|  | **50** |  |  |  |  |  |  |  | 13.5 | 15.1 | 17.5 | 19.4 | 21.5 | 24.7 | 27.3 | 13.0 | 14.3 | 16.3 | 17.9 | 19.6 | 22.2 | 24.3 |
|  | **60** |  |  |  |  |  |  |  | 12.9 | 14.5 | 16.9 | 18.8 | 20.9 | 24.3 | 26.9 | 12.7 | 14.0 | 15.9 | 17.4 | 19.0 | 21.5 | 23.5 |
|  | **70** |  |  |  |  |  |  |  |  |  |  |  |  |  |  | 12.8 | 14.0 | 15.8 | 17.2 | 18.7 | 21.1 | 22.9 |
|  | **80** |  |  |  |  |  |  |  |  |  |  |  |  |  |  | 13.2 | 14.3 | 16.1 | 17.4 | 18.8 | 21.1 | 22.7 |
| **Bone mineral content, kg** | **20** |  |  |  |  |  |  |  | 1.57 | 1.75 | 2.04 | 2.27 | 2.52 | 2.92 | 3.24 |  |  |  |  |  |  |  |
|  | **30** |  |  |  |  |  |  |  | 1.40 | 1.60 | 1.91 | 2.16 | 2.44 | 2.89 | 3.25 |  |  |  |  |  |  |  |
|  | **40** |  |  |  |  |  |  |  | 1.43 | 1.62 | 1.92 | 2.16 | 2.43 | 2.86 | 3.20 |  |  |  |  |  |  |  |
|  | **50** |  |  |  |  |  |  |  | 1.34 | 1.54 | 1.87 | 2.14 | 2.44 | 2.93 | 3.33 | 1.75 | 2.02 | 2.43 | 2.76 | 3.11 | 3.67 | 4.11 |
|  | **60** |  |  |  |  |  |  |  | 1.11 | 1.32 | 1.67 | 1.95 | 2.29 | 2.85 | 3.32 | 1.50 | 1.78 | 2.24 | 2.61 | 3.01 | 3.67 | 4.19 |
|  | **70** |  |  |  |  |  |  |  |  |  |  |  |  |  |  | 1.40 | 1.69 | 2.17 | 2.55 | 2.98 | 3.69 | 4.25 |
|  | **80** |  |  |  |  |  |  |  |  |  |  |  |  |  |  | 1.42 | 1.71 | 2.18 | 2.56 | 2.99 | 3.68 | 4.23 |
| **Bone mineral density, g/cm^2^** | **20** |  |  |  |  |  |  |  | 0.92 | 0.98 | 1.05 | 1.10 | 1.15 | 1.23 | 1.29 |  |  |  |  |  |  |  |
|  | **30** |  |  |  |  |  |  |  | 0.88 | 0.94 | 1.03 | 1.08 | 1.14 | 1.24 | 1.31 |  |  |  |  |  |  |  |
|  | **40** |  |  |  |  |  |  |  | 0.87 | 0.94 | 1.03 | 1.09 | 1.16 | 1.26 | 1.34 |  |  |  |  |  |  |  |
|  | **50** |  |  |  |  |  |  |  | 0.82 | 0.90 | 1.00 | 1.07 | 1.15 | 1.27 | 1.36 | 0.98 | 1.06 | 1.18 | 1.26 | 1.35 | 1.47 | 1.56 |
|  | **60** |  |  |  |  |  |  |  | 0.75 | 0.83 | 0.94 | 1.01 | 1.10 | 1.23 | 1.33 | 0.87 | 0.97 | 1.11 | 1.21 | 1.31 | 1.45 | 1.55 |
|  | **70** |  |  |  |  |  |  |  |  |  |  |  |  |  |  | 0.82 | 0.92 | 1.08 | 1.18 | 1.29 | 1.45 | 1.56 |
|  | **80** |  |  |  |  |  |  |  |  |  |  |  |  |  |  | 0.79 | 0.90 | 1.06 | 1.17 | 1.28 | 1.45 | 1.56 |
| **Frailty index_Lab** | **20** |  |  |  |  |  |  |  | 0.00 | 0.01 | 0.07 | 0.15 | 0.24 | 0.36 | 0.43 |  |  |  |  |  |  |  |
|  | **30** |  |  |  |  |  |  |  | 0.00 | 0.01 | 0.06 | 0.15 | 0.27 | 0.43 | 0.52 |  |  |  |  |  |  |  |
|  | **40** |  |  |  |  |  |  |  | 0.00 | 0.01 | 0.08 | 0.19 | 0.32 | 0.49 | 0.59 |  |  |  |  |  |  |  |
|  | **50** | 0.00 | 0.01 | 0.04 | 0.09 | 0.19 | 0.41 | 0.63 | 0.00 | 0.02 | 0.09 | 0.21 | 0.34 | 0.50 | 0.60 |  |  |  |  |  |  |  |
|  | **60** | 0.00 | 0.01 | 0.04 | 0.10 | 0.21 | 0.45 | 0.70 | 0.00 | 0.02 | 0.11 | 0.22 | 0.35 | 0.52 | 0.62 |  |  |  |  |  |  |  |
|  | **70** | 0.00 | 0.01 | 0.05 | 0.12 | 0.23 | 0.51 | 0.78 | 0.00 | 0.02 | 0.12 | 0.24 | 0.39 | 0.58 | 0.69 |  |  |  |  |  |  |  |
|  | **80** | 0.00 | 0.01 | 0.05 | 0.13 | 0.26 | 0.56 | 0.87 | 0.01 | 0.03 | 0.15 | 0.28 | 0.43 | 0.62 | 0.73 |  |  |  |  |  |  |  |
|  | **90** | 0.00 | 0.01 | 0.06 | 0.14 | 0.29 | 0.62 | 0.95 |  |  |  |  |  |  |  |  |  |  |  |  |  |  |
|  | **100** | 0.00 | 0.01 | 0.06 | 0.16 | 0.31 | 0.68 | 1.04 |  |  |  |  |  |  |  |  |  |  |  |  |  |  |

**Note:** FEV_1_, forced expiratory volume in the first second; CHARLS, the China Health and Retirement Longitudinal Study; NHANES, the National Health and Nutrition Examination Survey; UKB, the UK Biobank.

**Table S12 Age-specific percentile values of multi-systemic aging metrics for participants in the “high-education” group in three datasets**

|  |  | **CHARLS** | | | | | | | **NHANES** | | | | | | | **UKB** | | | | | | |
| --- | --- | --- | --- | --- | --- | --- | --- | --- | --- | --- | --- | --- | --- | --- | --- | --- | --- | --- | --- | --- | --- | --- |
|  | **Age,**  **years** | **1st** | **5th** | **25th** | **50th** | **75th** | **95th** | **99th** | **1st** | **5th** | **25th** | **50th** | **75th** | **95th** | **99th** | **1st** | **5th** | **25th** | **50th** | **75th** | **95th** | **99th** |
| **Cognitive function score** | **50** | 5.4 | 8.4 | 11.9 | 13.8 | 15.6 | 17.8 | 19.2 |  |  |  |  |  |  |  | 2.3 | 3.6 | 5.6 | 7.0 | 8.4 | 10.5 | 12.1 |
|  | **60** | 4.4 | 7.5 | 11.1 | 13.2 | 15.0 | 17.3 | 18.8 | 6.5 | 9.4 | 14.1 | 17.7 | 21.6 | 27.7 | 32.3 | 2.5 | 3.8 | 5.6 | 6.9 | 8.3 | 10.3 | 11.7 |
|  | **70** | 4.3 | 7.3 | 11.0 | 13.0 | 14.9 | 17.2 | 18.6 | 5.5 | 8.2 | 12.8 | 16.3 | 20.1 | 26.1 | 30.7 | 2.5 | 3.7 | 5.6 | 6.9 | 8.2 | 10.2 | 11.6 |
|  | **80** | 2.9 | 5.7 | 9.5 | 11.8 | 13.7 | 16.1 | 17.6 | 4.7 | 7.0 | 10.9 | 13.9 | 17.2 | 22.3 | 26.2 | 2.0 | 3.1 | 4.8 | 6.0 | 7.2 | 9.0 | 10.4 |
|  | **90** | 2.0 | 4.3 | 8.0 | 10.4 | 12.4 | 14.9 | 16.5 |  |  |  |  |  |  |  |  |  |  |  |  |  |  |
|  | **100** | 1.5 | 3.3 | 6.8 | 9.0 | 11.1 | 13.6 | 15.1 |  |  |  |  |  |  |  |  |  |  |  |  |  |  |
| **Depression score** | **20** |  |  |  |  |  |  |  | 0.0 | 0.2 | 1.0 | 2.3 | 4.7 | 10.1 | 15.5 |  |  |  |  |  |  |  |
|  | **30** |  |  |  |  |  |  |  | 0.0 | 0.2 | 0.9 | 2.1 | 4.2 | 9.1 | 14.0 |  |  |  |  |  |  |  |
|  | **40** |  |  |  |  |  |  |  | 0.0 | 0.2 | 0.9 | 2.1 | 4.3 | 9.2 | 14.2 |  |  |  |  |  |  |  |
|  | **50** | 0.0 | 0.0 | 0.4 | 2.6 | 8.0 | 17.4 | 23.1 | 0.0 | 0.2 | 1.0 | 2.3 | 4.6 | 9.9 | 15.3 |  |  |  |  |  |  |  |
|  | **60** | 0.0 | 0.0 | 0.5 | 2.9 | 8.4 | 17.6 | 23.2 | 0.0 | 0.2 | 1.0 | 2.3 | 4.6 | 10.0 | 15.4 |  |  |  |  |  |  |  |
|  | **70** | 0.0 | 0.0 | 0.4 | 2.8 | 8.5 | 18.1 | 24.1 | 0.0 | 0.2 | 0.9 | 2.1 | 4.1 | 9.0 | 13.8 |  |  |  |  |  |  |  |
|  | **80** | 0.0 | 0.0 | 0.5 | 3.0 | 8.5 | 17.7 | 23.3 | 0.0 | 0.1 | 0.8 | 1.9 | 3.9 | 8.4 | 12.9 |  |  |  |  |  |  |  |
|  | **90** | 0.0 | 0.0 | 0.3 | 2.2 | 7.0 | 15.5 | 20.6 |  |  |  |  |  |  |  |  |  |  |  |  |  |  |
|  | **100** | 0.0 | 0.0 | 0.1 | 1.4 | 5.4 | 13.3 | 18.2 |  |  |  |  |  |  |  |  |  |  |  |  |  |  |
| **Systolic blood pressure, mmHg** | **20** |  |  |  |  |  |  |  | 90.4 | 96.6 | 105.6 | 112.3 | 119.7 | 132.6 | 144.5 |  |  |  |  |  |  |  |
|  | **30** |  |  |  |  |  |  |  | 90.1 | 96.8 | 106.6 | 114.0 | 122.4 | 137.2 | 151.0 |  |  |  |  |  |  |  |
|  | **40** |  |  |  |  |  |  |  | 90.9 | 98.2 | 109.2 | 117.6 | 127.1 | 144.2 | 160.6 |  |  |  |  |  |  |  |
|  | **50** | 90.6 | 98.4 | 111.4 | 121.9 | 133.9 | 154.5 | 171.9 | 92.5 | 100.6 | 112.9 | 122.4 | 133.3 | 153.3 | 172.9 | 96.8 | 104.9 | 117.4 | 127.0 | 137.5 | 154.5 | 168.2 |
|  | **60** | 93.0 | 101.4 | 115.4 | 127.0 | 140.3 | 163.3 | 183.0 | 94.6 | 103.4 | 116.9 | 127.5 | 139.7 | 162.6 | 185.3 | 100.4 | 109.3 | 123.1 | 133.8 | 145.5 | 164.6 | 180.1 |
|  | **70** | 94.4 | 103.4 | 118.6 | 131.2 | 145.8 | 171.4 | 193.5 | 96.4 | 105.9 | 120.5 | 132.0 | 145.5 | 171.0 | 196.8 | 105.3 | 114.9 | 129.9 | 141.4 | 154.1 | 175.0 | 192.0 |
|  | **80** | 94.7 | 104.4 | 121.0 | 134.8 | 151.2 | 180.3 | 206.1 | 96.9 | 107.3 | 123.5 | 136.5 | 152.0 | 182.0 | 213.2 | 111.3 | 121.5 | 137.4 | 149.7 | 163.2 | 185.5 | 203.5 |
|  | **90** | 94.0 | 104.4 | 122.6 | 138.0 | 156.6 | 190.4 | 220.9 |  |  |  |  |  |  |  |  |  |  |  |  |  |  |
|  | **100** | 93.6 | 104.8 | 124.4 | 141.4 | 162.1 | 200.6 | 236.2 |  |  |  |  |  |  |  |  |  |  |  |  |  |  |
| **Diastolic blood pressure, mmHg** | **20** |  |  |  |  |  |  |  | 43.5 | 50.5 | 59.5 | 65.5 | 71.7 | 82.2 | 91.5 |  |  |  |  |  |  |  |
|  | **30** |  |  |  |  |  |  |  | 46.9 | 54.4 | 64.0 | 70.4 | 77.1 | 88.2 | 98.2 |  |  |  |  |  |  |  |
|  | **40** |  |  |  |  |  |  |  | 49.7 | 57.6 | 67.7 | 74.4 | 81.5 | 93.2 | 103.7 |  |  |  |  |  |  |  |
|  | **50** | 53.3 | 59.4 | 68.9 | 76.2 | 84.2 | 97.3 | 107.8 | 50.9 | 59.0 | 69.1 | 75.9 | 83.0 | 94.8 | 105.4 | 56.8 | 62.7 | 71.6 | 78.2 | 85.1 | 95.7 | 103.7 |
|  | **60** | 53.5 | 59.5 | 69.0 | 76.2 | 84.2 | 97.1 | 107.6 | 48.5 | 56.7 | 67.1 | 74.2 | 81.5 | 93.7 | 104.8 | 57.7 | 63.5 | 72.3 | 78.8 | 85.7 | 96.1 | 104.0 |
|  | **70** | 52.0 | 57.8 | 66.7 | 73.5 | 81.0 | 93.1 | 102.9 | 43.6 | 52.0 | 62.8 | 70.1 | 77.7 | 90.6 | 102.2 | 56.5 | 62.4 | 71.3 | 77.9 | 84.8 | 95.4 | 103.3 |
|  | **80** | 48.1 | 54.0 | 63.3 | 70.5 | 78.4 | 91.5 | 102.1 | 38.4 | 46.9 | 57.8 | 65.3 | 73.1 | 86.4 | 98.5 | 54.5 | 60.6 | 70.0 | 76.9 | 84.2 | 95.4 | 103.9 |
|  | **90** | 44.5 | 50.7 | 60.5 | 68.2 | 76.8 | 91.1 | 103.0 |  |  |  |  |  |  |  |  |  |  |  |  |  |  |
|  | **100** | 41.3 | 47.6 | 57.9 | 66.1 | 75.3 | 90.9 | 103.9 |  |  |  |  |  |  |  |  |  |  |  |  |  |  |
| **Pulse** | **20** |  |  |  |  |  |  |  | 50.6 | 56.5 | 65.6 | 72.7 | 80.7 | 94.6 | 106.8 |  |  |  |  |  |  |  |
|  | **30** |  |  |  |  |  |  |  | 50.7 | 56.4 | 65.4 | 72.3 | 80.1 | 93.6 | 105.4 |  |  |  |  |  |  |  |
|  | **40** |  |  |  |  |  |  |  | 50.3 | 56.0 | 65.0 | 71.9 | 79.7 | 93.2 | 105.1 |  |  |  |  |  |  |  |
|  | **50** | 53.7 | 59.1 | 67.4 | 73.7 | 80.7 | 92.4 | 102.3 | 49.1 | 54.9 | 64.0 | 71.0 | 79.0 | 92.9 | 105.2 | 47.5 | 53.5 | 62.9 | 70.3 | 78.7 | 93.3 | 105.9 |
|  | **60** | 51.7 | 57.4 | 66.0 | 72.7 | 80.2 | 92.8 | 103.6 | 47.7 | 53.5 | 62.6 | 69.7 | 77.8 | 91.8 | 104.3 | 46.5 | 52.3 | 61.4 | 68.7 | 76.9 | 91.0 | 103.4 |
|  | **70** | 50.8 | 56.4 | 65.1 | 71.9 | 79.4 | 92.1 | 103.1 | 46.9 | 52.6 | 61.5 | 68.4 | 76.3 | 90.0 | 102.2 | 46.0 | 51.8 | 61.0 | 68.3 | 76.6 | 90.9 | 103.4 |
|  | **80** | 50.4 | 56.1 | 64.8 | 71.6 | 79.1 | 91.9 | 103.0 | 46.3 | 51.9 | 60.6 | 67.3 | 75.0 | 88.3 | 100.1 | 45.8 | 51.3 | 60.1 | 67.0 | 74.8 | 88.2 | 99.8 |
|  | **90** | 51.7 | 57.0 | 65.1 | 71.3 | 78.1 | 89.6 | 99.4 |  |  |  |  |  |  |  |  |  |  |  |  |  |  |
|  | **100** | 53.0 | 57.9 | 65.4 | 71.0 | 77.1 | 87.4 | 96.0 |  |  |  |  |  |  |  |  |  |  |  |  |  |  |
| **Body mass index, kg/m^2^** | **20** |  |  |  |  |  |  |  | 15.8 | 18.0 | 21.9 | 25.6 | 30.5 | 40.6 | 51.5 |  |  |  |  |  |  |  |
|  | **30** |  |  |  |  |  |  |  | 17.2 | 19.5 | 23.7 | 27.6 | 32.6 | 43.0 | 54.0 |  |  |  |  |  |  |  |
|  | **40** |  |  |  |  |  |  |  | 18.1 | 20.5 | 24.8 | 28.7 | 33.8 | 44.1 | 54.9 |  |  |  |  |  |  |  |
|  | **50** | 17.7 | 19.5 | 22.3 | 24.4 | 26.8 | 30.7 | 34.0 | 18.4 | 20.8 | 25.1 | 29.0 | 34.1 | 44.4 | 55.0 | 18.5 | 20.3 | 23.3 | 25.9 | 29.0 | 35.2 | 41.6 |
|  | **60** | 17.1 | 18.9 | 21.7 | 23.8 | 26.2 | 30.2 | 33.4 | 18.5 | 20.8 | 25.1 | 28.9 | 33.9 | 43.9 | 54.2 | 18.6 | 20.4 | 23.3 | 25.8 | 28.9 | 34.8 | 40.9 |
|  | **70** | 16.5 | 18.4 | 21.3 | 23.6 | 26.1 | 30.2 | 33.7 | 18.7 | 20.9 | 24.9 | 28.5 | 32.9 | 41.7 | 50.5 | 18.8 | 20.5 | 23.3 | 25.7 | 28.5 | 33.9 | 39.3 |
|  | **80** | 15.2 | 17.1 | 20.1 | 22.4 | 25.1 | 29.6 | 33.4 | 18.4 | 20.4 | 24.0 | 27.1 | 30.9 | 38.1 | 45.1 | 18.8 | 20.4 | 22.9 | 25.0 | 27.4 | 32.0 | 36.5 |
|  | **90** | 14.3 | 16.1 | 18.9 | 21.0 | 23.5 | 27.6 | 31.0 |  |  |  |  |  |  |  |  |  |  |  |  |  |  |
|  | **100** | 13.5 | 15.1 | 17.6 | 19.6 | 21.8 | 25.4 | 28.5 |  |  |  |  |  |  |  |  |  |  |  |  |  |  |
| **Waist circumference, cm** | **20** |  |  |  |  |  |  |  | 60.1 | 66.5 | 77.8 | 87.4 | 99.1 | 120.7 | 140.6 |  |  |  |  |  |  |  |
|  | **30** |  |  |  |  |  |  |  | 65.3 | 72.1 | 83.8 | 93.8 | 105.8 | 127.7 | 147.7 |  |  |  |  |  |  |  |
|  | **40** |  |  |  |  |  |  |  | 69.0 | 75.8 | 87.7 | 97.7 | 109.7 | 131.3 | 150.9 |  |  |  |  |  |  |  |
|  | **50** | 58.0 | 69.3 | 81.0 | 87.7 | 94.2 | 103.9 | 112.0 | 71.0 | 78.0 | 89.8 | 99.8 | 111.6 | 132.9 | 152.0 | 61.1 | 67.4 | 77.5 | 85.4 | 94.2 | 108.6 | 120.1 |
|  | **60** | 57.8 | 69.2 | 81.0 | 87.8 | 94.3 | 104.1 | 112.3 | 72.7 | 79.6 | 91.3 | 101.1 | 112.6 | 133.2 | 151.5 | 61.6 | 67.9 | 78.2 | 86.3 | 95.2 | 109.8 | 121.5 |
|  | **70** | 57.3 | 69.2 | 81.5 | 88.6 | 95.3 | 105.5 | 113.8 | 74.5 | 81.1 | 92.1 | 101.3 | 111.9 | 130.5 | 146.6 | 63.4 | 69.7 | 79.8 | 87.8 | 96.6 | 110.9 | 122.2 |
|  | **80** | 55.3 | 67.5 | 80.0 | 87.2 | 94.1 | 104.4 | 112.9 | 74.6 | 80.8 | 90.9 | 99.3 | 108.8 | 125.3 | 139.3 | 65.0 | 71.0 | 80.7 | 88.3 | 96.6 | 110.0 | 120.6 |
|  | **90** | 55.3 | 66.6 | 78.4 | 85.1 | 91.6 | 101.3 | 109.4 |  |  |  |  |  |  |  |  |  |  |  |  |  |  |
|  | **100** | 55.4 | 65.9 | 76.7 | 83.0 | 89.0 | 98.1 | 105.7 |  |  |  |  |  |  |  |  |  |  |  |  |  |  |
| **Grip strength, kg** | **20** |  |  |  |  |  |  |  | 18.5 | 23.4 | 31.9 | 39.0 | 47.2 | 61.2 | 72.6 |  |  |  |  |  |  |  |
|  | **30** |  |  |  |  |  |  |  | 19.1 | 24.2 | 32.9 | 40.3 | 48.7 | 63.1 | 74.8 |  |  |  |  |  |  |  |
|  | **40** |  |  |  |  |  |  |  | 19.3 | 24.2 | 32.6 | 39.6 | 47.7 | 61.3 | 72.4 |  |  |  |  |  |  |  |
|  | **50** | 13.5 | 19.8 | 29.1 | 35.8 | 42.7 | 52.9 | 60.2 | 18.7 | 23.4 | 31.4 | 38.0 | 45.6 | 58.4 | 68.8 | 14.2 | 19.2 | 27.7 | 34.7 | 42.5 | 55.2 | 65.3 |
|  | **60** | 14.2 | 19.9 | 28.3 | 34.4 | 40.6 | 49.7 | 56.3 | 17.4 | 21.8 | 29.5 | 35.8 | 43.1 | 55.5 | 65.5 | 12.5 | 17.1 | 25.2 | 31.8 | 39.3 | 51.5 | 61.2 |
|  | **70** | 12.4 | 17.7 | 25.4 | 31.0 | 36.8 | 45.3 | 51.3 | 15.2 | 19.3 | 26.4 | 32.4 | 39.3 | 51.0 | 60.6 | 11.4 | 15.9 | 23.7 | 30.1 | 37.4 | 49.4 | 58.9 |
|  | **80** | 10.8 | 15.6 | 22.9 | 28.1 | 33.4 | 41.3 | 47.0 | 11.9 | 15.4 | 21.7 | 27.1 | 33.3 | 44.1 | 53.0 | 11.0 | 15.1 | 22.1 | 27.9 | 34.3 | 45.0 | 53.3 |
|  | **90** | 10.1 | 14.3 | 20.5 | 25.0 | 29.6 | 36.4 | 41.3 |  |  |  |  |  |  |  |  |  |  |  |  |  |  |
|  | **100** | 9.4 | 12.9 | 18.0 | 21.7 | 25.5 | 31.1 | 35.1 |  |  |  |  |  |  |  |  |  |  |  |  |  |  |
| **FEV_1_, ml** | **20** |  |  |  |  |  |  |  | 2173.57 | 2580.03 | 3232.90 | 3739.95 | 4292.22 | 5168.71 | 5843.89 |  |  |  |  |  |  |  |
|  | **30** |  |  |  |  |  |  |  | 2006.16 | 2407.89 | 3058.00 | 3566.13 | 4122.11 | 5008.83 | 5694.86 |  |  |  |  |  |  |  |
|  | **40** |  |  |  |  |  |  |  | 1737.40 | 2120.87 | 2748.51 | 3243.74 | 3789.28 | 4665.71 | 5348.07 |  |  |  |  |  |  |  |
|  | **50** |  |  |  |  |  |  |  | 1475.59 | 1826.70 | 2406.77 | 2868.01 | 3378.89 | 4204.40 | 4850.35 | 1449.29 | 1870.06 | 2534.05 | 3039.26 | 3580.27 | 4422.90 | 5062.01 |
|  | **60** |  |  |  |  |  |  |  | 1234.77 | 1558.89 | 2101.32 | 2537.22 | 3023.62 | 3815.71 | 4439.68 | 1251.15 | 1637.41 | 2250.40 | 2718.72 | 3221.52 | 4006.62 | 4603.31 |
|  | **70** |  |  |  |  |  |  |  | 1015.14 | 1312.29 | 1817.36 | 2228.29 | 2690.78 | 3450.70 | 4053.89 | 1025.91 | 1388.82 | 1972.45 | 2422.55 | 2908.61 | 3671.87 | 4254.60 |
|  | **80** |  |  |  |  |  |  |  | 873.57 | 1135.86 | 1583.35 | 1948.54 | 2360.38 | 3038.52 | 3577.76 | 760.02 | 1094.90 | 1646.41 | 2078.58 | 2549.81 | 3296.52 | 3870.72 |
| **Peak expiratory flow, ml/s** | **20** |  |  |  |  |  |  |  | 4769.9 | 5911.2 | 7650.3 | 8936.3 | 10286.2 | 12345.3 | 13879.4 |  |  |  |  |  |  |  |
|  | **30** |  |  |  |  |  |  |  | 4554.6 | 5753.8 | 7592.9 | 8959.1 | 10397.6 | 12598.4 | 14242.0 |  |  |  |  |  |  |  |
|  | **40** |  |  |  |  |  |  |  | 4116.3 | 5328.8 | 7203.2 | 8603.8 | 10083.6 | 12355.6 | 14057.2 |  |  |  |  |  |  |  |
|  | **50** | 2132.1 | 3068.5 | 4637.2 | 5881.4 | 7246.1 | 9412.3 | 11071.9 | 3603.9 | 4773.8 | 6595.7 | 7964.0 | 9414.4 | 11648.0 | 13324.9 | 1799.0 | 3056.4 | 5059.5 | 6564.4 | 8146.4 | 10540.5 | 12297.0 |
|  | **60** | 1956.4 | 2856.5 | 4372.6 | 5579.8 | 6906.7 | 9017.3 | 10636.8 | 2976.2 | 4114.4 | 5910.9 | 7272.4 | 8723.5 | 10969.5 | 12662.6 | 1470.7 | 2634.1 | 4510.9 | 5928.2 | 7421.5 | 9685.7 | 11349.1 |
|  | **70** | 1214.5 | 2052.9 | 3546.0 | 4775.8 | 6153.9 | 8384.3 | 10118.5 | 2238.8 | 3342.9 | 5127.0 | 6499.3 | 7974.5 | 10275.5 | 12020.7 | 947.9 | 2029.2 | 3868.5 | 5282.4 | 6782.9 | 9070.8 | 10757.9 |
|  | **80** | 703.4 | 1411.7 | 2755.3 | 3899.2 | 5203.4 | 7345.9 | 9029.6 | 1588.9 | 2621.2 | 4339.3 | 5683.2 | 7141.1 | 9433.8 | 11183.2 | 508.7 | 1414.7 | 3139.1 | 4507.3 | 5974.9 | 8229.4 | 9899.9 |
|  | **90** | 443.3 | 950.0 | 1935.0 | 2783.2 | 3755.8 | 5361.0 | 6626.6 |  |  |  |  |  |  |  |  |  |  |  |  |  |  |
|  | **100** | 239.6 | 532.5 | 1109.9 | 1610.3 | 2185.8 | 3137.9 | 3889.9 |  |  |  |  |  |  |  |  |  |  |  |  |  |  |
| **Fat-free mass index, kg/m^2^** | **20** |  |  |  |  |  |  |  | 11.7 | 13.2 | 15.7 | 17.8 | 20.2 | 24.4 | 27.9 |  |  |  |  |  |  |  |
|  | **30** |  |  |  |  |  |  |  | 12.5 | 14.0 | 16.6 | 18.8 | 21.2 | 25.4 | 28.9 |  |  |  |  |  |  |  |
|  | **40** |  |  |  |  |  |  |  | 12.7 | 14.3 | 16.9 | 19.1 | 21.5 | 25.8 | 29.3 |  |  |  |  |  |  |  |
|  | **50** |  |  |  |  |  |  |  | 12.8 | 14.3 | 16.9 | 19.0 | 21.3 | 25.4 | 28.7 | 12.7 | 14.0 | 15.9 | 17.3 | 18.9 | 21.7 | 24.0 |
|  | **60** |  |  |  |  |  |  |  | 12.4 | 14.0 | 16.5 | 18.6 | 21.0 | 25.0 | 28.4 | 12.5 | 13.8 | 15.6 | 17.0 | 18.6 | 21.2 | 23.5 |
|  | **70** |  |  |  |  |  |  |  |  |  |  |  |  |  |  | 12.8 | 14.0 | 15.8 | 17.1 | 18.6 | 21.0 | 23.1 |
|  | **80** |  |  |  |  |  |  |  |  |  |  |  |  |  |  | 13.3 | 14.3 | 15.9 | 17.0 | 18.3 | 20.3 | 22.0 |
| **Bone mineral content, kg** | **20** |  |  |  |  |  |  |  | 1.43 | 1.65 | 2.01 | 2.30 | 2.63 | 3.17 | 3.60 |  |  |  |  |  |  |  |
|  | **30** |  |  |  |  |  |  |  | 1.50 | 1.72 | 2.07 | 2.35 | 2.66 | 3.17 | 3.58 |  |  |  |  |  |  |  |
|  | **40** |  |  |  |  |  |  |  | 1.53 | 1.74 | 2.08 | 2.35 | 2.65 | 3.14 | 3.53 |  |  |  |  |  |  |  |
|  | **50** |  |  |  |  |  |  |  | 1.44 | 1.65 | 2.01 | 2.29 | 2.61 | 3.13 | 3.55 | 1.74 | 2.00 | 2.41 | 2.73 | 3.09 | 3.66 | 4.11 |
|  | **60** |  |  |  |  |  |  |  | 1.25 | 1.48 | 1.85 | 2.16 | 2.50 | 3.09 | 3.57 | 1.48 | 1.76 | 2.21 | 2.56 | 2.95 | 3.60 | 4.13 |
|  | **70** |  |  |  |  |  |  |  |  |  |  |  |  |  |  | 1.42 | 1.71 | 2.18 | 2.56 | 2.98 | 3.69 | 4.26 |
|  | **80** |  |  |  |  |  |  |  |  |  |  |  |  |  |  | 1.40 | 1.70 | 2.18 | 2.58 | 3.02 | 3.75 | 4.35 |
| **Bone mineral density, g/cm^2^** | **20** |  |  |  |  |  |  |  | 0.88 | 0.94 | 1.04 | 1.11 | 1.18 | 1.30 | 1.40 |  |  |  |  |  |  |  |
|  | **30** |  |  |  |  |  |  |  | 0.89 | 0.96 | 1.05 | 1.12 | 1.19 | 1.30 | 1.39 |  |  |  |  |  |  |  |
|  | **40** |  |  |  |  |  |  |  | 0.90 | 0.97 | 1.06 | 1.12 | 1.19 | 1.31 | 1.40 |  |  |  |  |  |  |  |
|  | **50** |  |  |  |  |  |  |  | 0.87 | 0.93 | 1.03 | 1.10 | 1.18 | 1.31 | 1.41 | 0.98 | 1.07 | 1.18 | 1.26 | 1.34 | 1.46 | 1.55 |
|  | **60** |  |  |  |  |  |  |  | 0.79 | 0.87 | 0.97 | 1.05 | 1.14 | 1.28 | 1.39 | 0.87 | 0.97 | 1.10 | 1.20 | 1.29 | 1.44 | 1.55 |
|  | **70** |  |  |  |  |  |  |  |  |  |  |  |  |  |  | 0.82 | 0.93 | 1.08 | 1.18 | 1.29 | 1.45 | 1.57 |
|  | **80** |  |  |  |  |  |  |  |  |  |  |  |  |  |  | 0.79 | 0.90 | 1.06 | 1.17 | 1.29 | 1.46 | 1.58 |
| **Frailty index_Lab** | **20** |  |  |  |  |  |  |  | 0.00 | 0.00 | 0.03 | 0.10 | 0.21 | 0.36 | 0.45 |  |  |  |  |  |  |  |
|  | **30** |  |  |  |  |  |  |  | 0.00 | 0.00 | 0.03 | 0.11 | 0.23 | 0.40 | 0.49 |  |  |  |  |  |  |  |
|  | **40** |  |  |  |  |  |  |  | 0.00 | 0.00 | 0.03 | 0.12 | 0.25 | 0.44 | 0.55 |  |  |  |  |  |  |  |
|  | **50** | 0.00 | 0.01 | 0.04 | 0.09 | 0.19 | 0.40 | 0.62 | 0.00 | 0.00 | 0.04 | 0.13 | 0.28 | 0.49 | 0.60 |  |  |  |  |  |  |  |
|  | **60** | 0.00 | 0.01 | 0.04 | 0.10 | 0.21 | 0.45 | 0.69 | 0.00 | 0.00 | 0.05 | 0.15 | 0.31 | 0.53 | 0.65 |  |  |  |  |  |  |  |
|  | **70** | 0.00 | 0.01 | 0.05 | 0.11 | 0.23 | 0.49 | 0.76 | 0.00 | 0.01 | 0.06 | 0.18 | 0.35 | 0.56 | 0.69 |  |  |  |  |  |  |  |
|  | **80** | 0.00 | 0.01 | 0.05 | 0.13 | 0.26 | 0.57 | 0.88 | 0.00 | 0.01 | 0.09 | 0.22 | 0.38 | 0.58 | 0.69 |  |  |  |  |  |  |  |
|  | **90** | 0.00 | 0.01 | 0.06 | 0.13 | 0.27 | 0.58 | 0.89 |  |  |  |  |  |  |  |  |  |  |  |  |  |  |
|  | **100** | 0.00 | 0.01 | 0.05 | 0.13 | 0.26 | 0.57 | 0.88 |  |  |  |  |  |  |  |  |  |  |  |  |  |  |

**Note:** FEV_1_, forced expiratory volume in the first second; CHARLS, the China Health and Retirement Longitudinal Study; NHANES, the National Health and Nutrition Examination Survey; UKB, the UK Biobank.
