# Supplementary material for: Delineating Life‐Course Percentile Curves and Normative Values of Multi‐Systemic Ageing Metrics in the United Kingdom, the United States, and China
Source: J Cachexia Sarcopenia Muscle. 2025 Jun 13;16(3):e13862. doi: 10.1002/jcsm.13862 (PMC12163542; doi:10.1002/jcsm.13862)

**a** Sociodemographic disparities within the United Kingdom, the United States, and China

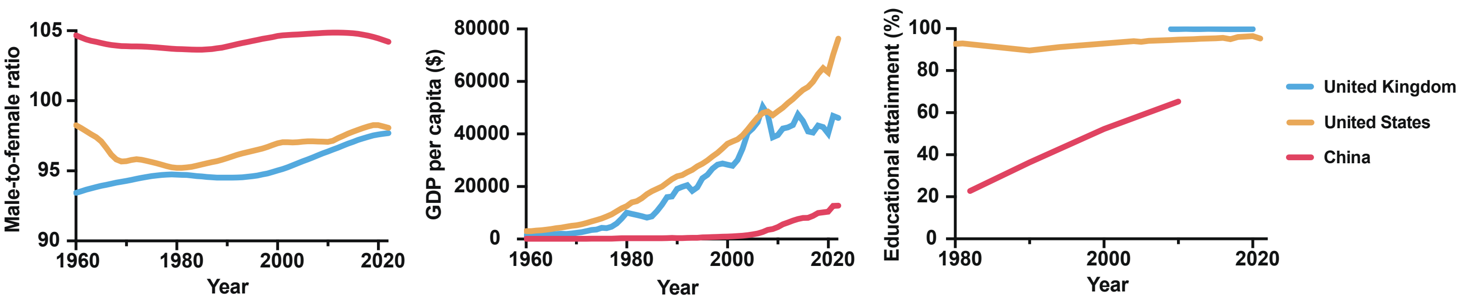


**b** Study population and dataset selection

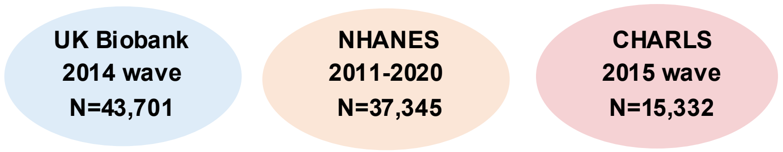


**c** Sociodemographic factors

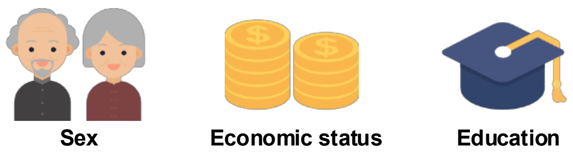


**d** Organ/system representative aging metrics from different dimensions

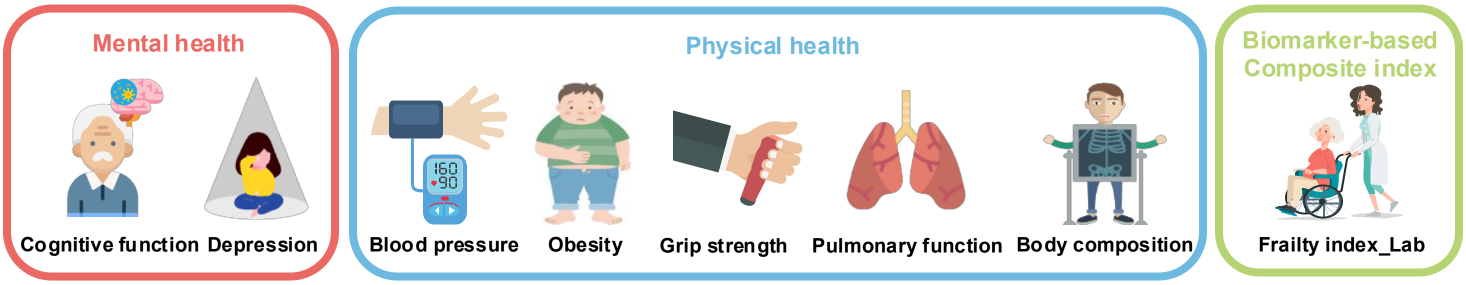


**e** Modelling fitting and graphic plotting

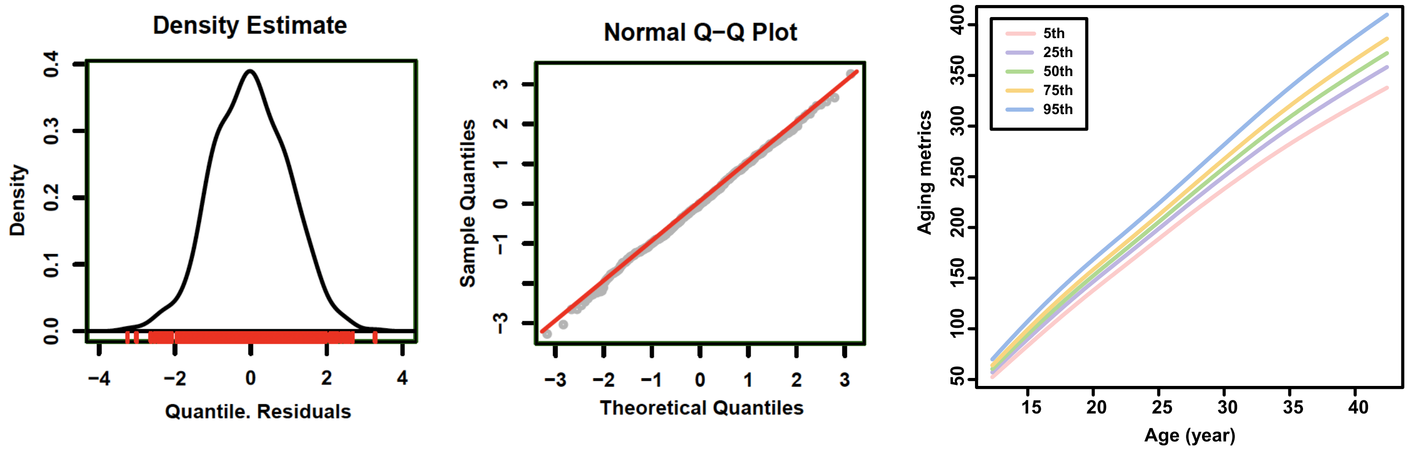

Supplement: Supplementary file 3 — Figure S1 Roadmap of the study design. a. Sociodemographic disparities within the United Kingdom, the United States, and China, including the tendencies of the male‐to‐female ratio, the GDP per capita, and the educational attainment rate for lower secondary schools in adults. b. Three national datasets were utilized for the cross‐national analyses, including the UKB (the United Kingdom, 2014 wave, N = 43 701), the NHANES (the United States, 2011–2020 waves, N = 37 345), and the CHARLS (China, 2015 wave, N = 15 332). c. Three sociodemographic (sex, income, and education) factors were considered in the analysis. d. We selected 14 organ/system representative ageing metrics from different dimensions (i.e., Mental health, Physical health, and Biomarker‐based Composite index). e. Using the GAMLSS method, we fitted the percentile curves for each ageing metric with the advancing age. Several parameters were used to test the residual and the degree of the fitted model. Note: Data utilized in Figure S1a were obtained from the official website of the World Bank (https://data.worldbank.org.cn/?cid=eap_wechat_worldbank_zh_ext). CHARLS, the China Health and Retirement Longitudinal Study; NHANES, the National Health and Nutrition Examination Survey; UKB, the UK Biobank; GAMLSS, Generalized Additive Models for Location, Scale, and Shape. [file JCSM-16-e13862-s004.pdf]
