# Supplementary figures and images for: Delineating Life‐Course Percentile Curves and Normative Values of Multi‐Systemic Ageing Metrics in the United Kingdom, the United States, and China
Source: J Cachexia Sarcopenia Muscle. 2025 Jun 13;16(3):e13862. doi: 10.1002/jcsm.13862 (PMC12163542; doi:10.1002/jcsm.13862)

UKB

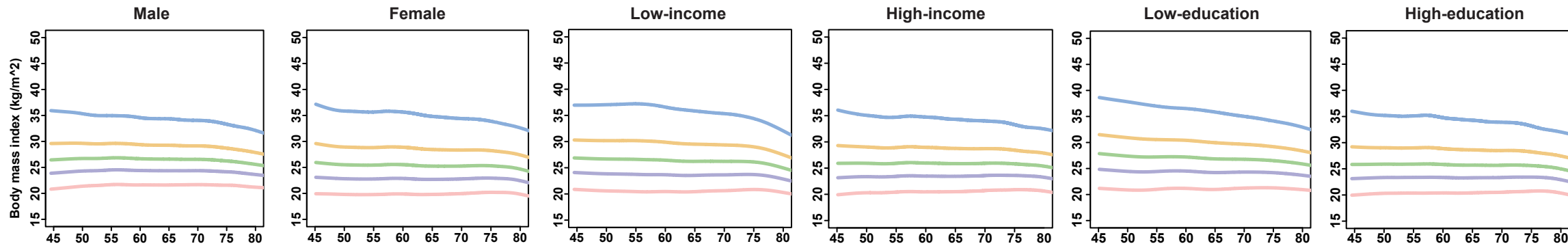

NHANES

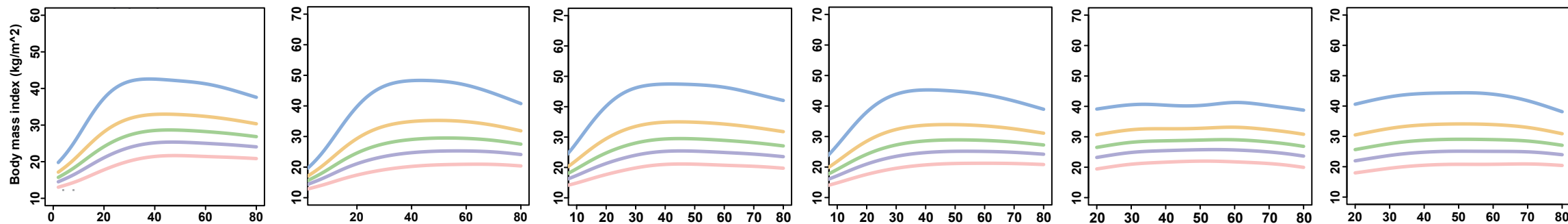

CHARLS

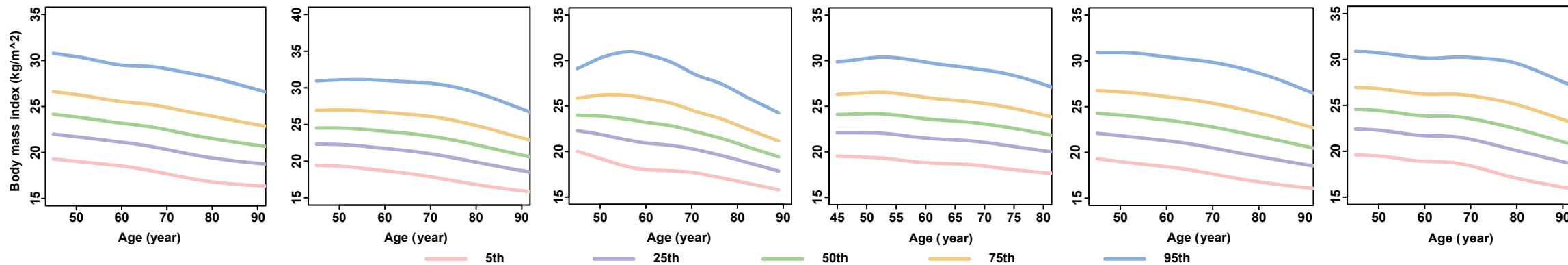

Supplement: Supplementary file 4 — Figure S2 Percentile curves of body mass index by different sociodemographic contexts in three datasets. Note: The solid lines in different colours represent the corresponding percentile curves. UKB, the UK Biobank; NHANES, the National Health and Nutrition Examination Survey; CHARLS, the China Health and Retirement Longitudinal Study. [file JCSM-16-e13862-s010.pdf]

UKB

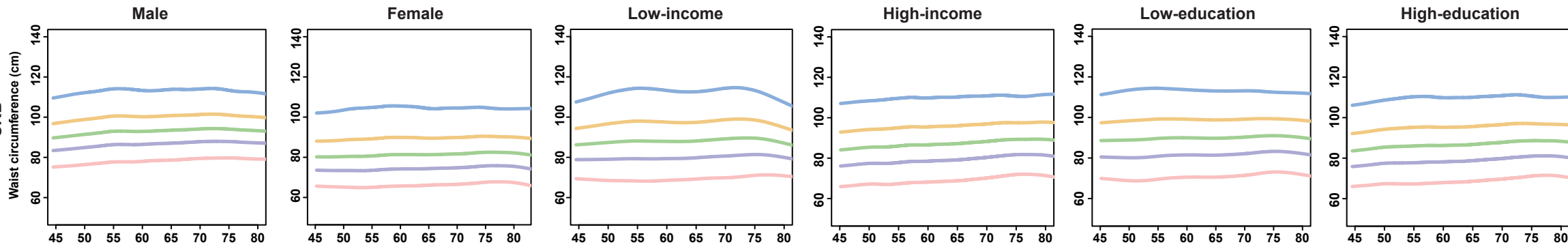

NHANES

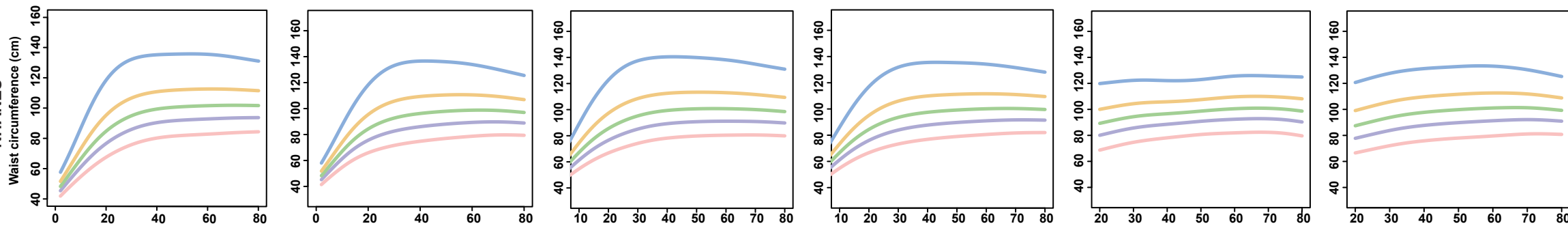

CHARLS

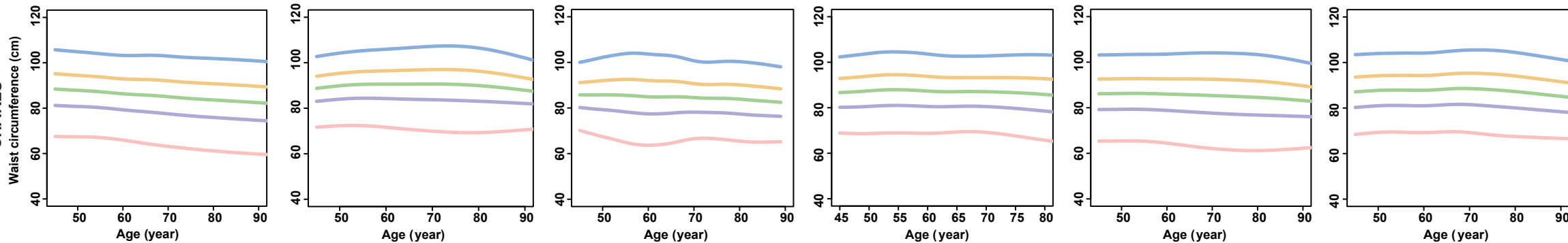

5th

25th

50th

75th

95th

Supplement: Supplementary file 5 — Figure S3 Percentile curves of waist circumference by different sociodemographic contexts in three datasets. Note: The solid lines in different colours represent the corresponding percentile curves. UKB, the UK Biobank; NHANES, the National Health and Nutrition Examination Survey; CHARLS, the China Health and Retirement Longitudinal Study. [file JCSM-16-e13862-s005.pdf]

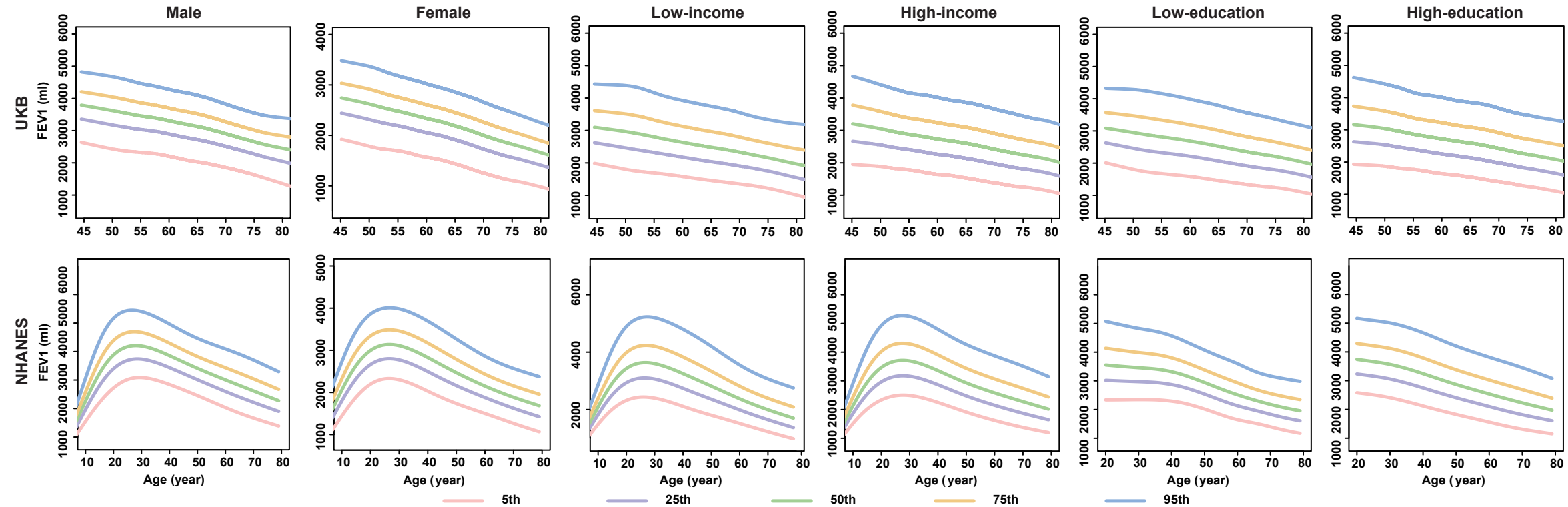

Supplement: Supplementary file 6 — Figure S4 Percentile curves of FEV1 by different sociodemographic contexts in UKB and NHANES. Note: The solid lines in different colours represent the corresponding percentile curves. UKB, the UK Biobank; NHANES, the National Health and Nutrition Examination Survey; FEV1, forced expiratory volume in the first second. [file JCSM-16-e13862-s001.pdf]

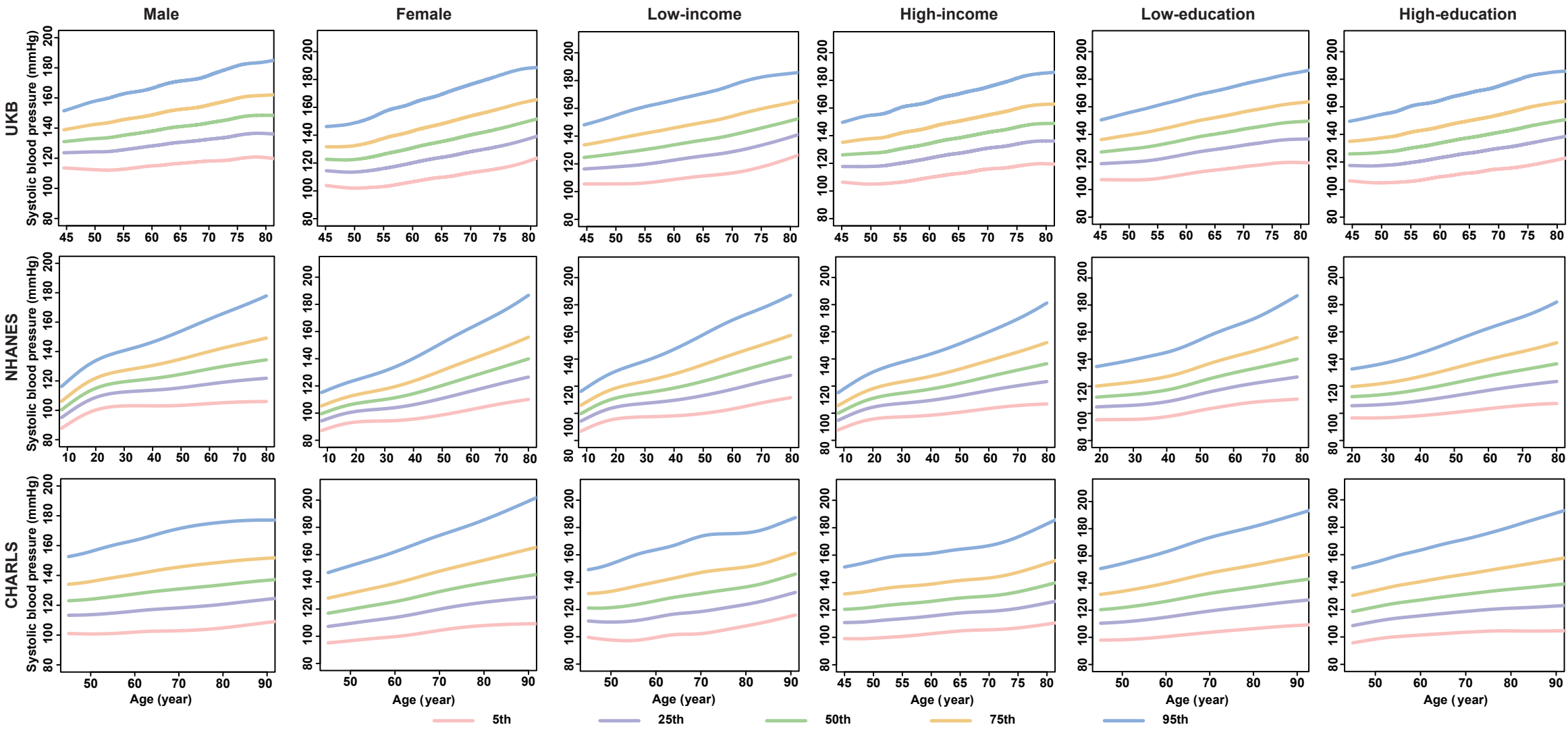

Supplement: Supplementary file 7 — Figure S5 Percentile curves of systolic blood pressure by different sociodemographic contexts in three datasets. Note: The solid lines in different colours represent the corresponding percentile curves. UKB, the UK Biobank; NHANES, the National Health and Nutrition Examination Survey; CHARLS, the China Health and Retirement Longitudinal Study. [file JCSM-16-e13862-s002.pdf]

UKB

Male

Female

Low-income

High-income

Low-education

High-education

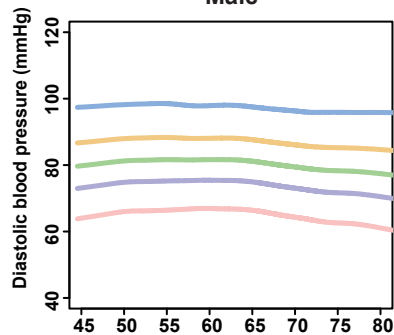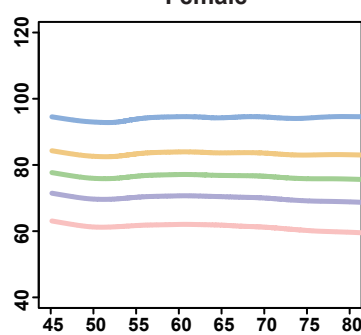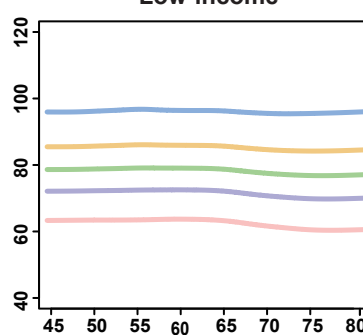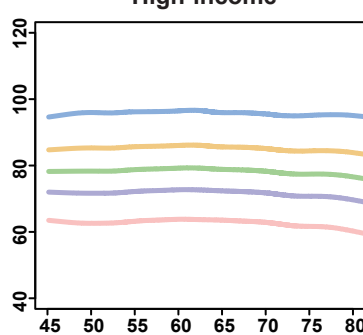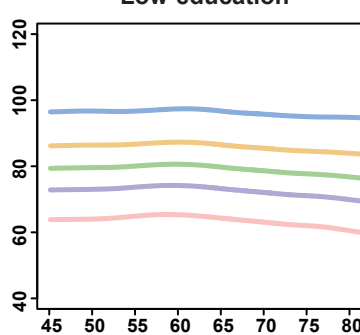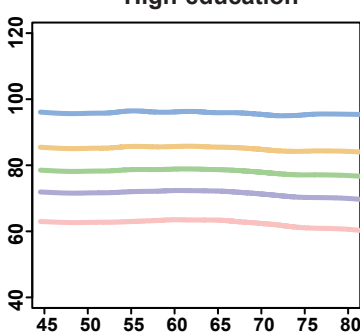

NHANES

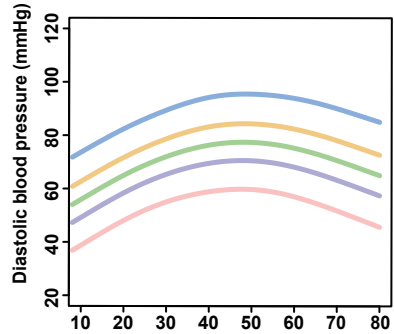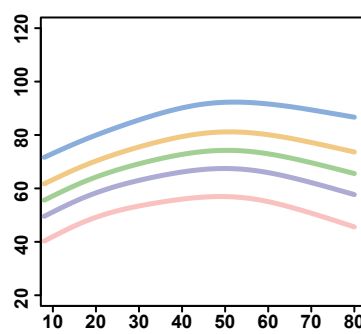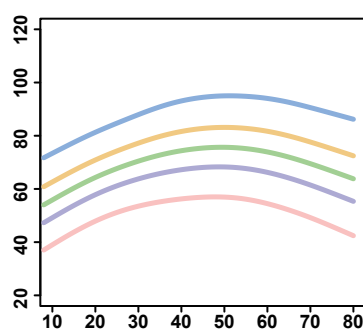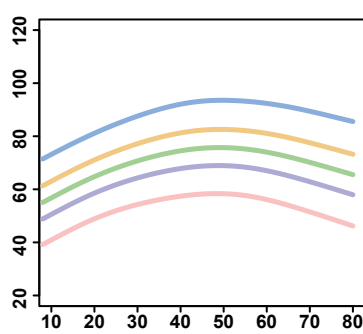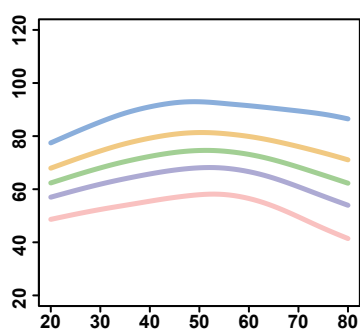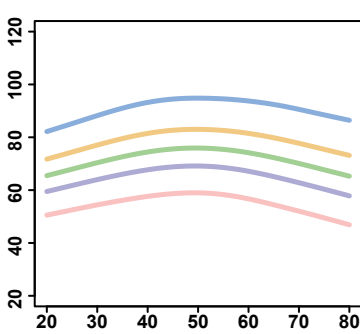

CHARLS

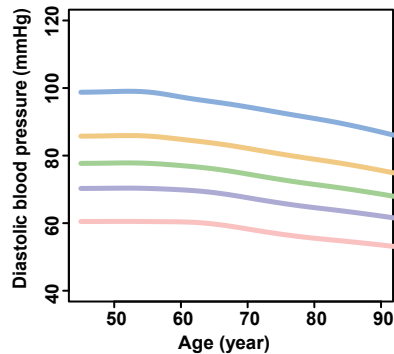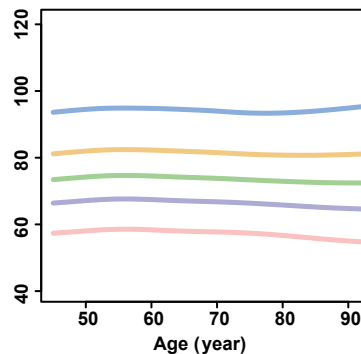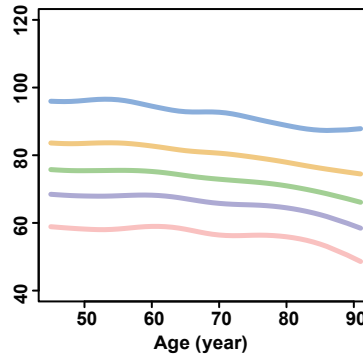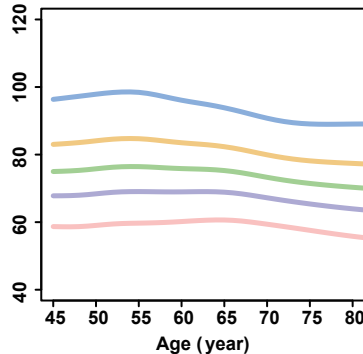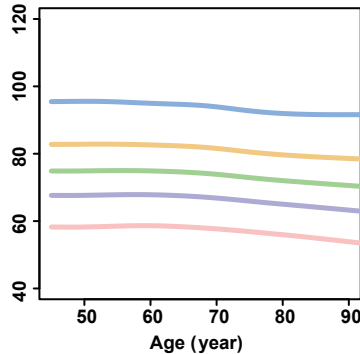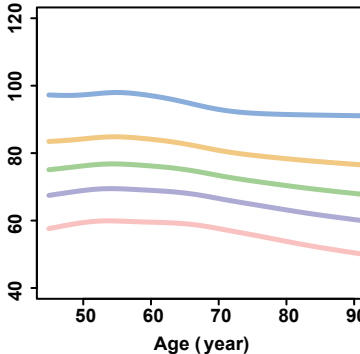

5th

25th

50th

75th

95th

Supplement: Supplementary file 8 — Figure S6 Percentile curves of diastolic blood pressure by different sociodemographic contexts in three datasets. Note: The solid lines in different colours represent the corresponding percentile curves. UKB, the UK Biobank; NHANES, the National Health and Nutrition Examination Survey; CHARLS, the China Health and Retirement Longitudinal Study. [file JCSM-16-e13862-s007.pdf]

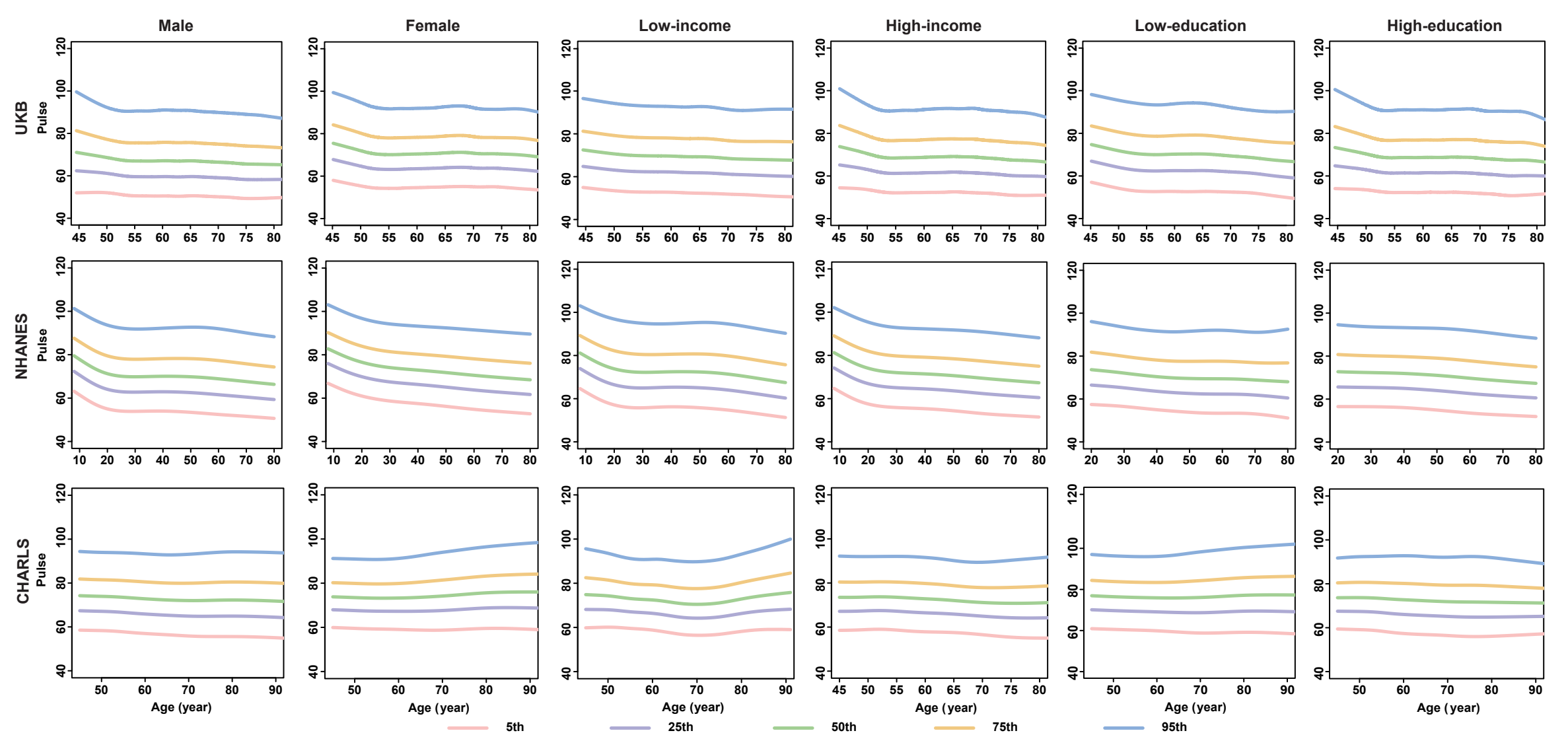

Supplement: Supplementary file 9 — Figure S7 Percentile curves of pulse by different sociodemographic contexts in three datasets. Note: The solid lines in different colours represent the corresponding percentile curves. UKB, the UK Biobank; NHANES, the National Health and Nutrition Examination Survey; CHARLS, the China Health and Retirement Longitudinal Study. [file JCSM-16-e13862-s008.pdf]

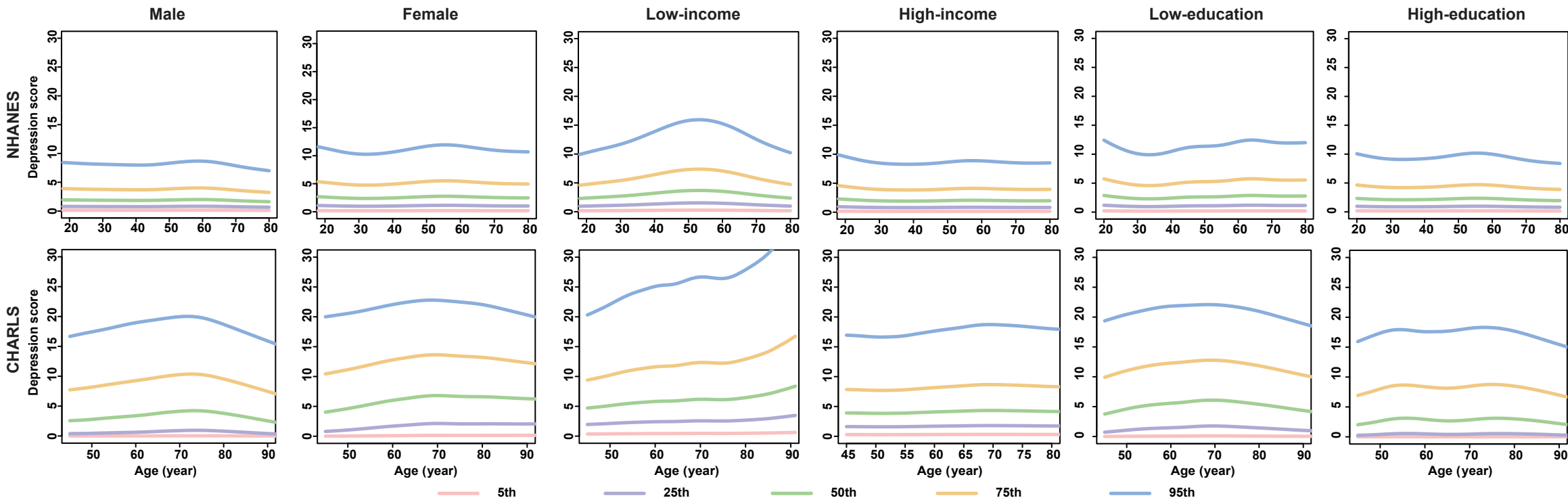

Supplement: Supplementary file 10 — Figure S8 Percentile curves of depression scores by different sociodemographic contexts in CHARLS and NHANES. Note: The solid lines in different colours represent the corresponding percentile curves. NHANES, the National Health and Nutrition Examination Survey; CHARLS, the China Health and Retirement Longitudinal Study. [file JCSM-16-e13862-s011.pdf]

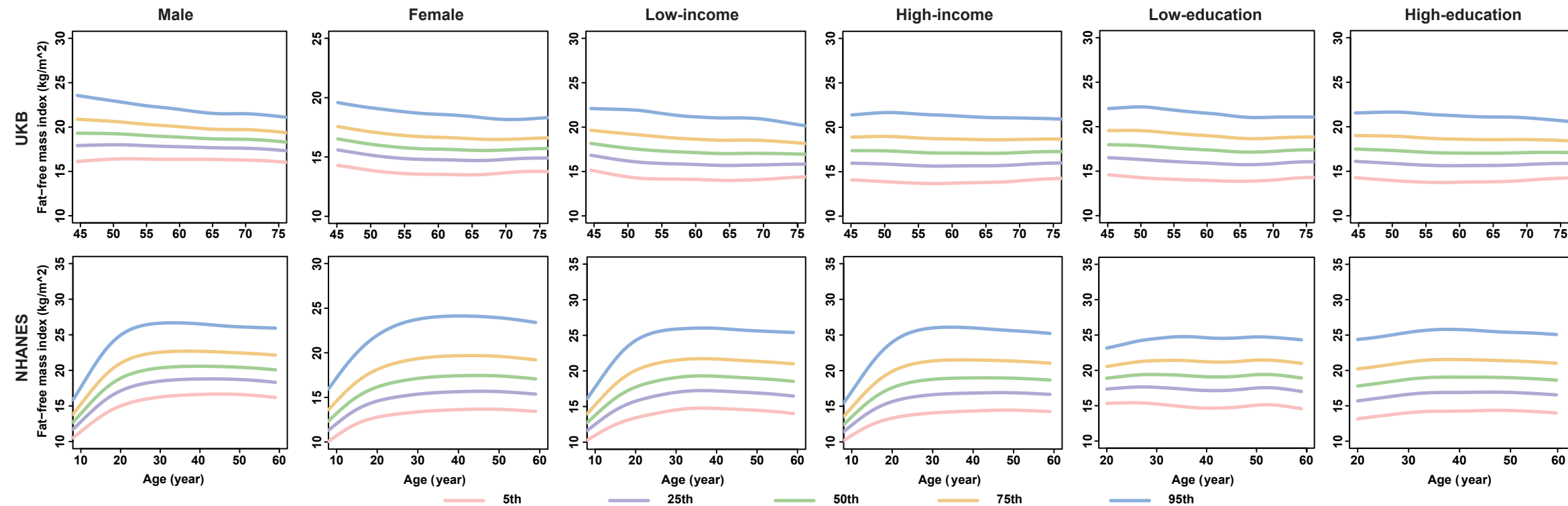

Supplement: Supplementary file 11 — Figure S9 Percentile curves of fat‐free mass index by different sociodemographic contexts in UKB and NHANES. Note: The solid lines in different colours represent the corresponding percentile curves. UKB, the UK Biobank; NHANES, the National Health and Nutrition Examination Survey. [file JCSM-16-e13862-s009.pdf]
